# A multidimensional social risk atlas of depression and anxiety: An observational and genome-wide environmental interaction study

**DOI:** 10.7189/jogh.13.04146

**Published:** 2023-12-08

**Authors:** Chuyu Pan, Li Liu, Shiqiang Cheng, Xuena Yang, Peilin Meng, Na Zhang, Dan He, Yujing Chen, Chun’e Li, Huijie Zhang, Jingxi Zhang, Zhen Zhang, Bolun Cheng, Yan Wen, Yumeng Jia, Huan Liu, Feng Zhang

**Affiliations:** Key Laboratory of Trace Elements and Endemic Diseases of National Health and Family Planning Commission, Key Laboratory of Environment and Genes Related to Diseases of Ministry of Education of China, Key Laboratory for Disease Prevention and Control and Health Promotion of Shaanxi Province, School of Public Health, Health Science Center, Xi’an Jiaotong University, Xi'an, P. R. China

## Abstract

**Background:**

Mental disorders are largely socially determined, yet the combined impact of multidimensional social factors on the two most common mental disorders, depression and anxiety, remains unclear.

**Methods:**

We constructed a polysocial risk score (PsRS), a multidimensional social risk indicator including components from three domains: socioeconomic status, neighborhood and living environment and psychosocial factors. Supported by the UK Biobank cohort, we randomly divided 110 332 participants into the discovery cohort (60%; n = 66 200) and the replication cohort (40%; n = 44 134). We tested the associations between 13 single social factors with Patient Health Questionnaire (PHQ) score, Generalized Anxiety Disorder Scale (GAD) score and self-reported depression and anxiety. The significant social factors were used to calculate PsRS for each mental disorder by considering weights from the multivariable linear model. Generalized linear models were applied to explore the association between PsRS and depression and anxiety. Genome-wide environmental interaction study (GWEIS) was further performed to test the effect of interactions between PsRS and SNPs on the risk of mental phenotypes.

**Results:**

In the discovery cohort, PsRS was positively associated with PHQ score (β *=* 0.37; 95% CI = 0.35-0.38), GAD score (β *=* 0.27; 95% CI = 0.25-0.28), risk of self-reported depression (OR = 1.29; 95% CI = 1.28-1.31) and anxiety (OR = 1.19; 95% CI = 1.19-1.23). Similar results were observed in the replication cohort. Emotional stress, lack of social support and low household income were significantly associated with the development of depression and anxiety. GWEIS identified multiple candidate loci for PHQ score, such as rs149137169 (*ST18*) (*P*_discovery_ = 1.08 × 10^−8^, *P*_replication_ = 3.25 × 10^−6^) and rs3759812 (*MYO9A*) (*P*_discovery_ = 3.87 × 10^−9^, *P*_replication_ = 6.21 × 10^−5^). Additionally, seven loci were detected for GAD score, such as rs114006170 (*TMPRSS11D*) (*P*_discovery_ = 1.14 × 10^−9^, *P*_replication_ = 7.36 × 10^−5^) and rs77927903 (*PIP4K2A*) (*P*_discovery_ = 2.40 × 10^−9^, *P*_replication_ = 0.002).

**Conclusions:**

Our findings reveal the positive effects of multidimensional social factors on the risk of depression and anxiety. It is important to address key social disadvantage in mental health promotion and treatment.

The global burden of disease due to mental disorder has increased in all countries over the past few decades [1]. According to global burden disease (GBD) 2019, mental disorders were the second leading cause of years lived with disability (YLDs), and also the seventh leading cause of disability-adjusted life-years (DALYs) worldwide [2]. Depression and anxiety disorders are the most common mental disorders, accounting for 37.3 and 22.9% of mental disorder DALYs, respectively [1]. As a basic human right, mental health continues to receive attention from the World Health Organization (WHO) [3]. Despite a large number of studies showing that it is possible to take steps to prevent and treat mental disorder and improve mental health, the progress in translating the research findings into realistic benefits is still slow [3].

Substantial evidence supported that social factors significantly influence the risk of depression and anxiety. Healthcare factors play a limited role in health, and health-related behaviours are strongly influenced by social factors [4]. Previous studies have extensively focused on the influence of social risk factors, such as socioeconomic status (SES), psychosocial factors, community and living environment, on the risk of mental disorders. Higher SES can reduce the risk of depression [5] and anxiety [6], alleviate the symptoms of mental disorder and reduce the persistence of mental symptoms [7]. Poor social-emotional support and loneliness will lead to worse symptoms and social functioning and more difficult recovery in people with depression [8]. Structural characteristics of the community, such as environmental stressors and resources, can also affect well-being, depression and anxiety symptoms of residents [9].

However, exploration of etiology is often limited by simply quantifying the contribution of any single factor to health outcomes [10]. Multivariate social risk indicators are helpful in predicting the associations between specific health outcomes risks and comprehensive social level [10]. To date, there were no studies quantifying the contribution of comprehensive social variable to mental disorders. The construction of polysocial risk score (PsRS) [11] will be powerful to explore the more comprehensive domain of social determinants of depression and anxiety disorders.

In recent years, genetic studies have identified a large number of variants that affect the risk of depression and anxiety. However, these variants explained only part of the heritability and were not consistently replicated [12]. Mental illness is the result of the combination of genetic predisposition and environmental risk factors [13]. Gene-environment interaction studies have provided insights and implications in the etiology and treatment of mental disorders [14,15]. Genome-wide environmental interaction study (GWEIS), which considers the impact of environmental exposures on genetically driven disease, has been widely used in psychiatric disorders and has strong efficacy in identifying new risk loci [16-18]. Applying the PsRS to GWEIS may be helpful to prioritise risk variants, or identify novel loci for depressive and anxiety disorders.

In this study, we explored the impact of comprehensive social factor levels on the risk of depression and anxiety by constructing the PsRS in the UK Biobank cohort. GWEIS was further performed to test the interaction effect of the combined social disadvantage and genetic factors to identify genetic loci for the risk of depression and anxiety.

## METHODS

### Study samples and design

UK Biobank is a large-scale population-based prospective study, which consists of biological samples and phenotype data from more than 500 000 people aged 40 to 69 years assessed between 2006 and 2010 in 22 assessment centers throughout the UK. The participants were required to finish the self-completed touch screen questionnaires and brief computer-assisted interviews in the assessment visit. UK Biobank had obtained ethics approval from the North West Multi-center Research Ethics Committee (approval number: 11/NW/0382), and all participants have signed an informed consent, allowing UK Biobank to access their health-related records [19].

In this cross-sectional study, the social risk factors, mental phenotype, covariate data and genotype data were obtained at baseline assessment in the UK Biobank cohort. We excluded non-white individuals and genetically related individuals. Finally, 110 332 participants were included in analysis. Among them, 60% were randomly selected as the discovery cohort, and the remaining 40% were set as the replication cohort. The flowchart of the study are shown in Figure S1 in the **Online Supplementary Document**.

### Definition of mental phenotypes

We used continuous and binary phenotypes to measure depression and anxiety in this study. The depression score was generated based on Patient Health Questionnaire (PHQ)-9, which consists of the 9 items based on the diagnosis criteria of DSM-IV depressive disorders [20]. The 7-item Generalized Anxiety Disorder Scale (GAD-7) was applied for the measurement of continuous indicators of anxiety [21]. For binary phenotypes, the cases of depression and anxiety were defined according to self-reported depression and anxiety, respectively [22]. The participants who didn’t report depressive symptoms and screened negative on PHQ-9 or composite international diagnostic interview short-form (CIDI-SF) [23] were selected as the control group of the self-reported depression, and individuals without self-reported anxiety symptoms didn’t screen positive on GAD-7 were defined as controls of self-reported anxiety. The details of the mental disorder definition are shown in supplementary materials.

### Calculation of PsRS

We selected potential mental health social risk factors from three domains: SES, neighborhood and living environment, and psychosocial factors to generate PsRS [24-27]. The definition of single social factor was based on previous research [11]. The SES domain includes four indicators: low household income (total household income before tax was less than £31 000), low education attainment (education level was lower than college), poor education quality (education score was above the median) and not in paid employment (not in any paid employment or self-employed). Social psychological factors include living alone (number in household = 1), lack of social support (cannot confide in someone nearby at least once a week), social inactivity (attended any group activities less often than once a week), social isolation (visited friends/family or had them to visit once a week or less often) and emotional distress (had experienced illness, injury, bereavement or stress within last two years). Neighborhood and living environment domain consists of area-level material deprivation (Townsend deprivation index was above the median), high local crime rate (crime score for their neighborhood was above the median), poor housing quality (housing score was above the median) and instable accommodation (did not own their current accommodation outright). All indicators were derived from questionnaire interviews with participants and were defined as binary phenotypes. The responses representing higher social risk were scored as “1”, and answers on behalf of lower social risk were scored as “0”. The details definition for social factors are shown in supplementary materials.

The statistically significant social risk factors for each mental trait were selected for further PsRS calculation. First, we identified statistically significant social risk factors for each mental trait. Next, we included these significant risk factors in multivariable models. We then calculated PsRS using the weights derived from the multivariable-adjusted risk estimates (β coefficients) [28]. The formula is as follows:



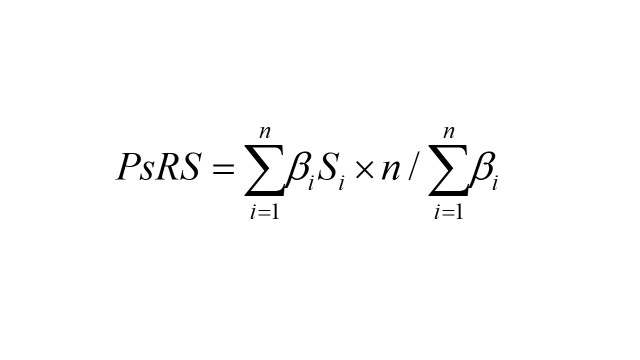


In the formula, n represents the sum of the number of significant social risk factors for mental phenotype, i (i = 1,2,3…n) represents the number of social risk factors, β_i_ represents weights of the ith social risk factor in the multivariable model, S_i_ represents the score of ith significant social risk factor.

### Demographic analysis

Generalised linear model in R 4.1.0 was applied to estimate the effect of single social risk factors and PsRS on mental disorders. Briefly, the linear regression model was used for analyses of PHQ and GAD scores, and logistic model was applied for self-reported depression and anxiety. First, we used multivariate model to identify single social risk factors for each mental phenotype, and the significance threshold was adjusted by Bonferroni correction: *P* = 0.05 / 4 = 0.0125 (four mental phenotypes). The PsRS was then calculated by the sum of significant social indicator score considering the corresponding weights, which has been described previously. Finally, we tested the associations between PsRS and mental phenotypes, and all mental phenotypes were adjusted by age, gender and top 10 PC in regression models.

### UK Biobank genotyping, imputation and quality control

The UK Biobank genetic data comprised genotypes of 488 377 individuals assessed by two very similar genotyping arrays (Applied Biosystems UK BiLEVE Axiom Array and Applied Biosystems UK Biobank Axiom) [29]. The imputation was conducted by IMPUTE4 (https://jmarchini.org/software/) in chunks of approximately 50 000 imputed markers with a 250 kilobase (kb) buffer region and on 5000 samples per compute job. Routine quality control was performed during sample extraction, DNA extraction and genotyping. A series of statistical tests, such as batch effect, plate effect and deviation from Hardy-Weinberg equilibrium (HWE), were conducted to identify poor quality markers for consistency of genotype calling. Principle component analysis (PCA) was computed to account for population structure in both sample-based quality and marker control. KING software was applied to exclude genetically related individuals by performing kinship coefficient estimation. The individuals whose self-reported sex was inconsistent with their genetic sex, who were genotyped but not imputed, and who withdrew the consent were excluded in this study.

### GWEIS analysis

We performed GWEIS analysis to detect the influence of interactions between genetic factors and PsRS on depression and anxiety using the generalised linear models in PLINK 2.0. The SNPs with call rate <0.90, Hardy-Weinberg balance accurate test *P* < 0.001, or minor allele frequency (MAF)<0.01 were excluded for quality control. The mental phenotypes were adjusted by age, gender, and top 10 PCs. In the discovery cohort, the significance threshold of GWEIS was adjusted by Bonferroni correction: *P* = 5.0 × 10^−8^ / 4 = 1.25 × 10^−8^ (four mental phenotypes).

In replication cohort, we performed GWEIS for depression and anxiety by including SNPs with *P* < 1.25 × 10^−8^ and *P* < 1 × 10^−5^ in discovery GWEIS, respectively. SNPs with *P* < 0.05 were considered to be significant in replication analysis. Gene mapping for SNPs was conducted by Varnote-REG function (http://www.mulinlab.org/varnote/application.html#REG).

### Function analysis

We further explored the potential biological function of candidate genes in GWEIS by Functional Mapping and Annotation (FUMA). FUMA is an integrative web-based platform using information from multiple biological resources to facilitate functional annotation in the biological context [30]. The overrepresentation of biological functions of prioritised genes are tested against gene sets obtained from MsigDB and WikiPathways by hypergeometric tests [30].

### Multi-omics-based validation

We retrieved multi-omics databases to validate candidate genes detected by GWEIS, and also to discovery relevant biological functions. GWAScatalog (https://www.ebi.ac.uk/gwas/), TWAS Atlas (https://ngdc.cncb.ac.cn/twas/) and UniPort (https://www.uniprot.org/) was applied for candidate gene confirmation at the genome, transcriptome and proteome level, respectively.

## RESULTS

### Demographic characteristics of participants

Totally, 66 200 and 44 134 individuals were included in the discovery and replication cohorts, respectively. In the discovery cohort, 55 754 and 55 959 individuals were included in analysis of PHQ and GAD score, and the mean ± standard deviation (SD) of PHQ and GAD score were 2.60 ± 3.46 and 2.03 ± 3.22. A total of 53 192 and 49 097 participants were included in analysis of self-reported depression and anxiety, which consisted of 23 374 and 8646 cases, respectively. The detailed basic characteristics of replication cohort samples are shown in **Table 1**.

**Table 1:** Demographic characteristics of participants in the UK Biobank

	Discovery cohort (n total = 66 200)				Replication cohort (n total = 44 134)			
	**PHQ score**	**Self-reported depression**	**GAD score**	**Self-reported anxiety**	**PHQ score**	**Self-reported depression**	**GAD score**	**Self-reported anxiety**
n	55 754	53 192	55 959	49 097	37 038	35 530	37 198	32 673
Case (%)	-	23 374 (43.9)	-	8646 (17.6)	-	15 722 (44.2)	-	5745 (17.6)
Gender = male (%)	25 213 (45.2)	24 138 (45.4)	25 311 (45.2)	22 777 (46.4)	16 915 (45.7)	16 194 (45.6)	16 985 (45.7)	15 339 (46.9)
Age, mean (SD)	55.82 (7.58)	55.99 (7.60)	55.82 (7.58)	56.06 (7.54)	55.84 (7.60)	56.03 (7.63)	55.84 (7.60)	56.07 (7.55)
Accommodation problem = yes (%)	23 567 (42.3)	22 588 (42.5)	23 689 (42.3)	20 283 (41.3)	15 693 (42.4)	15 120 (42.6)	15 781 (42.4)	13 571 (41.5)
Living alone = yes (%)	8874 (15.9)	8607 (16.2)	8916 (15.9)	7834 (16.0)	5999 (16.2)	5841 (16.4)	6040 (16.2)	5339 (16.3)
Social isolation = yes (%)	34 047 (61.1)	31 863 (59.9)	34 199 (61.1)	29 926 (61.0)	22 686 (61.3)	21321 (60.0)	22 785 (61.3)	19 936 (61.0)
Low household income = yes (%)	18 327 (32.9)	19 134 (36.0)	18 375 (32.8)	16 202 (33.0)	12 097 (32.7)	12 716 (35.8)	12 154 (32.7)	10 751 (32.9)
Social support = yes (%)	13 104 (23.5)	12 764 (24.0)	13 170 (23.5)	11 332 (23.1)	8693 (23.5)	8474 (23.9)	8769 (23.6)	7548 (23.1)
Low education attainment = yes (%)	28 076 (50.4)	28 007 (52.7)	28 183 (50.4)	24 687 (50.3)	18 617 (50.3)	18 637 (52.5)	18 697 (50.3)	16 410 (50.2)
Not in paid employment = yes (%)	19 091 (34.2)	19 320 (36.3)	19 130 (34.2)	17 140 (34.9)	12 619 (34.1)	12 900 (36.3)	12 674 (34.1)	11 418 (34.9)
Social inactivity = yes (%)	15 187 (27.2)	14 535 (27.3)	15 260 (27.3)	13 223 (26.9)	10 036 (27.1)	9715 (27.3)	10 097 (27.1)	8720 (26.7)
Emotional distress = yes (%)	22 733 (40.8)	22 461 (42.2)	22 835 (40.8)	19 694 (40.1)	15 018 (40.5)	14 914 (42.0)	15 095 (40.6)	13 029 (39.9)
High local crime rate = yes (%)	23 537 (42.2)	22 705 (42.7)	23 630 (42.2)	20 599 (42.0)	15 762 (42.6)	15 209 (42.8)	15 853 (42.6)	13 767 (42.1)
Poor education quality = yes (%)	21 112 (37.9)	21 003 (39.5)	21 197 (37.9)	18 390 (37.5)	14 111 (38.1)	14 151 (39.8)	14 201 (38.2)	12 315 (37.7)
Poor housing quality = yes (%)	27 693 (49.7)	26 404 (49.6)	27 778 (49.6)	24 342 (49.6)	18 581 (50.2)	17 848 (50.2)	18 649 (50.1)	16 366 (50.1)
Area-level material deprivation = yes (%)	22 617 (40.6)	22 030 (41.4)	22 738 (40.6)	19 752 (40.2)	15 091 (40.7)	14 806 (41.7)	15 176 (40.8)	13 155 (40.3)

PHQ score – patient health questionnaire score, GAD score – generalised anxiety disorder scale score

### Association between single social risk factors and mental phenotypes

**Figure 1:**
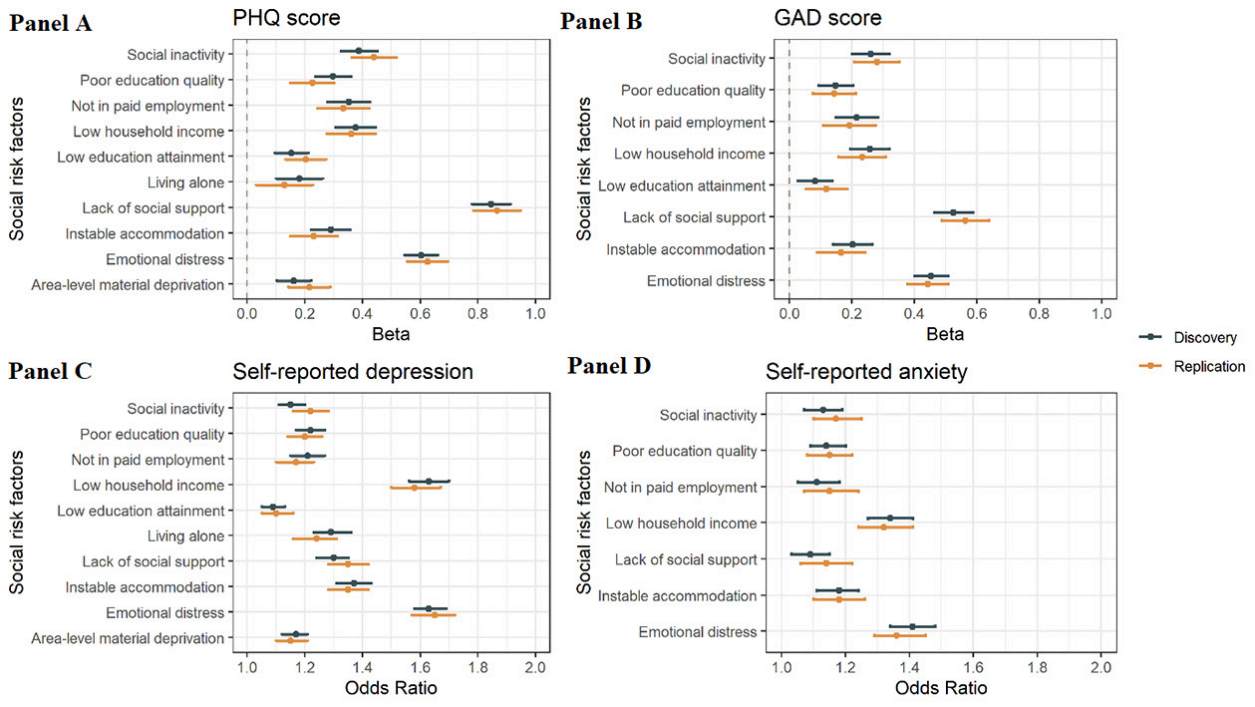
Association between single social factors with depression and anxiety. For PHQ (**Panel A**) and GAD score (**Panel B**), the x-axis shows the beta value, and points display the beta value and 95% confidence intervals (error bars) of beta values. For self-reported depression (**Panel C**) and anxiety (**Panel D**), the x-axis shows the odds ratio, and points display the odds ratios and 95% confidence intervals (error bars) of odd ratios. The y-axis represents the single social factors. PHQ score – patient health questionnaire score, GAD – generalised anxiety disorder scale score

Demographic analyses detected several social risk factors for depression and anxiety (**Figure 1**, panels A-D and Table S2 in the **Online Supplementary Document**). In two cohorts, emotional distress, instable accommodation, lack of social support, low household income, not in paid employment, poor education quality and social inactivity were shared social risk factors for all mental phenotypes. Lack of social support was the leading social risk factor for PHQ score (discovery: β = 0.85 (95% CI = 0.78-0.91); replication: β = 0.87 (95% CI = 0.78-0.95) and GAD score (discovery: β = 0.53 (95% CI = 0.46-0.59); replication: β = 0.56 (95% CI = 0.49-0.64)). Emotional distress was the leading social factor for self-reported depression (discovery: OR = 1.63 (95% CI = 1.58-1.69); replication: OR = 1.65 (95% CI = 1.57-1.72) and anxiety (discovery: OR = 1.41 (95% CI = 1.34-1.48); replication: OR = 1.36 (95% CI = 1.29-1.45).

In discovery and replication cohorts, we also found low education attainment was a significant risk factor for PHQ score, GAD score and self-reported depression. Area-level deprivation and living alone was positively associated with PHQ score and risk of self-reported depression.

### Association between PsRS and mental phenotypes

We calculated PsRS for each mental phenotype using the weight of each significant social risk factor based on multivariable model (**Table 2**). The weights are shown in Table S3 in the **Online Supplementary Document**. The mean ± SD of PsRS for PHQ score, self-reported depression, GAD score and self-reported anxiety were 3.29 ± 1.79, 3.55 ± 1.91, 2.69 ± 1.54, 2.43 ± 1.39 in the discovery cohort and were 3.31 ± 1.81, 3.54 ± 1.89, 2.67 ± 1.55, 2.43 ± 1.38 in the replication cohort. Significant positive associations between PsRS and risk of all mental phenotypes were observed in two cohorts (all *P* < 0.001). In the discovery cohort, PsRS was associated with a 29% increased risk for self-reported depression (95% CI = 1.28-1.31), and a 21% higher risk of self-reported anxiety (95% CI = 1.19-1.23). For one point increase in PsRS, the GAD score will increase by 0.27 points (95% CI = 0.25-0.28) and PHQ score will increase by 0.37 points (95% CI = 0.35-0.38). Similar results were observed in the replication cohort.

**Table 2:** Associations between PsRS and mental phenotypes

Mental phenotype	Components of PsRS	Discovery cohort			Replication cohort			
		**Mean (SD) of PsRS**	Beta/OR* (95% CI)	***P*-value**		**Mean (SD) of PsRS**	Beta/OR* (95% CI)	***P*-value**
PHQ score	(1) Area-level material deprivation, (2) instable accommodation, (3) living alone, (4) low household income, (5) lack of social support, (6) low education attainment, (7) not in paid employment, (8) social inactivity, (9) emotional distress, (10) poor education quality	3.29 ± 1.79	0.37 (0.35-0.38)	<0.001		3.31 ± 1.81	0.36 (0.34-0.38)	<0.001
GAD score	(1) Instable accommodation, (2) low household income, (3) lack of social support, (4) low education attainment, (5) not in paid employment, (6) social inactivity, (7) emotional distress, (8) poor education quality	2.69 ± 1.54	0.27 (0.25-0.28)	<0.001		2.67 ± 1.55	0.27 (0.25-0.29)	<0.001
Self-reported depression	(1) Area-level material deprivation, (2) instable accommodation, (3) living alone, (4) low household income, (5) lack of social support, (6) low education attainment, (7) not in paid employment, (8) social inactivity, (9) emotional distress, (10) poor education quality	3.55 ± 1.91	1.29 (1.28-1.31)	<0.001		3.54 ± 1.89	1.29 (1.27-1.3)	<0.001
Self-reported anxiety	(1) Instable accommodation, (2) low household income, (3) lack of social support, (4) not in paid employment, (5) social inactivity, (6) emotional distress, (7) poor education quality	2.43 ± 1.39	1.21 (1.19-1.23)	<0.001		2.43 ± 1.38	1.21 (1.18-1.23)	<0.001

PsRS – Polysocial risk score, SD – standard deviation, OR – odds ratio, CI – confidence interval, PHQ score – patient health questionnaire score, GAD score – generalised anxiety disorder scale score

*Beta was the estimate of PsRS for PHQ score and GAD score. OR was the estimate for self-reported depression and anxiety.

### Interactions between individual SNPs and PsRS

**Figure 2:**
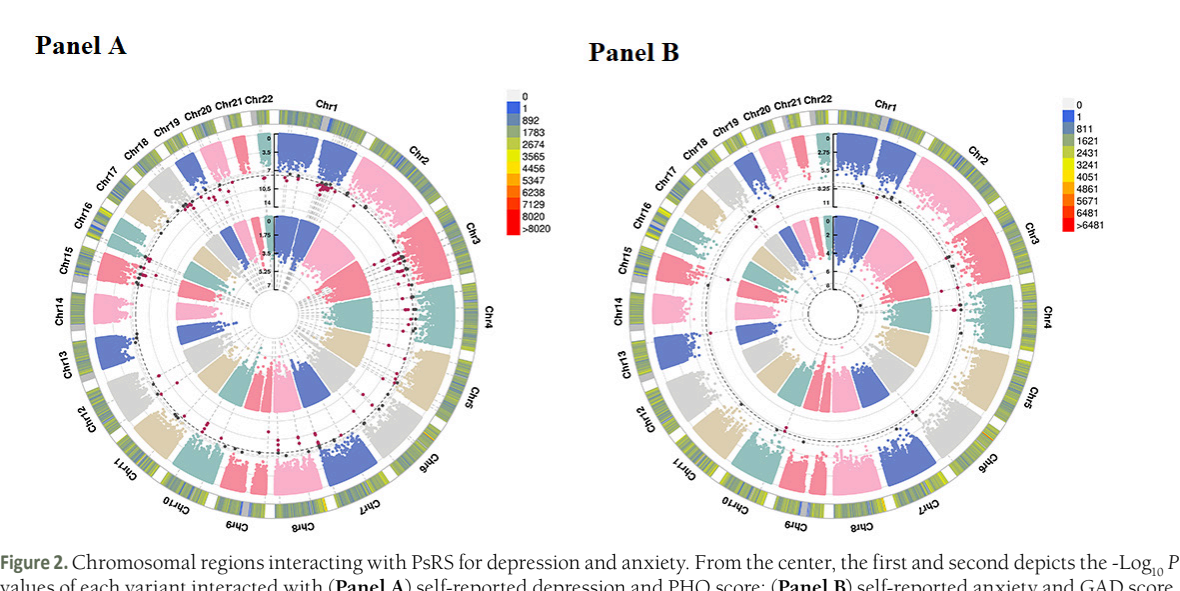
Chromosomal regions interacting with PsRS for depression and anxiety. From the center, the first and second depicts the -Log_10_ *P* values of each variant interacted with (**Panel A**) self-reported depression and PHQ score; (**Panel B**) self-reported anxiety and GAD score. Each point represents a single test of association between the G × E interactions (SNP × socioeconomic factors) and mental disorders ordered by genomic position. The third circos shows chromosome density. Red plots represent *P* < 1.25 × 10^−8^ and dark gray plots present *P* < 5 × 10^−8^. PHQ score – patient health questionnaire score, GAD score – generalised anxiety disorder scale score

In discovery cohort, GWEIS detected 63 significant loci for PHQ and could be mapped to 35 genes (**Figure 2**, panel A), such as rs141360714 (*ZNF622*, *P* = 1.69 × 10^−12^), rs11614439 (*CD69*, *P* = 2.11 × 10^−12^) and rs111606492 (*LILRB1*, *P* = 5.46 × 10^−10^). For GAD score, we identified 18 loci that were subsequently mapped to seven genes (**Figure 2****, panel B**), such as rs146434301 (*CHST9*, *P* = 2.52 × 10^−10^), rs140314791(*TMPRSS11D*, *P* = 1.06 × 10^−9^), and rs111606492 (*LILRB1*, *P* = 1.24 × 10^−9^). We did not detect significant loci for self-reported depression and anxiety, however, we identified several SNPs with *P* < 1 × 10^−5^. In replication cohort, all significant SNPs with *P* < 1.25 × 10^−8^ in discovery cohort showed nominal significance for both PHQ score and GAD score in the replication (*P* < 0.05). For self-reported depression and anxiety, several loci with *P* < 1 × 10^−5^ in discovery cohort were detected to be nominally significant in the replication. The detailed results for GWEIS are shown in Table S5-S9 in the **Online Supplementary Document**.

### Functional analysis results

The candidate genes detected by GWEIS were further annotated by FUMA. For PHQ score, we found the candidate gene sets were involved in GO: cerebral cortex tangential migration (*P* = 2.06 × 10^−5^), GO: tangential migration from the subventricular zone to the olfactory bulb (*P* = 3.83 × 10^−5^) and REACTOM: regulation of commissural axon pathfinding by slit and robo (*P* = 6.15 × 10^−5^). The detailed results are shown in Table S10 in the **Online Supplementary Document**.

### Multi-omics-based validation results

We queried candidate genes for PHQ score and GAD score in multi-omics databases and found multiple related diseases or traits at the genome, transcriptome and proteome levels, such as mental disorders, cognition, brain development and neurological function (Table S11 in the **Online Supplementary Document**). For instance, *GBE1* and *PIP4K2A* were associated with sensitivity to environmental stress and adversity at the genome level; *CTBP2P3* was associated with depression severity × hours spent watching television interaction at the genome level; *LCORL* was related to general cognitive ability at the genome level and intelligence at the transcriptome level; and *EPB41L1* was associated with intellectual developmental disorder at the protein level.

## DISCUSSION

Mental disorders in populations are strongly socially determined [31]. To date, the extent to which the combination of multidimensional social risk factors contributes to depression and anxiety remains unknown. We generated PsRS based on large-scale data from the UK Biobank cohort, and explored the link between PsRS with depression and anxiety. We found PsRS was significantly associated with higher PHQ and GAD scores, and the risk of self-reported anxiety and depression. We also conducted GWEIS analysis, and observed that PHQ score and GAD score were affected by multidimensional social risk and gene interactions.

In this study, PsRS was generated based on social factors from socioeconomic status, neighborhood and living environment, and psychological adversity domains. For individuals, various social risk conditions may co-exist and collectively contribute to adverse psychological and physical outcomes. As a integrated social risk indicator, PsRS is helpful to accurately and comprehensively measure social disadvantage by considering multiple social factors from various domains [10]. Recently, Zhao et.al used PsRS to estimate the participant-level total social risk exposure, and identified a positive association between PsRS and type 2 diabetes [11]. For mental disorders, previous studies always focused on the effects of single or several social factors, ignoring the coexistence of multidimensional social factors. Thus, the application of PsRS in the present study will more accurately to assess the real consequences of social vulnerabilities on mental issues. We found PsRS accounted for approximately 30% increased risk of depression, and about 20% increased risk of anxiety, showing the impairment of social disadvantage on mental disorders is not negligible. Our findings provide novel insight into the link between integrated social factors with depression and anxiety.

We used multivariable regression model to screen risk factors for mental disorders from the domains of socioeconomic status, psychological adversity, as well as neighborhood and living environment. Notably, lack of social support, emotional distress and low household income were the leading risk factors for depression and anxiety. Lack of social support from coworkers and family was associated with depressive symptoms in Japanese working population [32]. Spouse and family support can reduce the risk of depression in all age groups, and social support from spouse was related to the reduction of depression in the elderly group. Social support also had a significant moderating effect on the relationship between loneliness and depression in the old populations [33]. Emotional distress is a known risk factor for mental disorders. For instance, bereavement can have a long-term impact on the mental health of the surviving individuals [34], the presence of life stress will results in high possibility of psychological distress [35]. Low SES is well documented to be associated with higher rates of psychiatric illness, more disability, and less access to health care [36]. Low household income predicted higher rates of adverse psychological events [37,38].

Social exclusion may lead to reduced motivation, anticipatory anhedonia, and development of negative beliefs, which were the driving factors for psychotic disorders [39]. Undesirable psychosocial factors may challenge prior cognisation and trust in social interactions, promote attention to environmental stimuli, and trigger a cascade of changes in information processing [39]. Individuals living in lower socioeconomic neighborhoods and with less education may be exposed to more stress, thereby increasing susceptibility to mental illness [40,41]. Moreover, accessibility to mental health resources and utilisation of psychotherapy services tend to be lower among socially vulnerable groups [42]. The gross inequalities in health present a challenge to the current world. A burgeoning volume of studies showed social factors as the root cause of much of these inequalities in health, and social determinants are relevant to both communicable and non-communicable diseases, such as mental illness [43]. At present, many countries have formulated strategies and made efforts in creating social conditions and reducing health inequalities, and achieved encouraging results [44]. The strategies that mitigate the effect of social issues on mental disorders should be urgently considered when in treatment, nursing and social work.

In this study, GWEIS was conducted to preliminary explore interaction between gene and social risk factors for depression and anxiety, and try to identify potential loci. GWEIS is designed on the notion that individuals respond differently to environmental stimuli based on their genotype [45]. Traditional genome-wide association study (GWAS) focus on main effects and may not detect SNPs associated with diseases that are influenced by specific environmental factors. GWEIS can improve the identification of relevant genes by facilitating the integration of environmental information into GWAS [46]. Moreover, it can provide insights into significant contributors to etiological heterogeneity [47]. GWEIS was also considered as a suitable approach to gain relevant biological information from the gene × environment interaction analyses [47]. Currently, GWEIS has gained significant traction in the field of psychiatric disorders [48], such as depression [16-18], neuroticism [47] and anxiety [26].

We detected significant interactions between genetic loci and PsRS for depression and anxiety, and identified multiple candidate genes, such as *ST18*, *MYO9A* and *PIP4K2A*. *ST18* is a transcription factor that regulates neuronal differentiation [49]. A GWAS for Alzheimer disease found that *ST18* was associated with cortical atrophy [50]. *MYO9A* may play a pivotal role in controlling the molecular structure and function of hippocampal synapses [51]. *MYO9A* haploinsufficiency was reported to impair hippocampal synaptic transmission, spatial learning and memory [51]. *PIP4K2A* was reported to play a role in biological pathways that may be impaired in schizophrenia [52]. These findings provide preliminary insights into gene × social risk factor interactions involved in depression and anxiety. More in-depth follow-up studies using experimental or more controlled designs are required to further identify the modulation of specific gene expression by environmental factors.

The study is the first to assess the contribution of comprehensive social risk to depression and anxiety considering multiple dimensions, and explored the influence of the interactions between genetic factors and comprehensive social risk score on the mental phenotypes. Our findings highlight that comprehensive adverse social situation significantly increases the risk of developing depression and anxiety, and indicate that social risk factors may act as epigenetic drivers of mental disorders. We provide new insights into depression and anxiety interventions.

Some limitations should also be noted. First, this study was a cross-sectional analysis, and it is not yet possible to confirm the causal effect of PsRS on mental phenotypes. The field of gene-environment interaction research is actually quite complex, and our cross-sectional analysis has limitations in providing how the environment affects specific genes in the pathogenesis of depression and anxiety. The findings of GWEIS require further research, including experimental studies or more controlled study designs, to elucidate the regulation and mechanisms of specific gene expression by social risk factors. Second, the variance of genetic factors may be miscalculated in the genetic association models due to missing heritability, resulting in potential bias for the results. The heritability of depression and anxiety, as estimated from twin studies, typically falls in the range of 30-40% [53,54]. It has been well-documented that SNP-based heritability estimates tend to be substantially lower than those obtained from twin studies [54,55]. In our study, the heritability was estimated to be 13.35% for self-reported depression, 6.87% for self-reported anxiety, 6.96% for PHQ score, and 5.86% for GAD score, which was comparable with the heritability estimated in previous GWAS [54,55]. Missing heritability problem is a complex and longstanding challenge in the field of genetics. Several factors contribute to this challenge, such as the presence of rare variants and structural variants, phenotyping methodology, sample size and ethnicity diversity of the study subjects, as well as the inflation of twin-based heritability estimates because of shared environmental effects and gene-environment interplay [56-58]. Notably, due to the presence of gene-environment interaction effects, a substantial proportion of the shared environmental influences being wrongly attributed to genetic factors, causing an inflation of the heritability estimate in twin studies, and the actual heritability estimates are likely to be between SNP heritability and twin study heritability estimates [59,60]. In addition, it is reported that GWEIS are able to complement heritability estimates obtained from purely additive models [16]. If some of the unexplained heritability in GWAS is due to gene-environment interactions, it may be feasible to exploit interactions to discover novel variants that act synergistically with other factors without having demonstrable marginal effects [61,62]. In the present study, we focused exclusively on common variants and did not encompass rare or structural variants, and our study sample consisted solely of European individuals, which could contribute to missing heritability. We will focus on developing enhanced methods for capturing a broader range of genetic variations and collecting large scale multi-ethnic cohort data in our future research endeavors when investigating gene-environment interactions. Furthermore, using an independent cohort to replicate our findings will add a lot toward the validity. However, we do not currently possess another independent sample for replication analysis. Therefore, we endeavored to replicate the results by dividing study samples into discovery and replication cohorts [18,63,64]. We will collect novel study samples to confirm our findings in future research. Due to data limitations, we are unable to identify the biological responses to environmental exposures and their dynamic variations, which may result in unknown biases. We are committed to conduct a more detailed and in-depth research of environment triggers by prospective study and experimental verification in the future. Finally, to enhance consistency in constructing PsRS and minimise disparities among variables, we categorised these variables into binary form. This simplification may lead to a loss of some of the precision found in the original information. In future research, we will explore a more refined assessment strategy for measuring these social factors while carefully considering the trade-off between variables.

## CONCLUSIONS

In summary, we constructed the PsRS for depression and anxiety of UK Biobank participants. We found the aggregate of multidimensional social disadvantages was associated with the risk of depression and anxiety, and also detected genetic clues interacting with PsRS. Our findings suggest the significance of addressing social disadvantage in mental health promotion.

## Additional material


Online Supplementary Document
