# Supplementary material for: A multidimensional social risk atlas of depression and anxiety: An observational and genome-wide environmental interaction study
Source: J Glob Health. 2023 Dec 8;13:04146. doi: 10.7189/jogh.13.04146 (PMC10704948; doi:10.7189/jogh.13.04146)
Supplement: Online Supplementary Document [file jogh-13-04146-s001.pdf]

Supplementary Material

Supplementary definition in the UK Biobank

Definition of Patient Health Questionnaire (PHQ-9)

PHQ-9[1] is a classification algorithm with a total score (0-27) used to screen for and measure depression severity, focusing on nine depressive symptoms and signs (as detailed below： Little interest or pleasure in doing things (UKB: 20514), Feeling down, depressed, or hopeless (UKB:20510, Trouble sleeping(UKB: 20517), Feeling tired (UKB: 20519), Poor appetite or overeating (UKB: 20511), Feeling bad about yourself (UKB: 20507), Trouble concentrating (UKB: 20508), Moving or speaking slowly or fidgety or restless (UKB: 20518), Thoughts that you would be better off dead (UKB: 20513)). In order to meet the 0-3 score for each item of PHQ, the 9 symptom scores (1-4) of our team UK were all reduced by 1 point, which was then added up.

Definition of 7-item Generalized Anxiety Disorder Scale (GAD-7)

GAD-7[1] is a classification algorithm with a total score scale from 0-21 used to screen for and measure anxiety severity, focusing on seven anxious symptoms and signs (as detailed below: Feeling nervous, anxious or on edge (UKB: 20506), Not being able to stop or control worrying (UKB: 20509), Worrying too much about different things (UKB: 20520), Trouble relaxing (UKB: 20515), Being so restless that it is hard to sit still (UKB: 20516), Becoming easily annoyed or irritable (UKB: 20505), Feeling afraid as if something awful might happen (UKB: 20512)). In order to meet the 0-3 score for each item of GAD, the seven symptom scores (1-4) of UKB were all reduced by 1 point, and then were added up to generate GAD-7.

Definition of self-reported depression and anxiety

The criteria for the definition of depression and anxiety were according to the definitions of Davis et al[2]. The case group criteria of depression and anxiety were defined based on self-reports and came from three UK Biobank fields: 20002, 20126 and 20544. The case group of depression was selected based on the code 1286 (UKB: 20002), code 3,4 or 5 (UKB: 20126) and code 11 (UKB: 20544). The case group of anxiety was selected based on the code 1287 (UKB: 20002) and code 15 (UKB: 20544).

The participants who didn’t report depressive symptoms and screened negative on PHQ-9

or composite international diagnostic interview short-form (CIDI-SF)[3] were selected as the control group of the self-reported depression, and individuals without self-reported anxiety symptoms didn't screen positive on GAD-7 were defined as control group of self-reported anxiety. The details of the mental disorder definition are shown in supplementary materials.

Definition of single social factors

The definition of a single social factor refers to a recent study published by Zhao et.al [4]. For mental disorders, we selected 13 social factors from three domains (socioeconomic status[SES], neighborhood and living environment, psychosocial factors) for preliminary analysis[5-8]. Briefly, SES include 4 indicators: low household income, low education attainment, poor education quality, and not in paid employment. Social psychological factors include living alone, lack of social support, social inactivity, social isolation and emotional distress. Neighborhood and living environment domain consists of area-level material deprivation, high local crime rate, poor housing quality and instable accommodation. The definition of a single social factor refers to a recent study published by Zhao et.al [4]. All indicators were derived from questionnaire interviews with participants and were defined as binary phenotypes. The responses representing higher social risk were scored as 1, and answers on behalf of lower social risk were scored as 0. The details of the definition are as follows:

Social factors	Original data in UKB		Definition in the present study
	ID(UKB field)	Definition	
**Socioeconomic status**			
Low household income	Average total household income before tax (738)	1=Less than 18,000; 2=18,000 to 30,999; 3=31,000 to 51,999; 4=52,000 to 100,000; 5=Greater than 100,000	0 = average total household income before tax is more than £31,000; 1 = less than £31,000
Low education attainment	Qualifications (6138)	1=College or University degree; 2=A levels/AS levels or equivalent; 3=O levels/GCSEs or equivalent; 4=CSEs or equivalent; 5=NVQ or HND or HNC or equivalent; 6=Other professional qualifications eg: nursing, teaching	0 = highest education level is college or above; 1 = lower than college
Poor education quality	Education score-England (26414)<br>Education score-Scotland (26431)<br>Education score-Wales (26421)	1=In paid employment or self-employed; 2=Retired; 3=Looking after home and/or family; 4=Unable to work because of sickness or disability; 5=Unemployed; 6=Doing unpaid or voluntary work; 7=Full or part-time student	0 = below the median of education score; 0 = above the median
Not in paid employment	Current employment status (6142)	1=In paid employment or self-employed; 2=Retired; 3=Looking after home and/or family; 4=Unable to work because of sickness or disability; 5=Unemployed; 6=Doing unpaid or voluntary work; 7=Full or part-time student	0 = In paid employment or self-employed; 1 = others
**Psychosocial factors**			
Living alone	Number in household(709)	Continuous variable	0 = no; 1= yes
Lack of social support	Able to confide (2110)	5=Almost daily; 4=2-4 times a week; 3=About once a week;<br>2=About once a month; 1=Once every few months; 0=Never or almost never	0 = able to confide in anyone close to you at least once a week; 1 = once a week or less often
Social inactivity	Leisure/social activities (6160)	1=Sports club or gym; 2=Pub or social club; 3=Religious group; 4=Adult education class; 5=Other group activity	0 = attend any group activities once a week or more often; 1 = less often than once a week

Social isolation	Frequency of friend/family visits (1031)	1=Almost daily; 2=2-4 times a week; 3=About once a week; 4=About once a month; 5=Once every few months; 6=Never or almost never; 7=No friends/family outside household	0 = visit friend/family or have them visit you more often than once a week; 1 = once a week or less
Emotional distress	Illness, injury, bereavement, stress in last 2 years (6145)	1=Serious illness, injury or assault to yourself; 2=Serious illness, injury or assault of a close relative; 3=Death of a close relative; 4=Death of a spouse or partner; 5=Marital separation/divorce; 6=Financial difficulties	1 = have experienced illness, injury, bereavement, stress within last 2 years; 0 = none
**Neighborhood and living environment**			
Area-level material deprivation	Townsend deprivation index at recruitment (189)	Continuous variable	0 = below the median of Townsend deprivation index; 1 = above the median
High local crime rate	Crime score-England (26416)<br>Crime score-Scotland (26434)<br>Crime score-Wales(26425)	Continuous variable	0 = below the median of crime score; 1 = above the median
Poor housing quality	Housing score-England (26415)<br>Housing score-Scotland (26432)<br>Housing score-Wales (26423)	Continuous variable	0 = below the median of housing score; 1 = above the median
Instable accommodation	Own or rent accommodation lived in (680)	1=Own outright (by you or someone in your household); 2=Own with a mortgage; 3=Rent - from local authority, local council, housing association; 4=Rent - from private landlord or letting agency; 5=Pay part rent and part mortgage (shared ownership);<br>6=Live in accommodation rent free	0 = own current accommodation outright; 1 = own with mortgage

Reference

1 Kroenke K, Spitzer RL, Williams JB, Löwe B. The Patient Health Questionnaire Somatic, Anxiety, and Depressive Symptom Scales: a systematic review. Gen Hosp Psychiatry. 2010;32:345-59.

2 Davis KAS, Cullen B, Adams M, Brailean A, Breen G, Coleman JRI, et al. Indicators of mental disorders in UK Biobank-A comparison of approaches. Int J Methods Psychiatr Res. 2019;28:e1796.

3 Gigantesco A, Morosini P. Development, reliability and factor analysis of a self-administered questionnaire which originates from the World Health Organization's Composite International Diagnostic Interview - Short Form (CIDI-SF) for assessing mental disorders. Clin Pract Epidemiol Ment Health. 2008;4:8.

4 Zhao Y, Li Y, Zhuang Z, Song Z, Wang W, Huang N, et al. Associations of polysocial risk score, lifestyle and genetic factors with incident type 2 diabetes: a prospective cohort study. Diabetologia. 2022;65:2056-65.

5 Holmberg I, Persson G. Mental disorder at seventy in relation to social factors and attitudes during life. Acta Psychiatr Scand. 1986;74:168-77.

6 Otte C, Gold SM, Penninx BW, Pariante CM, Etkin A, Fava M, et al. Major depressive disorder. Nature Reviews Disease Primers. 2016;2:16065.

7 Qi X, Jia Y, Pan C, Li C, Wen Y, Hao J, et al. Index of multiple deprivation contributed to common psychiatric disorders: A systematic review and comprehensive analysis. Neurosci Biobehav Rev. 2022;140:104806.

8 Meltzer H, Bebbington P, Brugha T, Jenkins R, McManus S, Dennis MS. Personal debt and suicidal ideation. Psychol Med. 2011;41:771-8.

Supplementary figure

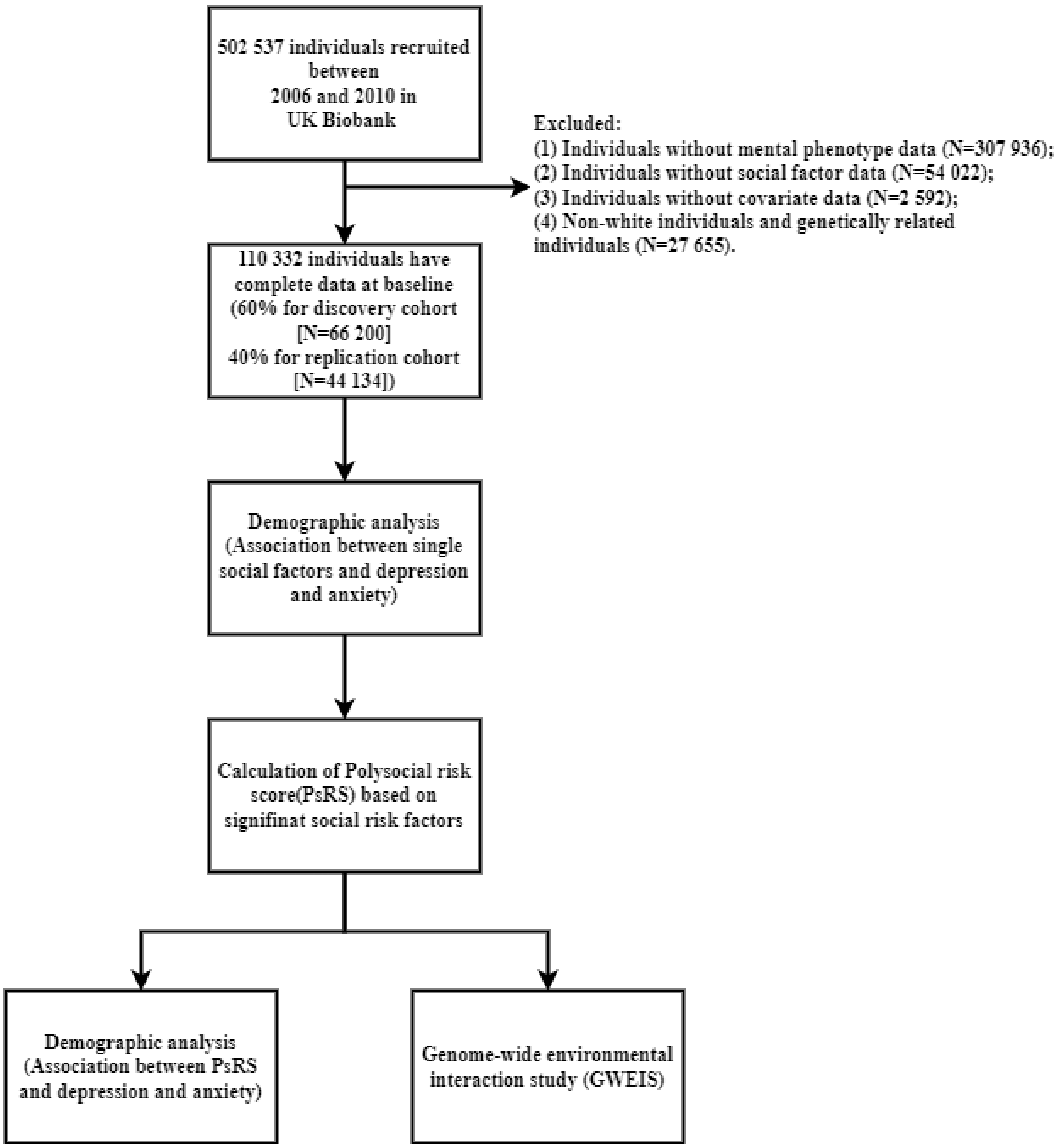

Figure S1. Flow chart of study design.

Supplementary tables

Table S1. Association between single social factor and depression and anxiety (original multivariable regression model).

Table S2. Association between single social risk factor and depression and anxiety (insignificant factors removed).

Table S3. Weights used to calculate PsRS based on multivariable models.

Table S4. The statistical summary of PsRS.

Table S5. The numbers of PsRS-interacted SNPs detected by GWEIS.

Table S6. The PsRS-interacted SNPs for PHQ score in discovery and replication cohort.

Table S7. The PsRS-interacted SNPs for GAD score in discovery and replication cohort.

Table S8. The PsRS-interacted SNPs for self-reported depression in discovery and replication cohort.

Table S9. The PsRS-interacted SNPs for self-reported anxiety in discovery and replication cohort.

Table S10. Functional analysis results for PHQ score candidate genes.

Table S11. Multi-omics-based validation for top candidate genes of mental phenotypes.

Table S1. Association between single social factor and depression and anxiety (original multivariable regression model).

Mental phenotype	Social factor	Discovery cohort			Replication cohort		
		Beta	SE	*P*	Beta	SE	*P*
PHQ score	Area-level material deprivation	0.151	0.032	2.31E-06	0.201	0.039	3.35E-07
	Instable accommodation	0.289	0.034	3.39E-17	0.230	0.042	4.11E-08
	Living alone	0.186	0.041	6.69E-06	0.131	0.050	8.97E-03
	Social isolation	0.063	0.030	0.038	0.046	0.037	0.216
	Low household income	0.379	0.035	4.60E-27	0.365	0.043	4.27E-17
	Lack of social support	0.839	0.034	1.27E-132	0.862	0.042	1.37E-93
	Low education attainment	0.159	0.030	8.44E-08	0.209	0.036	1.00E-08
	Not in paid employment	0.359	0.037	5.82E-22	0.339	0.046	1.47E-13
	Social inactivity	0.383	0.032	1.29E-32	0.437	0.040	2.41E-28
	Emotional distress	0.606	0.029	1.36E-95	0.626	0.036	1.98E-68
	High local crime rate	0.025	0.032	0.430	0.033	0.039	0.391
	Poor education quality	0.295	0.032	6.06E-20	0.222	0.039	1.82E-08
	Poor housing quality	0.021	0.029	0.470	0.036	0.035	0.310
GAD score	Area-level material deprivation	0.067	0.030	0.026	0.082	0.037	0.026
	Instable accommodation	0.202	0.032	3.22E-10	0.166	0.039	2.44E-05
	Living alone	-0.118	0.039	2.27E-03	-0.171	0.047	2.86E-04
	Social isolation	0.020	0.028	0.481	0.009	0.035	0.804
	Low household income	0.279	0.033	3.17E-17	0.267	0.041	5.07E-11
	Lack of social support	0.534	0.032	2.80E-62	0.579	0.039	3.65E-49
	Low education attainment	0.082	0.028	3.14E-03	0.119	0.034	5.07E-04
	Not in paid employment	0.213	0.035	1.11E-09	0.185	0.043	1.57E-05
	Social inactivity	0.256	0.030	2.42E-17	0.275	0.037	1.25E-13
	Emotional distress	0.454	0.027	8.31E-62	0.443	0.034	9.47E-40
	High local crime rate	0.029	0.030	0.321	0.026	0.036	0.466
	Poor education quality	0.125	0.030	3.57E-05	0.119	0.037	1.29E-03
	Poor housing quality	-0.008	0.027	0.779	0.021	0.033	0.531
Self-reported depression	Area-level material deprivation	0.151	0.021	1.79E-13	0.129	0.025	2.92E-07
	Instable accommodation	0.315	0.022	4.48E-46	0.299	0.027	1.53E-28
	Living alone	0.248	0.026	2.14E-21	0.202	0.032	2.27E-10
	Social isolation	-0.068	0.019	4.01E-04	-0.080	0.024	7.27E-04
	Low household income	0.485	0.022	1.26E-104	0.456	0.028	1.38E-61
	Lack of social support	0.267	0.022	2.32E-34	0.310	0.027	6.45E-31
	Low education attainment	0.084	0.019	1.35E-05	0.096	0.024	4.30E-05
	Not in paid employment	0.182	0.024	2.62E-14	0.145	0.029	6.75E-07
	Social inactivity	0.146	0.021	1.86E-12	0.205	0.025	7.66E-16
	Emotional distress	0.488	0.019	1.39E-151	0.496	0.023	9.99E-105
	High local crime rate	0.009	0.020	0.644	0.033	0.025	0.188
	Poor education quality	0.196	0.021	1.79E-21	0.173	0.025	6.28E-12
	Poor housing quality	-0.002	0.019	0.933	0.036	0.023	0.117
	Area-level material deprivation	0.043	0.027	0.115	0.016	0.033	0.637
	Instable accommodation	0.160	0.029	2.67E-08	0.160	0.035	6.17E-06
	Living alone	0.034	0.034	0.305	0.035	0.041	0.392

Self-reported anxiety	Social isolation	-0.040	0.025	0.110	-0.051	0.031	0.103
	Low household income	0.271	0.029	1.46E-20	0.254	0.036	1.43E-12
	Lack of social support	0.087	0.029	0.003	0.130	0.035	2.21E-04
	Low education attainment	-0.010	0.025	0.693	0.052	0.031	9.34E-02
	Not in paid employment	0.109	0.032	5.89E-04	0.139	0.039	3.37E-04
	Social inactivity	0.125	0.027	3.26E-06	0.158	0.033	1.56E-06
	Emotional distress	0.342	0.024	4.49E-45	0.308	0.030	5.35E-25
	High local crime rate	0.028	0.027	0.286	-0.001	0.033	0.975
	Poor education quality	0.109	0.027	5.11E-05	0.126	0.033	1.40E-04
	Poor housing quality	-0.107	0.024	1.23E-05	-0.018	0.030	0.553

Table S2. Association between single social risk factor and depression and anxiety (insignificant factors removed).

Mental phenotype	Social risk factor	Discovery cohort				Replication cohort			
		Beta/ OR	95% CI Lower	95% CI Upper	*P*	Beta/ OR	95% CI	95% CI Upper	*P*
PHQ score	Area-level material deprivation	0.16	0.10	0.22	1.18E-07	0.22	0.14	0.29	7.97E-09
	Instable accommodation	0.29	0.22	0.36	2.49E-17	0.23	0.15	0.31	3.78E-08
	Living alone	0.18	0.10	0.26	1.01E-05	0.13	0.03	0.23	9.83E-03
	Low household income	0.38	0.31	0.44	1.08E-26	0.36	0.28	0.45	8.09E-17
	Lack of social support	0.85	0.78	0.91	4.92E-136	0.87	0.78	0.95	1.92E-95
	Low education attainment	0.15	0.10	0.21	2.22E-07	0.20	0.13	0.27	2.23E-08
	Not in paid employment	0.35	0.28	0.42	2.44E-21	0.33	0.24	0.42	2.82E-13
	Social inactivity	0.39	0.32	0.45	1.89E-33	0.44	0.36	0.52	8.25E-29
	Emotional distress	0.60	0.55	0.66	5.12E-95	0.62	0.55	0.70	3.38E-68
	Poor education quality	0.30	0.24	0.36	2.75E-21	0.23	0.15	0.30	4.54E-09
GAD score	Instable accommodation	0.20	0.14	0.26	2.99E-10	0.17	0.09	0.24	2.59E-05
	Low household income	0.26	0.19	0.32	6.32E-16	0.23	0.16	0.31	2.78E-09
	Lack of social support	0.53	0.46	0.59	1.42E-61	0.56	0.49	0.64	1.01E-47
	Low education attainment	0.08	0.03	0.14	3.08E-03	0.12	0.05	0.18	4.57E-04
	Not in paid employment	0.21	0.15	0.28	5.86E-10	0.19	0.11	0.28	6.86E-06
	Social inactivity	0.26	0.20	0.32	7.28E-18	0.28	0.21	0.35	3.80E-14
	Emotional distress	0.45	0.40	0.51	8.40E-62	0.44	0.38	0.51	7.24E-40
	Poor education quality	0.15	0.09	0.20	2.01E-07	0.14	0.07	0.21	4.00E-05
Self-reported depression	Area-level material deprivation	1.17	1.12	1.21	5.25E-15	1.15	1.1	1.21	2.58E-09
	Instable accommodation	1.37	1.31	1.43	6.73E-46	1.35	1.28	1.42	1.66E-28
	Living alone	1.29	1.23	1.36	1.69E-22	1.24	1.16	1.31	2.82E-11
	Low household income	1.63	1.56	1.7	2.58E-106	1.58	1.5	1.67	4.34E-62
	Lack of social support	1.3	1.24	1.35	8.01E-33	1.35	1.28	1.42	1.83E-29
	Low education attainment	1.09	1.05	1.13	4.51E-06	1.1	1.05	1.16	2.27E-05
	Not in paid employment	1.21	1.15	1.27	1.71E-15	1.17	1.1	1.23	1.35E-07
	Social inactivity	1.15	1.11	1.2	7.94E-12	1.22	1.16	1.28	4.12E-15
	Emotional distress	1.63	1.58	1.69	2.15E-153	1.65	1.57	1.72	8.29E-106
	Poor education quality	1.22	1.17	1.27	2.16E-23	1.2	1.14	1.26	2.52E-13
	Instable accommodation	1.18	1.11	1.24	1.70E-08	1.18	1.1	1.26	3.97E-06
	Low household income	1.34	1.27	1.41	1.08E-25	1.32	1.24	1.41	2.64E-16

Self-reported anxiety	Lack of social support	1.09	1.03	1.15	3.18E-03	1.14	1.06	1.22	1.96E-04
	Not in paid employment	1.11	1.05	1.18	6.75E-04	1.15	1.07	1.24	2.13E-04
	Social inactivity	1.13	1.07	1.19	7.00E-06	1.17	1.1	1.25	1.77E-06
	Emotional distress	1.41	1.34	1.48	1.18E-45	1.36	1.29	1.45	1.95E-25
	Poor education quality	1.14	1.09	1.2	1.36E-07	1.15	1.08	1.22	5.21E-06

Table S3. Weights used to calculate PsRS based on multivariable models.

Social risk factor	PHQ score		GAD score		Self-reported		Self-reported	
	**Discovery**	**Replication**	**Discovery**	**Replication**	**Discovery**	**Replication**	**Discovery**	**Replication**
Area-level material deprivation	0.162	0.216	0.202	0.165	0.143	0.153	/	/
Instable accommodation	0.290	0.230	/	/	0.299	0.314	0.162	0.163
Living alone	0.181	0.129	/	/	0.211	0.254	/	/
Low household income	0.375	0.361	0.257	0.233	0.457	0.488	0.289	0.278
Lack of social support	0.846	0.866	0.525	0.563	0.300	0.260	0.084	0.130
Low education attainment	0.153	0.203	0.082	0.118	0.099	0.088	/	/
Not in paid employment	0.352	0.334	0.215	0.192	0.153	0.190	0.107	0.142
Social inactivity	0.388	0.440	0.260	0.280	0.199	0.141	0.120	0.157
Emotional distress	0.603	0.625	0.454	0.443	0.498	0.491	0.343	0.310
Poor education quality	0.298	0.226	0.148	0.143	0.180	0.200	0.132	0.140

Table S4. The statistical summary of PsRS.

	Mental Phenotype	Minimum	Quartile 1	Median	Quartile 3	Maximum
Discovery cohort	Self-reported anxiety	0	1.497	2.388	3.253	7.005
	Area-level material deprivation	0	1.613	2.583	3.722	8.000
	Self-reported depression	0	2.105	3.463	4.831	10.000
	PHQ score	0	2.003	3.119	4.461	9.997
Replication cohort	Self-reported anxiety	0	1.497	2.388	3.253	7.005
	GAD score	0	1.590	2.567	3.689	7.996
	Self-reported depression	0	2.170	3.462	4.801	10.000
	PHQ score	0	1.915	3.096	4.493	10.000

Table S5. The numbers of PsRS-interacted SNPs detected by GWEIS.

Mental Phenotype	Discovery cohort		N (*P* <1.25e-8 in discovery cohort and *P* <0.05 in replication cohort)
	N (*P* <5e-8)	N (*P* <1.25e-8)	
PHQ score	102	62	62
GAD score	30	18	18
Self-reported depression	0	0	0
Self-reported anxiety	1	0	0
Total	133	80	80

Note. N, the number of PsRS-interacted SNPs.

Table S6. The PsRS-interacted SNPs for PHQ score in discovery and replication cohort (Marked in red is significant).

SNP	CHR	Position	Eeference allele	Alternative allele	*P* discovery	*P* replication	Overlapped gene
rs141360714	5	16239143	G	T	1.69E-12	0.000659135	*ZNF622*
rs147165657	5	16274471	G	A	2.10E-12	0.0123691	*ZNF622*
rs11614439	12	9902857	T	C	2.11E-12	1.41E-08	*CD69*
rs2689542	7	152542459	A	G	4.69E-11	0.0007633	*ACTR3B*
rs542946033	7	152543389	C	T	8.34E-11	0.00246084	*ACTR3B*
rs4591276	19	55029367	C	G	1.35E-10	0.000107614	*AC008746.9*
rs144469813	4	20274271	T	C	1.75E-10	0.000606422	*SLIT2*
rs117345247	20	34773553	A	G	3.79E-10	5.85E-06	*EPB41L1*
rs10518045	4	68710842	C	T	4.50E-10	7.33E-06	*TMPRSS11D*
rs2860008	4	68710963	C	T	4.51E-10	7.34E-06	*TMPRSS11D*
rs116587563	4	68710465	A	C	4.51E-10	7.33E-06	*TMPRSS11D*
rs140314791	4	68709501	T	C	4.53E-10	7.31E-06	*TMPRSS11D*
rs17088693	4	68709690	C	T	4.53E-10	7.31E-06	*TMPRSS11D*
rs114006170	4	68704816	T	C	4.81E-10	4.55E-06	*TMPRSS11D*
rs116531749	3	82128014	C	T	5.12E-10	0.00114896	*GBE1*
8:41453210_CTCTT_C	8	41453210	C	CTCTT	5.20E-10	0.00279471	/
rs111606492	19	55105969	A	C	5.46E-10	2.68E-10	*LILRB1*
rs148060334	4	68705712	G	A	6.33E-10	7.60E-06	*TMPRSS11D*
rs1545935	4	68700984	C	G	6.34E-10	6.84E-06	*TMPRSS11D*
rs17576689	4	68704958	C	T	6.35E-10	7.57E-06	*TMPRSS11D*
rs115427586	4	68681137	C	T	6.35E-10	9.44E-06	*TMPRSS11D*
rs76632488	4	68684456	A	G	6.42E-10	9.75E-06	*TMPRSS11D*
rs150629496	3	82006561	C	G	7.27E-10	0.000285426	*GBE1*
rs117969745	18	64889618	C	A	9.31E-10	5.54E-06	*RPL31P9*
rs182485596	6	76667042	T	C	1.32E-09	0.000100584	*IMPG1*
rs147509787	7	46287804	G	T	1.33E-09	0.0036796	*HMGN1P19*
4:68667012_CAAT_C	4	68667012	C	CAAT	1.64E-09	4.48E-05	/
rs112030069	1	213919006	C	G	2.12E-09	0.0141236	*PROX1*
rs112672202	1	213917791	T	C	2.31E-09	0.0139595	*PROX1*
rs28529767	1	206300551	T	C	2.33E-09	0.0209615	*CTSE*
rs183848315	7	152425977	A	G	2.64E-09	0.00429495	*XRCC2*
rs142221374	3	153568239	G	A	2.93E-09	0.00531383	*C3orf79*
rs17603507	15	30234960	G	A	3.29E-09	0.000350721	*TJP1*
rs116107595	3	81624468	C	T	3.34E-09	0.000718239	*GBE1*
rs118133461	7	152420994	A	G	3.39E-09	0.00362656	*XRCC2*
rs76722576	7	152421261	A	C	3.86E-09	0.0036653	*XRCC2*
rs3759812	15	72100019	G	C	3.87E-09	6.21E-05	*MYO9A*
rs6863037	5	119686074	A	G	4.22E-09	0.000651188	*PRR16*
rs144501423	5	119670176	G	A	4.53E-09	0.000646586	*PRR16*
rs144464609	4	108161365	G	T	5.16E-09	0.000152513	*DKK2*
rs142478061	1	207483392	A	G	5.26E-09	0.0490296	*CD55*
rs149533016	4	18319183	A	G	5.27E-09	1.43E-06	*LCORL*
rs142268508	3	81846513	T	C	5.42E-09	0.000118662	*GBE1*
rs113644415	18	64892371	T	C	5.67E-09	1.42E-05	*RPL31P9*
rs11661452	18	64893826	T	C	5.79E-09	1.44E-05	*RPL31P9*
rs79824688	5	141506354	T	C	6.96E-09	0.00419647	*NDFIP1*
rs148275421	3	194661899	T	G	8.34E-09	0.0056306	*XXYLT1*
rs77140099	1	181391031	A	G	8.84E-09	0.0181991	*CACNA1E*
rs74594895	16	10839907	G	A	9.45E-09	0.0307412	*NUBP1*
rs75482345	20	61724045	T	C	9.55E-09	0.0004998	*BHLHE23*
rs115357900	3	81719118	T	A	9.63E-09	0.000396712	*GBE1*
rs80029890	7	54449520	T	G	9.98E-09	0.000340082	*SLC25A5P3*

rs146348447	15	100798747	C	T	1.06E-08	0.0234018	*ADAMTS17*
rs117918935	15	100798756	A	G	1.08E-08	0.0203625	*ADAMTS17*
rs117676280	18	58355538	C	T	1.08E-08	1.68E-06	*CTBP2P3*
rs149137169	8	53343488	A	C	1.08E-08	3.25E-06	*ST18*
rs62849163	3	58558781	T	A	1.09E-08	0.000577234	*FAM107A*
rs147753363	4	18381341	C	T	1.13E-08	9.98E-08	*LCORL*
rs115300689	3	81736535	A	C	1.15E-08	0.000290053	*GBE1*
rs149469089	3	68327787	G	C	1.21E-08	4.31E-08	*FAM19A1*
rs370273048	18	62216618	T	G	1.21E-08	0.0313918	*CDH7*
rs116332061	3	79825415	C	G	1.21E-08	0.00032627	*ROBO1*
rs112952213	4	18315475	C	G	1.26E-08	1.03E-07	*LCORL*
rs149965693	3	128248170	T	C	1.48E-08	0.0244584	*GATA2*
rs117175198	17	7012342	T	C	1.49E-08	9.33E-08	*ASGR2*
rs72989664	18	76826364	A	T	1.55E-08	0.0105614	*ATP9B*
rs187269353	2	13670135	C	T	1.69E-08	0.0236091	*TRIB2*
rs547141653	15	30237979	A	AACACAC ACAC	1.74E-08	0.000348346	/
rs9829418	3	77424153	G	A	1.86E-08	2.94E-05	*ROBO2*
rs72638373	13	95330457	T	G	1.89E-08	0.00427868	*SOX21*
rs2086051	4	18478543	A	G	2.06E-08	1.73E-07	*LCORL*
rs73094392	4	18466752	G	C	2.06E-08	1.96E-07	*LCORL*
rs148161662	3	81711532	G	A	2.07E-08	0.000264396	*GBE1*
rs72638377	13	95331826	A	C	2.13E-08	0.00387939	*SOX21*
rs184710548	7	152535893	G	A	2.21E-08	0.0351477	*ACTR3B*
rs116638258	3	81869054	A	G	2.26E-08	0.000174517	*GBE1*
rs78856971	4	68672846	G	T	2.33E-08	8.77E-06	*TMPRSS11D*
rs41525951	4	68669704	C	T	2.34E-08	8.61E-06	*TMPRSS11D*
rs114698375	2	106054656	C	G	2.71E-08	0.000602855	*FHL2*
rs12403496	1	48484051	T	C	2.95E-08	0.00764491	*CYP46A4P*
rs560272307	18	49267154	A	G	2.99E-08	0.000844276	*RPS8P3*
rs115594478	3	79701623	A	G	3.04E-08	0.000856885	*ROBO1*
rs73136325	3	98196626	A	T	3.06E-08	0.0272117	*OR5K2*
rs116782885	3	82258990	C	T	3.30E-08	0.00495869	*GBE1*
rs79163829	2	11538351	A	G	3.34E-08	1.36E-06	*E2F6*
rs74631028	16	12789955	C	T	3.37E-08	0.036485	*CPPED1*
rs192415402	1	214563395	T	C	3.54E-08	2.10E-07	*PTPN14*
rs139424815	10	3109286	G	C	3.55E-08	0.000286175	*PFKP*
rs117793374	18	49313675	C	T	3.62E-08	0.000106258	*RPS8P3*
rs77814262	3	81865372	G	A	3.62E-08	0.000392649	*GBE1*
rs11638904	15	37309303	G	C	3.81E-08	0.000109191	*MEIS2*
rs146398630	11	67997388	T	C	4.08E-08	0.00014852	*SUV420H1*
rs138004333	6	69596677	C	T	4.13E-08	0.00265024	*ADGRB3*
rs35381071	4	68694828	T	TG	4.21E-08	0.000316889	*TMPRSS11D*
rs192290563	11	57619332	C	T	4.24E-08	1.17E-09	*CTNND1*
4:107007289_C A_C	4	107007289	C	CA	4.26E-08	0.00444221	/
rs143089210	8	11401503	A	G	4.27E-08	0.0215525	*BLK*
rs144173106	15	100793456	GCCTAC ACT	G	4.28E-08	0.0302281	*ADAMTS17*
rs4938877	11	57629180	A	G	4.39E-08	1.16E-09	*OR5BA1P*
rs12215234	6	49640046	G	A	4.45E-08	0.00721398	*RHAG*
rs694158	13	78132151	G	A	4.52E-08	1.22E-05	*SCEL*
rs117304140	9	102014351	C	A	4.52E-08	0.00163237	*SEC61B*
rs183917631	14	86791060	A	G	5.06E-08	2.30E-09	*FLRT2*
rs542897254	7	152534260	CA	C	5.23E-08	0.0350421	*ACTR3B*
rs36013500	2	152850229	T	C	5.63E-08	7.40E-05	*CACNB4*
rs71451165	11	29405492	T	C	5.66E-08	0.000854346	*RP11-460B17.1*
rs180916209	7	152536114	T	C	5.89E-08	0.0434681	*ACTR3B*

rs114250585	4	68667630	A	G	5.94E-08	1.83E-05	*TMPRSS11D*
rs115935519	3	19333451	T	C	6.43E-08	1.56E-05	*KCNH8*
rs79514712	3	81072180	G	C	6.82E-08	0.0126295	*RP11-481N16.1*
rs115709053	1	37596458	T	G	7.39E-08	0.000278912	*GRIK3*
rs74440514	15	100794511	A	G	7.63E-08	0.0415094	*ADAMTS17*
rs76619482	17	69067238	G	A	7.95E-08	0.000236923	*LOC124685*
rs114069551	4	71335556	G	A	8.00E-08	0.0104434	*MUC7*
rs78470634	4	162171584	T	C	8.79E-08	0.00106042	*FSTL5*
rs117827035	15	100792136	A	G	8.80E-08	0.0303534	*ADAMTS17*
rs74750249	6	5459680	A	C	8.89E-08	4.05E-06	*FARS2*
rs79749840	4	162169308	T	C	8.95E-08	0.00106522	*FSTL5*
rs72996103	18	76933689	A	G	8.95E-08	0.00546535	*ATP9B*
rs76884626	15	100795559	C	G	8.97E-08	0.030336	*ADAMTS17*
rs75760538	15	100793065	C	T	8.98E-08	0.0303519	*ADAMTS17*
rs74771048	15	100793128	G	T	8.99E-08	0.0303538	*ADAMTS17*
rs117053084	15	100794780	C	G	9.00E-08	0.0303164	*ADAMTS17*
rs117268382	15	100793356	C	A	9.00E-08	0.0302916	*ADAMTS17*
rs78312362	10	119061917	T	C	9.25E-08	6.57E-06	*PDZD8*
rs181745281	10	119072662	C	T	9.32E-08	6.64E-06	*PDZD8*
rs74393558	1	180154806	G	C	9.33E-08	0.00455335	*QSOX1*
rs113805345	2	199331728	G	T	9.38E-08	0.0311249	*PLCL1*
rs117669701	15	100796714	T	C	9.39E-08	0.0289186	*ADAMTS17*
rs76071867	17	10907886	T	G	9.63E-08	0.000257374	*SHISA6*
rs138580469	15	100787030	G	A	9.65E-08	0.040726	*ADAMTS17*
rs117098802	15	54221040	G	A	1.04E-07	7.94E-09	*UNC13C*
rs138332173	15	54215950	T	C	1.10E-07	7.83E-09	*UNC13C*
rs569519981	3	63749870	G	A	1.14E-07	0.000121297	*AC136289.1*
rs116138253	21	46675571	T	C	1.17E-07	0.001852	*POFUT2*
rs6832397	4	190200123	A	G	1.24E-07	0.000380541	*RP11-756P10.3*
rs140890408	14	36161825	A	G	1.28E-07	0.00133696	*RALGAPA1*
rs76063481	21	28728691	T	C	1.32E-07	1.09E-06	*GPX1P2*
rs114501114	3	80278910	A	G	1.33E-07	0.000956861	*HNRNPA3P8*
rs75057723	20	13156449	C	A	1.35E-07	9.63E-07	*ISM1*
rs4938874	11	57422341	A	G	1.39E-07	1.92E-09	*CLP1*
rs147601957	4	190180163	C	T	1.41E-07	0.000399386	*RP11-756P10.3*
rs138959917	4	190176612	G	A	1.43E-07	0.000402175	*RP11-756P10.3*
rs150427941	4	107253824	CTATAGA	C	1.43E-07	0.00538083	*AIMP1*
rs554441230	4	190174901	ATTATTCAAATATATTGAATAATATTAAAATTATTCAAAACTT	A	1.45E-07	0.000405469	*RP11-756P10.3*
rs17571316	15	30173956	T	C	1.46E-07	2.19E-05	*TJP1*
4:190205605_GA_G	4	190205605	G	GA	1.47E-07	0.000111851	/
rs7696747	4	107296413	C	A	1.51E-07	0.00198504	*AIMP1*
rs1971345	3	15400526	A	G	1.53E-07	0.048003	*SH3BP5*
rs79537527	4	189776630	A	G	1.55E-07	1.05E-05	*RP11-756P10.3*
rs138345778	4	107092786	GATA	G	1.57E-07	0.00473157	/
rs141135105	4	190228491	T	C	1.59E-07	0.000444298	*RP11-756P10.3*
rs150173241	4	190227592	A	G	1.59E-07	0.000428428	*RP11-756P10.3*
rs115329882	4	190230829	G	A	1.60E-07	0.000447357	*RP11-756P10.3*
rs10019578	4	190233217	A	G	1.60E-07	0.000459188	*RP11-756P10.3*
rs11097917	4	107096480	T	C	1.61E-07	0.00501066	*TBCK*

rs12511305	4	107098336	A	G	1.62E-07	0.00502501	*TBCK*
rs11097919	4	107099131	C	T	1.63E-07	0.00502919	*TBCK*
rs12499891	4	107097909	G	T	1.63E-07	0.00502661	*TBCK*
rs7661565	4	107097404	A	G	1.63E-07	0.00502664	*TBCK*
rs1186457	7	22027745	C	A	1.65E-07	0.00247622	*RAPGEF5*
rs12498715	4	107146625	T	G	1.69E-07	0.00428228	*TBCK*
rs7697673	4	107296620	T	G	1.70E-07	0.0016979	*AIMP1*
rs559768321	4	190174904	CAATTAA	C	1.74E-07	0.000406313	*RP11-756P10.3*
rs4747620	10	28732111	G	A	1.75E-07	7.04E-07	*WAC*
rs147733826	1	214921243	A	C	1.76E-07	0.00917917	*CENPF*
rs368131533	4	190192067	A	G	1.83E-07	0.000424429	*RP11-756P10.3*
rs373839230	4	190224627	A	G	1.85E-07	0.000289415	*RP11-756P10.3*
rs201980162	4	107137326	A	G	1.89E-07	0.00431588	*TBCK*
rs114991996	3	79729232	C	T	1.89E-07	0.0139792	*ROBO1*
rs34329524	4	179429967	A	G	1.94E-07	0.0176402	*RP11-84H6.1*
rs117029312	15	30264181	A	G	1.95E-07	0.000615874	*TJP1*
rs149126506	19	35911133	C	G	1.97E-07	0.000415994	*FFAR2*
rs72878537	4	107220049	G	A	1.98E-07	0.00440575	*TBCK*
rs6533245	4	107202935	T	C	2.02E-07	0.00459681	*TBCK*
rs7786820	7	139006963	A	G	2.06E-07	0.00914387	*UBN2*
rs7665946	4	107189328	C	T	2.07E-07	0.00446022	*TBCK*
rs3775090	4	107168520	T	G	2.08E-07	0.00446912	*TBCK*
rs17274248	4	107205662	T	C	2.08E-07	0.00450787	*TBCK*
rs17333732	4	107205009	T	C	2.08E-07	0.0045012	*TBCK*
rs578075416	2	11541938	C	A	2.08E-07	6.39E-08	*E2F6*
rs11097925	4	107161493	G	A	2.08E-07	0.00448273	*TBCK*
rs17333879	4	107220426	A	G	2.09E-07	0.00451395	*TBCK*
rs75055072	4	107201362	A	T	2.09E-07	0.00451168	*TBCK*
rs113142685	4	107210783	G	A	2.09E-07	0.0045155	*TBCK*
rs12500775	4	107213501	G	C	2.09E-07	0.0045155	*TBCK*
rs111450955	4	107215694	A	G	2.09E-07	0.0045155	*TBCK*
rs72876590	4	107204499	A	T	2.09E-07	0.00451742	*TBCK*
rs72876592	4	107204776	C	T	2.09E-07	0.00451742	*TBCK*
rs34899289	4	107194390	G	A	2.10E-07	0.00451098	*TBCK*
rs12510058	4	107188835	C	T	2.10E-07	0.0045043	*TBCK*
rs12645698	4	107188432	T	C	2.10E-07	0.0045043	*TBCK*
rs61544154	4	107189771	T	C	2.10E-07	0.0045043	*TBCK*
rs75814353	4	107200154	T	C	2.10E-07	0.00451072	*TBCK*
rs12639869	4	107176104	A	C	2.10E-07	0.00450895	*TBCK*
rs12642059	4	107173793	A	C	2.10E-07	0.00450895	*TBCK*
rs56333662	4	107177196	T	A	2.10E-07	0.00450895	*TBCK*
rs117699451	21	28716047	T	A	2.11E-07	1.28E-06	*GPX1P2*
rs79468403	4	107074786	C	T	2.11E-07	0.00420242	*TBCK*
rs80112814	4	107073081	C	A	2.11E-07	0.0042044	*TBCK*
rs151186466	4	107067567	G	A	2.11E-07	0.00420818	*TBCK*
rs7688411	4	107189665	T	C	2.11E-07	0.00495156	*TBCK*
rs12646263	4	107083746	T	C	2.11E-07	0.00420615	*TBCK*
rs143999404	4	107137621	A	C	2.11E-07	0.00432357	*TBCK*
rs190663640	4	107135466	A	G	2.11E-07	0.00432357	*TBCK*
rs4466113	4	107082043	C	T	2.11E-07	0.0042074	*TBCK*
rs12647078	4	107084333	A	G	2.11E-07	0.00420945	*TBCK*
rs17273093	4	107086739	C	G	2.11E-07	0.00421077	*TBCK*
rs78066595	4	107113954	GT	G	2.12E-07	0.00432564	*TBCK*
rs11097914	4	107070806	C	G	2.12E-07	0.00468405	*TBCK*
rs59784613	4	107073523	G	A	2.12E-07	0.00467338	*TBCK*
rs6816043	4	190217278	A	G	2.12E-07	0.000415785	*RP11-756P10.3*
rs6533246	4	107203084	T	C	2.13E-07	0.00500295	*TBCK*

rs72876594	4	107207527	T	C	2.13E-07	0.0049956	*TBCK*
rs7688008	4	107216896	C	G	2.13E-07	0.00501428	*TBCK*
rs56009469	4	107180715	G	C	2.13E-07	0.0049983	*TBCK*
rs7662291	4	107185593	A	G	2.13E-07	0.00499607	*TBCK*
rs7673949	4	107194036	A	G	2.13E-07	0.00499607	*TBCK*
rs7662395	4	107185855	T	C	2.13E-07	0.00498874	*TBCK*
rs7669212	4	107186712	A	G	2.13E-07	0.00498874	*TBCK*
4:107160985_TAA_T	4	107160985	T	TAA	2.13E-07	0.00446372	/
rs56231393	4	107180864	T	G	2.14E-07	0.00499383	*TBCK*
rs7662219	4	107185759	A	C	2.14E-07	0.00499945	*TBCK*
rs77545165	4	107123821	G	C	2.14E-07	0.00427461	*TBCK*
rs6818626	4	107123463	C	T	2.14E-07	0.00427835	*TBCK*
rs12641015	4	107172182	T	C	2.14E-07	0.00497862	*TBCK*
rs9685018	4	107124824	T	C	2.14E-07	0.00430275	*TBCK*
rs7667719	4	107186255	C	A	2.15E-07	0.0049974	*TBCK*
rs72893620	4	107187914	A	G	2.15E-07	0.00500434	*TBCK*
rs111765566	3	76098110	C	T	2.16E-07	5.97E-05	*ROBO2*
rs34193646	4	107151929	T	C	2.16E-07	0.00443563	*TBCK*
rs55634475	4	107067750	C	T	2.16E-07	0.00467777	*TBCK*
rs79051739	4	107150530	C	T	2.16E-07	0.00443874	*TBCK*
rs12512966	4	107082411	C	A	2.16E-07	0.00467773	*TBCK*
rs11097916	4	107087390	T	C	2.16E-07	0.00468093	*TBCK*
rs12512877	4	107145600	A	G	2.17E-07	0.00443996	*TBCK*
rs77722499	5	119834179	T	C	2.17E-07	0.0159772	*PRR16*
rs141144669	4	107086150	AATTT	A	2.17E-07	0.00442988	/
rs140333632	4	107127393	TG	T	2.18E-07	0.0045561	/
rs12641037	4	107142052	A	G	2.18E-07	0.004434	*TBCK*
rs202205774	4	107136248	C	T	2.18E-07	0.00480009	*TBCK*
rs12504704	4	107099563	G	A	2.19E-07	0.00482936	*TBCK*
rs56329826	4	107121031	C	T	2.19E-07	0.00475072	*TBCK*
rs12506062	4	107115960	G	A	2.19E-07	0.00475101	*TBCK*
rs12507429	4	107117686	C	A	2.19E-07	0.00475101	*TBCK*
rs17273445	4	107115665	A	C	2.19E-07	0.00475101	*TBCK*
rs6818197	4	107123210	C	T	2.19E-07	0.00478205	*TBCK*
rs535504686	6	151165899	GT	G	2.19E-07	1.03E-06	*PLEKHG1*
rs6822692	4	107108164	T	C	2.20E-07	0.00474076	*TBCK*
rs2290607	4	107113299	G	A	2.20E-07	0.00474905	*TBCK*
rs6533240	4	107112812	C	G	2.20E-07	0.00474929	*TBCK*
rs147343707	4	107110519	AAAAG	A	2.20E-07	0.00474569	*TBCK*
rs12503753	4	107068827	G	A	2.20E-07	0.0048254	*TBCK*
rs117909654	21	32192981	T	C	2.28E-07	0.00040508	*KRTAP8-1*
rs35317905	4	107199237	ATG	A	2.29E-07	0.00480659	*TBCK*
rs3840308	4	107254996	GCT	G	2.32E-07	0.00476529	*AIMP1*
rs769072295	4	107216072	C	CTA	2.32E-07	0.00429284	/
rs6831665	4	107222086	T	C	2.36E-07	0.00490052	*TBCK*
rs142748972	17	33616733	T	C	2.37E-07	0.00328119	*SLFN5*
4:107152379_GA_G	4	107152379	G	GA	2.37E-07	0.00466463	/
rs56833325	4	107222665	T	C	2.38E-07	0.00491812	*TBCK*
rs73002025	18	76988674	G	A	2.41E-07	0.039556	*ATP9B*
rs75511400	2	34919372	A	G	2.44E-07	0.000131167	*AC013442.1*
rs73154206	7	143086010	T	C	2.45E-07	1.42E-06	*ZYX*
rs11947335	4	107270658	A	G	2.47E-07	0.00505116	*AIMP1*
rs17456104	9	131948229	C	T	2.48E-07	3.32E-05	*IER5L*
rs34622492	8	137328025	G	C	2.54E-07	0.000144385	*KHDRBS3*
rs78708480	8	137329881	T	C	2.54E-07	0.00015342	*KHDRBS3*
rs143774502	15	77742598	A	G	2.55E-07	0.00707433	*HMG20A*

rs62380616	5	150175414	A	G	2.57E-07	7.36E-05	*SMIM3*
rs7696662	4	107243414	G	A	2.58E-07	0.00654967	*AIMP1*
rs11940160	4	107247367	G	A	2.59E-07	0.00596038	*AIMP1*
4:107246676_TA_T	4	107246676	T	TA	2.59E-07	0.00511889	/
rs12732933	1	76708130	A	T	2.60E-07	0.000184441	*ST6GALNAC3*
rs113059611	3	66724521	G	A	2.60E-07	7.70E-05	*KBTBD8*
rs201062605	4	107215353	CT	C	2.60E-07	0.0102616	*TBCK*
rs7674796	4	107243261	C	T	2.62E-07	0.00606716	*AIMP1*
rs147232944	4	107241983	C	T	2.62E-07	0.00665533	*AIMP1*
rs34144884	8	137322763	C	T	2.63E-07	0.00015305	*KHDRBS3*
rs35976733	8	137321672	T	C	2.64E-07	0.000153232	*KHDRBS3*
rs34229749	8	137321701	A	C	2.64E-07	0.000153243	*KHDRBS3*
rs71530153	8	137322752	A	G	2.64E-07	0.000153006	*KHDRBS3*
rs34329477	8	137318877	G	A	2.64E-07	0.000152007	*KHDRBS3*
rs76188442	20	7720259	G	A	2.64E-07	0.00229158	*BMP2*
rs10433894	4	107251400	G	C	2.66E-07	0.00665007	*AIMP1*
rs6819224	4	107250658	A	G	2.66E-07	0.00665016	*AIMP1*
rs35930320	8	137324423	C	T	2.66E-07	0.000153538	*KHDRBS3*
rs71530154	8	137325505	A	G	2.66E-07	0.000153483	*KHDRBS3*
rs34545445	8	137326674	T	C	2.66E-07	0.000153489	*KHDRBS3*
rs3737498	4	107246136	A	C	2.66E-07	0.00665476	*AIMP1*
rs35667165	8	137337508	T	C	2.68E-07	0.000158183	*KHDRBS3*
rs35471969	8	137338283	C	T	2.68E-07	0.000158588	*KHDRBS3*
rs13269261	8	137338230	A	C	2.68E-07	0.000158587	*KHDRBS3*
rs13261516	8	137346070	G	A	2.71E-07	0.00016745	*KHDRBS3*
rs34118496	8	137343203	G	T	2.71E-07	0.000167331	*KHDRBS3*
rs13259351	8	137344523	T	C	2.71E-07	0.000166853	*KHDRBS3*
rs114021007	5	155373160	T	C	2.71E-07	5.86E-05	*SGCD*
4:107241814_AT_A	4	107241814	A	AT	2.72E-07	0.00590029	/
rs6819742	4	107268016	C	A	2.74E-07	0.00617543	*AIMP1*
8:137347539_TTTTC_T	8	137347539	T	TTTTC	2.75E-07	0.000420776	/
rs76356450	8	137329763	T	C	2.77E-07	0.000153504	*KHDRBS3*
rs12642454	4	107266883	G	A	2.78E-07	0.00627394	*AIMP1*
rs547516699	7	152518557	GAA	G	2.78E-07	0.0128225	/
rs6828660	4	107269794	A	G	2.78E-07	0.00626689	*AIMP1*
rs3805410	4	107268949	G	A	2.78E-07	0.0062702	*AIMP1*
rs3805411	4	107269053	G	A	2.78E-07	0.00627042	*AIMP1*
rs3805409	4	107266401	G	C	2.78E-07	0.00628008	*AIMP1*
rs532856191	5	37589337	C	T	2.78E-07	0.000249594	*WDR70*
rs34933283	8	137317058	C	T	2.79E-07	0.000164639	*KHDRBS3*
rs6858391	4	107261654	A	G	2.79E-07	0.00636521	*AIMP1*
rs10516534	4	107256703	G	T	2.79E-07	0.00682913	*AIMP1*
rs970284	20	41280429	G	A	2.79E-07	0.00598651	*PTPRT*
rs11097928	4	107263868	T	A	2.80E-07	0.00629013	*AIMP1*
rs55753411	4	107264803	G	A	2.80E-07	0.00629143	*AIMP1*
rs11097927	4	107262793	C	G	2.80E-07	0.00629839	*AIMP1*
rs6831032	4	107257696	A	G	2.81E-07	0.00632044	*AIMP1*
rs55726133	4	107255868	A	G	2.82E-07	0.00633	*AIMP1*
rs113866132	7	132050334	C	T	2.83E-07	0.00031118	*PLXNA4*
4:107173543_TTAG_T	4	107173543	T	TTAG	2.84E-07	0.00388941	/
rs74509579	8	137316372	A	G	2.85E-07	0.000172328	*KHDRBS3*
rs35106727	8	137312815	C	T	2.90E-07	0.000176101	*KHDRBS3*
4:107253771_CAT_C	4	107253771	C	CAT	2.99E-07	0.00729124	/

rs113848226	3	66721559	T	G	3.06E-07	7.69E-05	*KBTBD8*
rs145237635	17	1333895	A	G	3.10E-07	0.00035789	*CRK*
rs148777093	20	55681131	T	C	3.12E-07	0.000120898	*BMP7*
rs11461633	4	107227770	TC	T	3.13E-07	0.00389626	*TBCK*
rs2425506	20	41285430	C	T	3.13E-07	0.00222869	*PTPRT*
rs144017138	4	107233622	TGAG	T	3.14E-07	0.00456107	*TBCK*
rs76506384	7	54312950	T	G	3.16E-07	0.00106517	*SLC25A5P3*
rs6856408	4	107236868	G	A	3.20E-07	0.00623843	*AIMP1*
rs3762947	4	107236648	G	A	3.20E-07	0.00623901	*TBCK*
rs117157297	18	49722182	G	A	3.23E-07	1.35E-06	*DCC*
rs6849341	4	107235750	G	A	3.29E-07	0.0054998	*TBCK*
rs138663011	11	68106952	G	A	3.29E-07	6.94E-06	*LRP5*
rs118173208	15	72043489	G	A	3.44E-07	0.0111694	*THSD4*
rs12693208	2	152875657	C	T	3.44E-07	9.63E-05	*CACNB4*
rs145040582	1	105661128	AC	A	3.46E-07	0.0157882	*RP11-414B7.1*
rs59228887	4	107273592	A	G	3.50E-07	0.00618928	*AIMP1*
rs3762945	4	107235064	C	T	3.50E-07	0.00546816	*TBCK*
rs7674460	4	107275156	T	A	3.53E-07	0.00621397	*AIMP1*
rs140904574	8	100798178	T	C	3.55E-07	5.02E-07	*VPS13B*
rs78685486	20	16731073	A	T	3.63E-07	0.000182559	*OTOR*
rs11930970	4	107276962	A	G	3.71E-07	0.00634853	*AIMP1*
rs374289606	15	100763076	A	G	3.71E-07	0.0463478	*ADAMTS17*
15:100763069_AG_A	15	100763069	A	AG	3.73E-07	0.0463477	/
rs142796415	3	194590997	A	C	3.75E-07	0.0106205	*XXYLT1*
rs1014966	6	5452328	T	G	3.75E-07	1.04E-06	*FARS2*
rs61256759	4	107234345	A	C	3.78E-07	0.00544355	*TBCK*
rs560375417	4	142366402	A	T	3.81E-07	0.00272159	*IL15*
rs7678749	4	107278978	C	T	3.82E-07	0.00698302	*AIMP1*
rs998914	3	112949081	A	G	3.82E-07	3.85E-05	*BOC*
rs12500295	4	107278166	G	C	3.85E-07	0.00703886	*AIMP1*
rs147640667	17	29581211	A	G	3.85E-07	0.0221269	*NF1*
rs10185392	2	143541696	A	G	3.85E-07	1.79E-05	*AC092578.1*
rs12645490	4	107279482	A	T	3.86E-07	0.00698731	*AIMP1*
rs189170779	16	50459358	C	A	3.87E-07	0.000135268	*NKD1*
rs75300145	6	5451845	C	T	3.90E-07	1.09E-06	*FARS2*
rs73839455	4	107281769	C	T	3.93E-07	0.00624417	*AIMP1*
rs73839422	4	107233658	C	T	3.93E-07	0.0053879	*TBCK*
rs6814166	4	107237395	T	C	3.94E-07	0.00568388	*TBCK*
rs62155760	2	84407782	G	C	3.95E-07	6.55E-06	*FUNDC2P2*
rs73839456	4	107281995	C	A	3.96E-07	0.0062079	*AIMP1*
rs7678943	4	107279097	A	T	3.99E-07	0.00656949	*AIMP1*
rs73719611	6	5457496	C	T	3.99E-07	3.91E-07	*FARS2*
rs11004662	10	56757641	T	C	4.00E-07	0.000788493	*PCDH15*
rs11383965	4	107269579	CT	C	4.01E-07	0.0179387	*AIMP1*
rs116111867	4	106993180	T	C	4.10E-07	0.00601331	*TBCK*
rs200745531	2	71914197	AC	A	4.11E-07	0.0131143	*DYSF*
rs57648108	13	95328589	G	T	4.11E-07	0.0198272	*SOX21*
2:222590580_ATAT_A	2	222590580	A	ATAT	4.16E-07	0.000435331	/
rs74154263	10	102778137	A	G	4.23E-07	0.000406954	*PDZD7*
rs7654865	4	107279450	T	C	4.27E-07	0.00712765	*AIMP1*
rs11706373	3	75480481	T	C	4.27E-07	0.0259768	*FAM86DP*
rs17259572	4	106961234	A	G	4.39E-07	0.00744901	*TBCK*
rs140069950	10	16766411	C	T	4.41E-07	0.000299557	*RSU1*
rs2425505	20	41284782	C	T	4.51E-07	0.0023241	*PTPRT*
rs72876575	4	107194607	G	A	4.54E-07	0.0049337	*TBCK*

4:107226452_GAAT_G	4	107226452	G	GAAT	4.56E-07	0.00443467	/
rs34614728	8	137339378	T	C	4.60E-07	0.000363013	*KHDRBS3*
rs114696892	3	77398283	A	G	4.61E-07	3.37E-06	*ROBO2*
rs77210717	7	147222039	G	A	4.61E-07	7.25E-05	*CNTNAP2*
rs76781666	8	137339421	C	T	4.62E-07	0.000323276	*KHDRBS3*
rs78320450	8	137338984	A	T	4.62E-07	0.000365017	*KHDRBS3*
rs35677837	8	137339292	A	G	4.62E-07	0.000365018	*KHDRBS3*
rs115348194	8	137339135	T	G	4.62E-07	0.00036502	*KHDRBS3*
rs79204859	8	137339420	A	G	4.62E-07	0.000365908	*KHDRBS3*
rs2425504	20	41284750	C	T	4.62E-07	0.00237814	*PTPRT*
rs142051800	7	88422978	C	A	4.63E-07	6.12E-07	*ZNF804B*
rs117997371	11	1724442	T	C	4.63E-07	0.000119652	*KRTAP5-6*
rs7675728	4	106951814	T	G	4.64E-07	0.0104152	*TBCK*
rs9684658	4	107182809	T	C	4.69E-07	0.004856	*TBCK*
rs139856543	14	86806395	C	T	4.70E-07	7.98E-09	*FLRT2*
rs111887782	3	66716574	C	G	4.72E-07	0.000128699	*KBTBD8*
rs111702699	7	20287336	T	C	4.74E-07	0.00265207	*ITGB8*
rs151245473	3	30689332	T	G	4.75E-07	0.0168623	*TGFBR2*
rs139403898	1	70001210	A	G	4.78E-07	0.00271872	*LRRC7*
rs372567922	8	13564360	CA	C	4.82E-07	0.00394788	*RP11-436P7.1*
rs73201271	4	2218685	G	A	4.84E-07	0.00234439	*POLN*
rs7668477	4	107133452	T	G	4.85E-07	0.00421943	*TBCK*
rs151147878	11	111676326	G	A	4.88E-07	0.00353711	*ALG9*
rs144643355	4	190296814	C	T	4.94E-07	0.00236709	*RP11-756P10.3*
rs114314455	4	190295131	T	C	4.94E-07	0.00236723	*RP11-756P10.3*
rs17036613	4	107133919	G	A	4.95E-07	0.00469798	*TBCK*
rs139017098	4	190256885	C	T	4.98E-07	0.00267694	*RP11-756P10.3*
rs77566898	4	190275760	A	G	4.98E-07	0.00238403	*RP11-756P10.3*
rs13178893	5	97936747	G	T	5.01E-07	0.00810805	*CTBP2P4*
rs79869782	3	127687765	G	A	5.01E-07	3.19E-06	*KBTBD12*
rs150793912	6	134283787	G	A	5.03E-07	1.27E-06	*TBPL1*
rs117294527	15	100764857	G	C	5.11E-07	0.0279588	*ADAMTS17*
rs190242095	4	190270325	A	G	5.17E-07	0.00309173	*RP11-756P10.3*
rs116299110	4	190259853	A	G	5.18E-07	0.00267587	*RP11-756P10.3*
rs537653234	4	190277007	A	G	5.18E-07	0.00295728	*RP11-756P10.3*
rs201889407	4	190275002	AT	A	5.18E-07	0.00300007	*RP11-756P10.3*
rs79022281	4	190280594	T	C	5.18E-07	0.0023854	*RP11-756P10.3*
rs138320087	4	190248758	A	C	5.19E-07	0.00267872	*RP11-756P10.3*
rs4926714	1	48512198	A	T	5.20E-07	0.020743	*CYP46A4P*
rs151144493	6	21731032	G	T	5.22E-07	5.69E-06	*RP11-524C21.1*
rs4645291	4	107231783	C	T	5.26E-07	0.00546475	*TBCK*
rs138282012	4	107219776	CA	C	5.28E-07	0.00679005	*TBCK*
rs145212500	3	80724920	A	G	5.32E-07	0.000312568	*RP11-481N16.1*
rs9565356	13	78159624	G	A	5.32E-07	2.28E-05	*SCEL*
4:190302073_CAG_C	4	190302073	C	CAG	5.41E-07	0.00246679	/
rs147263165	1	224666974	A	G	5.47E-07	0.00238656	*CNIH3*
rs10232445	7	26622985	A	G	5.50E-07	0.000220599	*KIAA0087*
rs760457	21	46329312	C	T	5.52E-07	2.66E-05	*ITGB2*
4:107202469_TTTG_T	4	107202469	T	TTTG	5.53E-07	0.0191777	/
rs12644596	4	107227180	T	C	5.59E-07	0.00531498	*TBCK*
rs112439126	4	107230534	T	A	5.61E-07	0.0053269	*TBCK*
rs4607300	4	107228521	C	T	5.71E-07	0.00533085	*TBCK*
rs6827816	4	107230363	C	T	5.73E-07	0.00534277	*TBCK*
rs17050261	2	59804348	C	G	5.78E-07	0.0166778	*BCL11A*
rs74285102	4	107227049	A	G	5.83E-07	0.00590382	*TBCK*

rs67417943	7	139006581	G	A	5.84E-07	0.0290948	*UBN2*
rs74132557	1	180134220	A	G	5.86E-07	0.019826	*QSOX1*
rs184858532	7	44908411	T	C	5.91E-07	0.00313825	*H2AFV*
rs79747906	18	3967852	C	T	5.98E-07	0.00688738	*DLGAP1*
rs566087594	8	61840272	G	T	5.99E-07	0.00150988	*AC022182.2*
rs12507867	4	106947003	G	A	6.00E-07	0.0116351	*TBCK*
rs62072466	17	18115544	A	T	6.09E-07	0.000206281	*ALKBH5*
rs73058158	3	36074147	T	A	6.20E-07	0.00227653	*AC104308.2*
rs77691720	6	5447345	A	G	6.26E-07	1.85E-06	*FARS2*
rs150011465	4	94502148	T	C	6.27E-07	0.000464064	*GRID2*
rs73172294	3	179440186	G	A	6.35E-07	0.000184722	*USP13*
rs62072468	17	18118179	G	A	6.35E-07	0.000221659	*ALKBH5*
rs115808848	1	117094189	A	G	6.36E-07	7.90E-05	*CD58*
rs77393553	1	48509897	C	G	6.41E-07	0.0207995	*CYP46A4P*
rs142664854	21	28083372	C	T	6.49E-07	9.03E-06	*ADAMTS1*
rs4926713	1	48506768	G	A	6.60E-07	0.0208126	*CYP46A4P*
rs4927319	1	48507085	A	G	6.63E-07	0.0208076	*CYP46A4P*
rs1563373	17	18114228	A	G	6.65E-07	0.0002226	*ALKBH5*
rs62072465	17	18114494	G	C	6.65E-07	0.0002226	*ALKBH5*
rs538443508	11	8727602	C	G	6.65E-07	1.66E-06	*DENND2B*
rs73058197	3	36103169	C	T	6.67E-07	0.00506914	*AC104308.2*
rs183319053	7	44910466	G	A	6.68E-07	0.00359287	*H2AFV*
rs73113148	7	44891158	G	A	6.69E-07	0.00361557	*H2AFV*
rs73113154	7	44894986	A	G	6.69E-07	0.00359508	*H2AFV*
rs7666823	4	163186240	G	A	6.70E-07	0.00468304	*FSTL5*
rs969262	20	41284395	G	T	6.72E-07	0.00889046	*PTPRT*
rs117962597	21	45621888	A	G	6.77E-07	7.81E-07	*ICOSLG*
1:48497395_AAGT_A	1	48497395	A	AAGT	6.89E-07	0.0206076	/
rs9308024	4	162207646	T	C	7.05E-07	0.000300133	*FSTL5*
rs115244735	4	35817219	G	A	7.07E-07	0.000580064	*ARAP2*
rs147818180	6	5446302	C	G	7.09E-07	2.14E-06	*FARS2*
rs4407172	19	55025471	A	C	7.16E-07	0.00620015	*LAIR2*
rs77029736	1	48493305	A	G	7.20E-07	0.0203021	*CYP46A4P*
rs35469407	5	26920982	A	G	7.22E-07	0.0210139	*CDH9*
rs151299022	1	117089537	A	G	7.29E-07	7.27E-05	*CD58*
rs80273221	6	5446023	G	C	7.30E-07	2.21E-06	*FARS2*
rs13225744	7	154521619	G	A	7.36E-07	0.0239472	*DPP6*
rs186678210	7	27335042	T	C	7.39E-07	4.70E-08	*RPL35P4*
rs144117640	4	162197644	A	G	7.43E-07	0.00311181	*FSTL5*
rs16960983	17	18104568	A	T	7.43E-07	0.000426292	*ALKBH5*
rs1891221	1	70006329	T	G	7.44E-07	0.00350854	*LRRC7*
rs115773911	2	159243289	T	C	7.45E-07	7.12E-07	*CCDC148*
rs150546162	4	60585598	A	G	7.48E-07	5.40E-06	*RP11-593F5.1*
rs77900939	15	100761083	G	A	7.51E-07	0.0236248	*ADAMTS17*
rs78834324	3	129162492	C	T	7.55E-07	0.0191031	*IFT122*
rs117460909	7	4244193	T	A	7.56E-07	3.36E-07	*SDK1*
rs631664	20	60940324	G	A	7.56E-07	0.029738	*LAMA5*
rs16960990	17	18108601	G	A	7.58E-07	0.000417733	*ALKBH5*
rs111589301	17	18116773	A	G	7.58E-07	0.000221969	*ALKBH5*
rs11657194	17	18092588	G	T	7.60E-07	0.000447662	*ALKBH5*
rs11653988	17	18088833	T	C	7.61E-07	0.000454754	*ALKBH5*
rs79963102	21	16471813	T	G	7.64E-07	0.00192288	*NRIP1*
rs6121995	20	60940004	T	C	7.70E-07	0.030108	*LAMA5*
rs8069094	17	18096528	T	C	7.81E-07	0.000439298	*ALKBH5*
rs72955190	2	59804805	G	T	7.86E-07	0.0078788	*BCL11A*
rs16960986	17	18108562	A	G	7.87E-07	0.000412427	*ALKBH5*
rs11655588	17	18107451	G	A	7.88E-07	0.000422895	*ALKBH5*

rs11655797	17	18107850	T	C	7.88E-07	0.000422895	*ALKBH5*
rs58441148	17	18106498	A	T	7.88E-07	0.000425809	*ALKBH5*
rs62072464	17	18108314	C	T	7.89E-07	0.000423241	*ALKBH5*
rs11868832	17	18105547	G	A	7.90E-07	0.000426066	*ALKBH5*
rs59602121	2	59805438	G	A	7.90E-07	0.00807529	*BCL11A*
rs16960980	17	18100858	A	G	7.91E-07	0.000431974	*ALKBH5*
rs11654177	17	18106214	T	C	7.91E-07	0.000425809	*ALKBH5*
rs11871491	17	18105656	C	T	7.93E-07	0.000426399	*ALKBH5*
10:56809968_AGGGTTAGAAGTCCT_A	10	56809968	A	AGGGTTAGAAGTCCT	7.94E-07	0.00139368	/
rs11078412	17	18100618	G	A	7.94E-07	0.000432339	*ALKBH5*
rs729269	7	154512110	A	G	8.00E-07	0.0179532	*DPP6*
rs77175620	18	69490258	T	C	8.04E-07	0.000364835	*RP11-723G8.2*
rs66482965	7	139009438	T	C	8.05E-07	0.0146322	*UBN2*
rs144127908	1	68569437	A	G	8.08E-07	0.00284302	*WLS*
rs75121681	1	180134075	A	G	8.09E-07	0.0181799	*QSOX1*
rs62525907	8	61840814	A	G	8.09E-07	0.00327516	*AC022182.2*
rs3767175	1	180156251	T	C	8.11E-07	0.015045	*QSOX1*
rs75281874	1	180161444	A	G	8.13E-07	0.0150861	*QSOX1*
rs62525909	8	61843039	C	A	8.14E-07	0.00350546	*AC022182.2*
rs769692383	2	152844791	C	CT	8.15E-07	0.00154654	*CACNB4*
rs3767181	1	180152242	A	G	8.15E-07	0.0150873	*QSOX1*
rs60285171	17	18110869	T	C	8.18E-07	0.000428779	*ALKBH5*
rs16960996	17	18110559	G	A	8.18E-07	0.000428749	*ALKBH5*
rs565395809	21	24722029	G	A	8.19E-07	3.97E-08	*EEF1A1P1*
rs3088233	17	18112675	T	C	8.20E-07	0.000425951	*ALKBH5*
rs13284	17	18112130	T	C	8.21E-07	0.000425878	*ALKBH5*
rs1563371	17	18111793	T	C	8.25E-07	0.000446681	*ALKBH5*
rs141657748	11	111694888	A	G	8.37E-07	0.00744281	*ALG9*
rs7249605	19	53485771	G	A	8.40E-07	3.53E-06	*ZNF702P*
rs140458168	2	106001208	G	A	8.42E-07	0.000228144	*FHL2*
rs7821208	8	61841372	G	A	8.44E-07	0.00340995	*AC022182.2*
rs11437627	4	107261439	GT	G	8.51E-07	0.00865221	*AIMP1*
rs62321990	4	95359772	C	G	8.51E-07	1.60E-06	*AC109925.1*
rs144331865	18	69482583	C	A	8.56E-07	0.000410314	*RP11-723G8.2*
rs143517325	5	119738957	G	C	8.58E-07	0.0163052	*PRR16*
rs73026472	6	170756716	A	T	8.59E-07	0.000415804	*PSMB1*
rs58023506	8	61841511	A	G	8.70E-07	0.00333771	*AC022182.2*
rs79569205	4	156760259	T	C	8.80E-07	1.42E-05	*ASIC5*
rs117116072	18	69562390	C	T	8.95E-07	0.000179982	*RP11-723G8.2*
rs56370372	17	11472961	T	C	8.98E-07	0.00497619	*DNAH9*
rs200610784	6	13512060	AT	A	9.01E-07	0.0206782	*GFOD1*
rs149012815	4	60583109	GT	G	9.10E-07	3.19E-06	*RP11-593F5.1*
rs143300216	17	21862339	G	T	9.11E-07	0.000101143	*RP11-744K17.3*
rs77972902	1	180131941	C	G	9.12E-07	0.0173526	*QSOX1*
rs9951772	18	3982583	T	A	9.17E-07	0.00432944	*DLGAP1*
rs80349135	3	156772055	A	G	9.20E-07	8.14E-06	*CCNL1*
rs6689602	1	180132688	G	A	9.21E-07	0.0172975	*QSOX1*
rs140784679	17	21865262	A	C	9.24E-07	0.00011303	*RP11-1109M24.11*
rs139211992	17	21868136	G	T	9.33E-07	0.000113498	*RP11-1109M24.11*
rs6502640	17	18122485	A	G	9.34E-07	0.000217177	*ALKBH5*
rs139597161	4	60595152	T	C	9.40E-07	5.10E-06	*RP11-593F5.1*
rs13235929	7	154520810	A	G	9.47E-07	0.0291461	*DPP6*
rs10015093	4	163190370	T	C	9.56E-07	0.00731326	*FSTL5*
rs12354230	1	180144240	T	C	9.62E-07	0.016054	*QSOX1*
rs842451	7	158568522	T	C	9.71E-07	0.0113699	*ESYT2*
rs13045735	20	60959890	G	A	9.75E-07	0.024939	*RPS21*

rs145065338	20	56649565	T	C	9.82E-07	9.33E-06	*PMEPA1*
rs12673237	7	154522065	T	G	9.86E-07	0.029306	*DPP6*
rs144285214	19	46742869	T	C	9.95E-07	0.0193801	*IGFL1*
rs76370724	10	92562753	C	CT	1.00E-06	0.00103335	*HTR7*
rs142964834	21	15231298	A	T	1.00E-06	0.0244121	*CYP4F29P*
rs78380970	2	59811622	A	C	1.01E-06	0.0103662	*BCL11A*
rs2460229	8	98446270	G	C	1.01E-06	0.0248176	*TSPYL5*
rs442140	1	40806933	A	T	1.01E-06	6.15E-06	*SMAP2*
rs10023120	4	163203871	T	C	1.01E-06	0.00736482	*FSTL5*
rs140064208	6	34182929	A	G	1.02E-06	0.000769809	*CYCSP55*
rs67460762	9	139416664	C	T	1.03E-06	2.04E-06	*NOTCH1*
rs116842049	17	21873326	T	C	1.04E-06	0.000113516	*RP11-1109M24.11*
rs140612790	17	21876163	T	C	1.04E-06	0.000113519	*RP11-1109M24.11*
rs138891644	17	21878027	T	A	1.04E-06	0.000113537	*RP11-1109M24.11*
rs142043739	17	21878585	A	G	1.04E-06	0.000113537	*RP11-1109M24.11*
rs142385086	17	21882021	A	G	1.04E-06	0.000113579	*RP11-1109M24.11*
rs151295969	17	21882032	T	G	1.04E-06	0.000113579	*RP11-1109M24.11*
rs61633784	10	1437844	G	A	1.05E-06	1.81E-05	*ADARB2*
rs191045897	17	21862107	C	T	1.06E-06	0.000101656	*RP11-744K17.3*
rs138278949	4	18356145	A	G	1.06E-06	1.07E-05	*LCORL*
rs184827310	8	100599731	G	A	1.06E-06	2.22E-06	*VPS13B*
rs145789946	17	21861989	G	C	1.06E-06	0.000101331	*RP11-744K17.3*
rs149445555	17	21861943	A	T	1.06E-06	0.000101211	*RP11-744K17.3*
rs116582181	17	21882632	T	C	1.06E-06	0.000113468	*RP11-1109M24.11*
rs117097386	17	21893125	T	C	1.07E-06	0.000115368	*RP11-1109M24.11*
rs36058389	17	18123434	T	G	1.07E-06	0.00021167	*ALKBH5*
rs377112003	17	21908928	T	G	1.07E-06	0.000122241	*RP11-1109M24.8*
rs570916079	17	21895373	ACTCTCT	A	1.08E-06	0.000124529	*RP11-1109M24.11*
rs2513390	8	98444949	A	G	1.08E-06	0.0254182	*TSPYL5*
rs148825205	17	21934763	T	C	1.09E-06	0.00012645	*RP11-744K17.7*
rs189087245	17	21898510	A	G	1.09E-06	0.000116625	*RP11-1109M24.11*
rs146041883	5	155013090	T	C	1.09E-06	0.0422103	*SGCD*
rs146758741	17	21899434	T	C	1.09E-06	0.000116787	*RP11-1109M24.11*
rs145696529	17	21899207	C	A	1.09E-06	0.000116787	*RP11-1109M24.11*
rs548446330	1	76046462	A	G	1.09E-06	4.88E-07	*SLC44A5*
rs118177269	17	21900951	T	C	1.10E-06	0.00011695	*RP11-1109M24.11*
rs117024651	17	21964839	G	C	1.10E-06	0.000123807	*RP11-744K17.8*
rs139419939	17	21910161	T	C	1.11E-06	0.000119069	*RP11-1109M24.8*
rs142641016	18	23651936	C	A	1.11E-06	0.0159521	*SS18*
rs117260520	17	21908588	A	G	1.11E-06	0.000118695	*RP11-1109M24.9*
rs56195588	4	162211709	A	T	1.11E-06	0.00108457	*FSTL5*
rs117979239	17	21910866	T	C	1.11E-06	0.000119265	*RP11-1109M24.8*
rs765039106	17	21885750	A	AT	1.11E-06	0.000126034	/
rs117055722	17	21909749	C	T	1.11E-06	0.000118892	*RP11-1109M24.8*
rs140173827	17	21913926	A	G	1.11E-06	0.000119468	*RP11-1109M24.7*
rs143397338	17	21913600	G	A	1.11E-06	0.000119468	*RP11-1109M24.7*
rs118133428	7	132063399	A	G	1.11E-06	0.00631105	*PLXNA4*
rs117524176	17	21968508	A	G	1.12E-06	0.000125666	*RP11-744K17.8*
rs117582270	17	21920177	G	A	1.12E-06	0.000120104	*RP11-744K17.7*
rs141023888	17	21918962	T	C	1.12E-06	0.000120104	*RP11-744K17.7*
rs142815952	17	21921600	T	C	1.12E-06	0.000120104	*RP11-744K17.7*
rs145410273	17	21929025	G	A	1.12E-06	0.000120103	*RP11-744K17.7*
rs145771934	17	21924866	C	G	1.12E-06	0.000120543	*RP11-744K17.7*
rs138179341	17	21948605	T	C	1.13E-06	0.000121169	*RP11-744K17.8*
rs149680517	17	21965891	A	G	1.13E-06	0.000128097	*RP11-744K17.8*
rs139195925	17	21939608	T	C	1.13E-06	0.000120812	*RP11-744K17.8*
rs146915687	17	21939603	G	A	1.13E-06	0.000120812	*RP11-744K17.8*

rs117645939	17	21934054	A	G	1.13E-06	0.000121533	*RP11-744K17.7*
rs144677893	17	21932933	A	C	1.13E-06	0.00012012	*RP11-744K17.7*
rs141378294	17	21944993	A	G	1.13E-06	0.000121166	*RP11-744K17.8*
rs117953217	17	21944474	T	C	1.13E-06	0.000121167	*RP11-744K17.8*
rs148021960	17	21948994	A	G	1.13E-06	0.000121169	*RP11-744K17.8*
rs117763222	17	21951917	A	G	1.13E-06	0.00012117	*RP11-744K17.8*
rs118119789	17	21953000	T	A	1.13E-06	0.000121174	*RP11-744K17.8*
rs149652401	17	21956759	C	T	1.13E-06	0.000121176	*RP11-744K17.8*
rs142644944	17	21959043	T	C	1.13E-06	0.000121177	*RP11-744K17.8*
rs117640518	17	21960412	A	G	1.13E-06	0.000121177	*RP11-744K17.8*
rs150081774	17	21961381	T	C	1.13E-06	0.000121179	*RP11-744K17.8*
1:157753232_GA_G	1	157753232	G	GA	1.13E-06	1.72E-07	/
rs183607234	17	21867720	C	G	1.13E-06	0.000126977	*RP11-1109M24.11*
rs56764036	8	61839895	A	G	1.14E-06	0.00333414	*AC022182.2*
rs139745416	1	44199508	T	C	1.14E-06	2.72E-06	*ST3GAL3*
rs3753809	1	180136711	C	T	1.15E-06	0.0193819	*QSOX1*
rs10808715	8	61849372	G	A	1.16E-06	0.00392194	*AC022182.2*
rs6536647	4	163200863	G	A	1.16E-06	0.00701847	*FSTL5*
8:61849486_CTT_C	8	61849486	C	CTT	1.16E-06	0.00383708	/
rs72828953	17	55250826	A	G	1.16E-06	0.00445275	*AKAP1*
rs17759374	7	29348878	T	G	1.17E-06	3.90E-09	*CHN2*
rs117428532	8	138142037	G	T	1.17E-06	9.99E-05	*KHDRBS3*
rs115369848	1	38041223	A	G	1.20E-06	0.0241189	*GNL2*
rs114252768	2	184014965	C	G	1.20E-06	0.00967177	*NUP35*
rs148387678	3	98381273	G	A	1.20E-06	0.0209398	*WWP1P1*
rs117736500	17	21964613	T	C	1.22E-06	0.000355207	*RP11-744K17.8*
rs11004690	10	56795648	T	C	1.22E-06	0.00108559	*PCDH15*
rs143041513	14	31606308	A	G	1.23E-06	0.00877775	*HECTD1*
rs551382133	12	20522146	T	C	1.25E-06	0.000131569	*PDE3A*
rs113612967	17	2717387	A	G	1.27E-06	0.000258631	*RAP1GAP2*
rs141840545	15	77370159	G	A	1.28E-06	0.0213531	*TSPAN3*
rs28782783	4	190214359	C	T	1.29E-06	0.000579889	*RP11-756P10.3*
rs139152084	7	29410546	C	T	1.30E-06	1.26E-07	*CHN2*
rs11527508	10	56831245	C	A	1.31E-06	0.00100092	*PCDH15*
rs2527697	7	22030060	T	C	1.33E-06	0.0104297	*RAPGEF5*
rs186806798	17	21972580	C	G	1.33E-06	0.000129437	*RP11-744K17.8*
rs28811342	17	18125845	C	T	1.33E-06	0.000233643	*LLGL1*
rs192117036	17	21972582	G	A	1.34E-06	0.00012815	*RP11-744K17.8*
rs142118282	4	190332676	A	G	1.35E-06	0.00609923	*RP11-756P10.3*
rs2107973	7	22029482	A	G	1.35E-06	0.010558	*RAPGEF5*
rs7802979	7	22030661	T	C	1.35E-06	0.010886	*RAPGEF5*
rs11371955	8	61840959	TA	T	1.35E-06	0.00425296	*AC022182.2*
rs62072498	17	18125951	G	C	1.35E-06	0.000238823	*LLGL1*
rs538446609	17	21853029	C	A	1.36E-06	0.000139803	*RP11-1109M24.12*
rs184617962	2	209643864	A	G	1.36E-06	1.24E-05	*PTH2R*
rs115424165	6	11234014	C	T	1.36E-06	1.21E-07	*NEDD9*
rs7424891	2	92282367	A	G	1.37E-06	4.47E-10	*IGKV1OR2-2*
rs11967988	6	7607292	T	C	1.37E-06	5.72E-06	*SNRNP48*
rs2107972	7	22029542	A	C	1.38E-06	0.0105403	*RAPGEF5*
rs577573266	7	111258850	A	C	1.38E-06	0.0232571	*DOCK4*
rs74586325	13	23813113	A	G	1.38E-06	0.00236837	*SGCG*
rs13225062	7	22030483	A	G	1.40E-06	0.0107197	*RAPGEF5*
rs148275067	17	21854349	T	C	1.42E-06	0.000140302	*RP11-744K17.3*
rs71636345	5	97982944	A	G	1.44E-06	0.00345832	*CTBP2P4*
rs9553426	13	25272264	T	C	1.44E-06	0.000168494	*ATP12A*
rs115549955	3	5678469	A	G	1.45E-06	0.0279164	*EDEM1*

rs149297758	1	222983281	T	C	1.46E-06	3.78E-05	*DISP1*
rs7821046	8	61841256	G	A	1.46E-06	0.00480656	*AC022182.2*
rs77061667	8	60016139	C	T	1.46E-06	0.000146138	*TOX*
rs79577665	4	134323986	T	G	1.49E-06	0.00992749	*PCDH10*
rs1224631	15	48123228	T	G	1.49E-06	0.00733633	*SEMA6D*
rs2853306	8	98435684	G	A	1.51E-06	0.0314457	*TSPYL5*
rs73022033	3	4403260	T	C	1.51E-06	0.0300118	*SUMF1*
rs6789501	3	66714241	A	G	1.51E-06	0.000192721	*KBTBD8*
rs117638454	21	27928148	A	C	1.52E-06	8.84E-06	*CYYR1*
rs141521629	10	119086303	T	C	1.55E-06	7.60E-05	*PDZD8*
rs17351328	18	74050217	T	C	1.55E-06	0.0059633	*ZNF516*
rs544791	15	48124888	G	A	1.56E-06	0.00676497	*SEMA6D*
rs143973615	17	21852172	A	C	1.56E-06	0.000149747	*RP11-1109M24.12*
rs11100423	4	163190862	T	G	1.57E-06	0.0112597	*FSTL5*
rs17363208	2	5913608	A	G	1.57E-06	5.65E-05	*SOX11*
12:52499485_TGAG_T	12	52499485	T	TGAG	1.59E-06	0.000832742	/
rs73182746	3	95069531	G	A	1.62E-06	0.00776803	*RPS18P6*
rs11057384	12	124370764	A	C	1.63E-06	0.0189107	*DNAH10*
rs200493526	5	24560457	CA	C	1.63E-06	0.0415834	*CDH10*
rs200486455	8	61849496	A	T	1.64E-06	0.00604208	*AC022182.2*
1:104453487_ATCT_A	1	104453487	A	ATCT	1.64E-06	9.71E-07	/
rs2513389	8	98436047	T	C	1.66E-06	0.0331334	*TSPYL5*
rs79058559	5	24553792	T	C	1.67E-06	0.0412992	*CDH10*
rs757944090	10	28457944	A	AAGG	1.67E-06	2.79E-08	/
rs17457952	5	24512495	A	G	1.68E-06	0.0413372	*CDH10*
rs182368518	4	161318293	T	C	1.68E-06	2.84E-05	*RP11-138A23.1*
rs75009488	12	67357682	T	A	1.69E-06	8.47E-08	*GRIP1*
rs551331	15	48124151	A	G	1.70E-06	0.00623182	*SEMA6D*
rs111891077	1	66362559	C	T	1.71E-06	0.0328069	*PDE4B*
rs9876236	3	195771574	A	G	1.72E-06	0.0229263	*TFRC*
rs142052557	5	24527643	T	C	1.72E-06	0.0413528	*CDH10*
rs75416251	11	111434033	C	A	1.72E-06	0.00127333	*LAYN*
rs115141923	4	161322910	C	T	1.74E-06	2.79E-05	*RP11-138A23.1*
17:21838690_TC_T	17	21838690	T	TC	1.75E-06	0.000162541	/
rs11481521	20	36150469	GA	G	1.75E-06	8.43E-06	*NNAT*
rs75217985	3	66713470	C	G	1.76E-06	0.000192032	*KBTBD8*
rs139174648	5	24519631	G	C	1.77E-06	0.0364255	*CDH10*
rs144660835	6	106914943	T	G	1.78E-06	0.00132721	*ATG5*
rs117183790	6	97236749	A	G	1.79E-06	8.85E-05	*GPR63*
rs2253994	8	98431841	A	G	1.83E-06	0.0348117	*TSPYL5*
rs73060169	3	36145416	T	C	1.85E-06	0.0203226	*AC104308.2*
rs116548736	2	145390250	T	C	1.86E-06	4.94E-06	*ZEB2*
rs201789575	18	40278396	GTTC	G	1.89E-06	6.57E-07	*RIT2*
rs115716251	4	35901444	A	T	1.89E-06	0.00446518	*ARAP2*
rs62073604	17	18065888	C	T	1.89E-06	0.000500125	*MYO15A*
rs145456621	17	21834777	T	C	1.89E-06	0.000168829	*RP11-1109M24.12*
rs9877409	3	4372386	G	C	1.89E-06	0.00293235	*SUMF1*
rs118018177	17	21835517	C	T	1.90E-06	0.000157979	*RP11-1109M24.12*
rs3753805	1	180138890	A	G	1.90E-06	0.0171298	*QSOX1*
rs3905359	10	56825358	C	G	1.91E-06	0.00108145	*PCDH15*
rs117973853	6	150993301	T	C	1.91E-06	1.43E-06	*PLEKHG1*
rs116928215	17	21834404	T	C	1.91E-06	0.000158774	*RP11-1109M24.12*
rs62073615	17	18080329	A	T	1.92E-06	0.000724626	*MYO15A*
rs12660004	5	26919924	T	A	1.92E-06	0.0359669	*CDH9*
rs117398415	12	124374596	G	A	1.92E-06	0.0308803	*DNAH10*

rs1996561	8	98425214	G	C	1.92E-06	0.032016	*TSPYL5*
8:98421668_GC_G	8	98421668	G	GC	1.92E-06	0.0328591	/
rs2853300	8	98424479	G	A	1.92E-06	0.0322248	*TSPYL5*
rs114345502	4	32204514	C	G	1.93E-06	0.000101082	*PCDH7*
rs4783897	16	55672391	A	G	1.94E-06	5.55E-05	*LPCAT2*
rs146558436	16	73071025	T	C	1.94E-06	3.88E-07	*ZFHX3*
rs117282643	12	124371614	T	C	1.95E-06	0.0207071	*DNAH10*
rs182227893	17	21819095	C	T	1.96E-06	0.000160963	*RP11-1109M24.14*
rs139297030	17	21818953	A	G	1.97E-06	0.000160252	*RP11-1109M24.14*
rs149743478	17	21820613	A	G	1.97E-06	0.000160242	*RP11-1109M24.14*
rs60108251	3	127176185	T	G	1.97E-06	3.24E-05	*TPRA1*
rs147805212	17	21822004	C	G	1.98E-06	0.00016071	*RP11-1109M24.14*
rs117212055	17	21822496	G	A	1.98E-06	0.000161107	*RP11-1109M24.14*
rs117888986	17	21822436	A	C	1.98E-06	0.000161107	*RP11-1109M24.14*
12:120589817_GTAAAGTTTTT_G	12	120589817	G	GTAAAGTTTTT	1.98E-06	4.58E-06	/
rs562543261	4	9763318	T	C	2.01E-06	0.0108492	*SLC2A9*
rs17712413	12	116166767	C	A	2.01E-06	3.35E-05	*MED13L*
rs75746785	1	237157093	A	C	2.01E-06	0.00519811	*RPL35P1*
rs77714634	4	32178756	G	A	2.02E-06	0.000115166	*PCDH7*
rs8077577	17	18064730	T	C	2.03E-06	0.000429073	*MYO15A*
rs80024556	9	101172602	A	G	2.03E-06	7.10E-06	*GABBR2*
rs62072500	17	18126918	C	A	2.04E-06	0.000481799	*LLGL1*
rs141908214	4	32197099	T	C	2.06E-06	0.000108713	*PCDH7*
rs114020042	12	116171106	A	G	2.06E-06	3.86E-05	*MED13L*
rs117289863	13	71833628	C	T	2.07E-06	0.0411585	*RABEPKP1*
rs76938823	12	116164426	T	C	2.07E-06	3.78E-05	*MED13L*
rs780471355	9	78011618	T	G	2.07E-06	0.011175	*OSTF1*
rs147887460	16	50487127	G	C	2.09E-06	7.12E-06	*NKD1*
rs138264396	17	21827916	C	T	2.11E-06	0.000168619	*RP11-1109M24.14*
rs149859933	15	75294000	G	A	2.11E-06	1.18E-05	*SCAMP5*
rs34068710	3	150670023	G	A	2.13E-06	1.97E-06	*CLRN1*
rs735960	17	18057301	A	G	2.13E-06	0.000293739	*MYO15A*
rs62520285	8	134518585	T	C	2.13E-06	0.0121885	*ST3GAL1*
4:187589928_CA_C	4	187589928	C	CA	2.16E-06	0.000392291	/
rs143129166	12	116173447	G	A	2.18E-06	3.55E-05	*MED13L*
rs17761602	22	47064075	A	G	2.18E-06	3.23E-06	*GRAMD4*
rs17058043	6	98251106	A	G	2.20E-06	0.00226571	*MMS22L*
rs185384447	3	158002973	T	G	2.20E-06	0.00763053	*RSRC1*
rs185140140	3	39474595	C	T	2.20E-06	0.00273251	*RPSA*
rs17439972	9	131948849	G	C	2.20E-06	0.000320813	*IER5L*
rs140256119	4	107135163	C	T	2.24E-06	0.0210615	*TBCK*
rs542568028	3	118324656	G	T	2.25E-06	1.68E-06	*IGSF11*
rs62362983	5	75976373	T	G	2.26E-06	2.37E-05	*IQGAP2*
rs11652964	17	18040404	T	C	2.27E-06	0.00022064	*MYO15A*
rs147997219	19	49004917	T	C	2.30E-06	0.000143044	*LMTK3*
rs139036859	13	64886312	C	T	2.31E-06	0.0446888	*LGMNP1*
rs118139102	12	116156297	A	G	2.31E-06	3.95E-05	*MED13L*
rs7207276	17	18065737	C	G	2.32E-06	0.000498333	*MYO15A*
17:18065210_AGG_A	17	18065210	A	AGG	2.36E-06	0.000484871	/
rs76682220	2	221127984	A	C	2.39E-06	0.0369252	*AC009310.1*
rs143302730	9	113985535	G	T	2.39E-06	0.0015425	*OR2K2*
rs79144486	10	134771136	A	G	2.40E-06	1.26E-08	*CFAP46*
rs2015336	17	18073610	G	A	2.40E-06	0.000499644	*MYO15A*

rs77198097	12	124371219	G	A	2.44E-06	0.0243179	*DNAH10*
rs72842176	2	127914819	A	G	2.44E-06	0.0143276	*BIN1*
rs62073608	17	18071448	C	T	2.44E-06	0.000517552	*MYO15A*
rs11044441	12	19322754	A	G	2.45E-06	0.029887	*PLEKHA5*
rs62073609	17	18071843	C	T	2.45E-06	0.000522881	*MYO15A*
rs77604835	3	81596421	A	G	2.47E-06	0.000584065	*GBE1*
rs55918833	17	18072352	C	T	2.50E-06	0.000534446	*MYO15A*
rs540068909	7	140062755	T	C	2.50E-06	7.59E-06	*SLC37A3*
rs145246408	17	21844669	G	A	2.51E-06	0.000190231	*RP11-1109M24.12*
rs139160329	10	22786587	G	A	2.51E-06	0.0169023	*SPAG6*
rs141719596	1	214641197	T	C	2.53E-06	1.23E-05	*PTPN14*
rs117668764	17	21842637	T	C	2.56E-06	0.000207169	*RP11-1109M24.12*
rs17223096	6	133958425	G	T	2.56E-06	1.26E-05	*EYA4*
rs146671994	6	151138945	A	G	2.56E-06	0.000487158	*PLEKHG1*
rs75830586	1	91095978	G	A	2.58E-06	0.0302187	*BARHL2*
rs150309325	9	108170930	A	G	2.61E-06	0.03266	*SLC44A1*
rs141796363	3	118783696	C	T	2.62E-06	0.000119458	*IGSF11*
rs997315	17	18085286	G	A	2.66E-06	0.000674196	*ALKBH5*
rs80221554	16	55098704	T	C	2.66E-06	3.02E-05	*IRX6*
rs11656035	17	18086025	C	T	2.66E-06	0.000673038	*ALKBH5*
rs34474249	17	18074525	C	T	2.67E-06	0.000581504	*MYO15A*
rs746165404	13	95326699	TAT	T	2.68E-06	0.0192944	*SOX21*
rs62073605	17	18066791	T	C	2.68E-06	0.000748083	*MYO15A*
rs147989671	14	52856758	A	G	2.68E-06	1.48E-05	*TXNDC16*
rs62073606	17	18069142	A	G	2.68E-06	0.000729256	*MYO15A*
rs116997290	8	60308445	C	T	2.69E-06	0.0425618	*TOX*
rs11654146	17	18076141	C	A	2.71E-06	0.000593178	*MYO15A*
rs58673429	1	50718961	T	C	2.71E-06	0.010103	*ELAVL4*
rs113522391	3	120459791	C	T	2.72E-06	4.16E-06	*RABL3*
rs12136109	1	4243708	T	A	2.72E-06	0.0239634	*AJAP1*
rs62073649	17	18080522	C	T	2.73E-06	0.000653826	*MYO15A*
rs10048161	17	18082796	G	C	2.75E-06	0.000608678	*MYO15A*
21:28071954_AC_A	21	28071954	A	AC	2.77E-06	0.00181309	/
rs11658477	17	18044893	A	G	2.81E-06	0.000274115	*MYO15A*
rs62073603	17	18064316	T	C	2.84E-06	0.000524557	*MYO15A*
rs13425303	2	221125519	T	C	2.86E-06	0.0329356	*AC009310.1*
rs17675868	1	200126078	C	T	2.87E-06	0.00184285	*NR5A2*
rs71893067	10	1509239	ATC	A	2.87E-06	0.00112973	/
rs75157267	9	73018762	C	T	2.87E-06	0.000290478	*KLF9*
rs116527637	1	5748645	C	G	2.87E-06	0.000122487	*NPHP4*
rs11655731	17	18107525	T	G	2.89E-06	0.000887826	*ALKBH5*
rs11868886	17	18105585	A	G	2.90E-06	0.000895193	*ALKBH5*
rs79500077	13	71840131	G	A	2.91E-06	0.0295434	*RABEPKP1*
rs111974547	1	8184089	C	T	2.91E-06	0.00457077	*RP11-431K24.2*
rs76764676	2	145344078	A	G	2.92E-06	4.17E-06	*ZEB2*
rs1341161	10	96724061	T	C	2.94E-06	0.00831741	*CYP2C9*
rs149922460	17	21805390	G	A	2.96E-06	0.000179561	*RP11-1109M24.15*
rs11745250	5	66657771	A	G	2.98E-06	5.73E-06	*CD180*
rs144849842	1	214613536	T	C	3.01E-06	1.53E-05	*PTPN14*
rs4839875	6	97268615	A	T	3.01E-06	2.30E-06	*GPR63*
rs371407211	6	140769067	CT	C	3.02E-06	2.47E-05	/
rs77497601	4	15748053	G	T	3.05E-06	4.26E-07	*RP11-442P12.2*
rs146166536	4	7156799	G	C	3.05E-06	7.64E-06	*GRPEL1*
rs62541805	9	36269958	T	C	3.06E-06	0.0163312	*GNE*
rs142108158	17	21805668	T	C	3.07E-06	0.000184284	*RP11-1109M24.15*
rs72848807	2	128587108	G	A	3.07E-06	0.000898737	*WDR33*
rs144326611	9	36257538	T	C	3.07E-06	0.0162578	*CLTA*

rs185728293	17	21815113	A	G	3.09E-06	0.000159895	*RP11-1109M24.14*
rs116085930	3	62031671	A	T	3.12E-06	0.00399728	*PTPRG*
rs78920283	17	10920238	A	G	3.13E-06	0.000101438	*SHISA6*
rs55951301	1	112326415	ATGT	A	3.13E-06	0.00751377	*KCND3*
rs56392653	1	112326416	ATC	A	3.13E-06	0.00751377	*KCND3*
rs745738085	1	112326417	CA	C	3.13E-06	0.00751377	*KCND3*
rs77162969	17	21831436	T	C	3.14E-06	0.000218831	*RP11-1109M24.13*
rs71306535	3	150695107	T	C	3.15E-06	1.40E-08	*CLRN1*
rs62073611	17	18071961	G	C	3.15E-06	0.000788659	*MYO15A*
rs141712749	17	21831848	T	C	3.16E-06	0.000218568	*RP11-1109M24.13*
rs11698467	20	60934947	T	C	3.16E-06	0.0227405	*LAMA5*
rs143380218	3	13369787	T	C	3.18E-06	2.81E-05	*NUP210*
rs117474622	17	21830267	A	G	3.19E-06	0.000219351	*RP11-1109M24.13*
8:69312629_TTTCCCCAGCACTATTTATTGCAAAG_T	8	69312629	T	TTTCCCCAGCACTATTTATTGCAAAG	3.25E-06	3.65E-06	/
rs145246669	9	38734739	A	G	3.25E-06	1.10E-05	*VN2R3P*
rs7965360	12	48259382	G	A	3.27E-06	0.000714867	*VDR*
rs144397327	18	75587176	T	C	3.30E-06	0.0270465	*SALL3*
rs4847086	1	105641626	A	T	3.31E-06	0.0145274	*RP11-414B7.1*
rs116561305	5	1554423	G	C	3.32E-06	0.01862	*LPCAT1*
rs4463677	1	104380531	T	C	3.32E-06	2.51E-06	*AMY1C*
1:104435616_TA_T	1	104435616	T	TA	3.34E-06	1.55E-06	/
rs12190193	6	170757021	T	C	3.35E-06	0.0347419	*PSMB1*
10:121171354_CTTTTTTTTT_C	10	121171354	C	CTTTTTTTTT	3.36E-06	0.0226197	/
rs61874594	10	114343260	A	G	3.37E-06	3.72E-05	*VTI1A*
rs191227827	14	53240085	T	C	3.38E-06	4.32E-06	*STYX*
rs146547665	3	13377882	T	C	3.38E-06	2.76E-05	*NUP210*
rs199857577	9	26001632	CT	C	3.40E-06	0.0132073	*CAAP1*
rs3842814	13	24938062	A	C	3.40E-06	0.00011343	*CYCSP33*
rs34510512	21	16956149	G	A	3.41E-06	0.000281374	*CYCSP42*
rs6856	11	62458275	C	T	3.44E-06	5.21E-05	*BSCL2*
rs7208839	17	21842191	A	G	3.44E-06	0.000204501	*RP11-1109M24.12*
3:82724068_GT_G	3	82724068	G	GT	3.44E-06	0.0300637	/
rs80062084	3	157945439	T	C	3.49E-06	0.00638294	*RSRC1*
rs1499269	4	129218494	G	A	3.51E-06	3.56E-07	*JADE1*
rs11871039	17	18078904	G	C	3.52E-06	0.000904901	*MYO15A*
rs1488930	7	154523235	G	A	3.53E-06	0.025134	*DPP6*
rs189114639	13	70391130	T	A	3.53E-06	0.000396234	*KLHL1*
rs142666276	15	77398996	G	A	3.54E-06	0.0126655	*PEAK1*
rs117794975	6	97235850	C	T	3.55E-06	0.000754019	*GPR63*
rs6544086	2	37729133	A	G	3.57E-06	0.0304477	*QPCT*
rs111840366	11	40522917	A	G	3.58E-06	6.18E-06	*LRRC4C*
rs12600694	17	18091019	A	G	3.60E-06	0.0017753	*ALKBH5*
rs112740591	1	180164132	GGGT	G	3.61E-06	0.0212269	*QSOX1*
rs112384084	3	195594494	T	C	3.61E-06	4.10E-05	*TNK2*
rs12034806	1	4239514	T	C	3.61E-06	0.0254369	*RP11-374C13.1*
rs115899785	1	37668102	T	C	3.62E-06	0.000548945	*GRIK3*
rs71433207	13	27505413	A	T	3.64E-06	8.76E-07	*RPS21P8*
rs2714445	7	79878921	T	C	3.65E-06	0.00236308	*GNAI1*
rs115022648	1	166012729	T	A	3.66E-06	0.00911424	*FAM78B*
rs145870286	6	134258662	A	G	3.66E-06	3.13E-06	*TBPL1*
1:156355248_CA_C	1	156355248	C	CA	3.70E-06	0.0107871	/

rs12782764	10	1509658	A	G	3.74E-06	0.00207626	*ADARB2*
rs115454719	4	35813832	T	G	3.75E-06	0.000424804	*ARAP2*
rs9332235	10	96747905	A	G	3.75E-06	0.00781594	*CYP2C9*
rs112556211	18	12590378	T	C	3.76E-06	0.0211043	*SPIRE1*
12:97567137_CT_C	12	97567137	C	CT	3.79E-06	6.62E-05	/
rs185728410	7	62199206	C	T	3.80E-06	0.0375257	*RP11-196D18.2*
rs138552519	17	21838567	A	G	3.81E-06	0.000269981	*RP11-1109M24.12*
rs115325182	3	19247662	G	A	3.82E-06	0.000389607	*KCNH8*
rs138576032	3	158058724	C	G	3.85E-06	0.00753425	*RSRC1*
3:104913189_AC_A	3	104913189	A	AC	3.85E-06	0.0216009	/
6:40224564_TC_T	6	40224564	T	TC	3.86E-06	1.09E-06	/
rs143400353	5	86770691	A	G	3.87E-06	0.000655425	*CCNH*
rs55894187	3	82700245	A	G	3.89E-06	0.0278531	*CYP51A1P1*
rs112255063	9	1674294	G	A	3.89E-06	0.0222613	*RP11-443B9.1*
rs73132167	3	82730257	T	A	3.89E-06	0.028771	*CYP51A1P1*
rs62072496	17	18124743	A	G	3.91E-06	0.000441702	*LLGL1*
rs142547867	11	40543645	T	C	3.91E-06	5.53E-06	*LRRC4C*
rs72641981	13	88627047	G	A	3.92E-06	4.51E-07	*RPL29P29*
rs72929424	11	62462546	G	C	3.93E-06	6.98E-05	*BSCL2*
12:9458100_TGAG_T	12	9458100	T	TGAG	3.93E-06	6.63E-05	/
rs114738411	1	112304164	C	T	3.94E-06	0.00370394	*DDX20*
rs532147141	12	20756879	T	C	3.95E-06	0.00223463	*PDE3A*
rs72841922	2	84338670	G	T	3.95E-06	0.000868603	*FUNDC2P2*
18:9112916_AAT_A	18	9112916	A	AAT	3.95E-06	7.24E-05	/
1:104423508_TA_T	1	104423508	T	TA	3.97E-06	2.55E-06	/
rs78949801	2	60623902	T	C	3.97E-06	2.02E-05	*BCL11A*
rs61851649	10	73283718	A	G	3.98E-06	0.00401677	*CDH23*
rs2192941	2	37718835	C	T	4.00E-06	0.0297967	*QPCT*
rs61333033	3	36459975	C	T	4.01E-06	0.00162946	*STAC*
1:104487662_GAGACA_G	1	104487662	G	GAGACA	4.01E-06	1.62E-06	/
rs62072497	17	18125877	A	G	4.02E-06	0.000431081	*LLGL1*
rs56711178	1	105661129	A	T	4.02E-06	0.0166588	*RP11-414B7.1*
rs61217171	1	105661130	A	T	4.02E-06	0.0166588	*RP11-414B7.1*
rs41269369	1	239866503	G	A	4.02E-06	0.00574836	*CHRM3*
rs72694446	1	104396377	G	A	4.02E-06	2.38E-06	*AMY1C*
rs11231193	11	62466992	C	T	4.03E-06	6.97E-05	*BSCL2*
rs145898293	10	4517519	C	T	4.03E-06	0.0153189	*KLF6*
rs58856600	1	104443263	T	G	4.05E-06	1.98E-06	*AMY1C*
rs72698796	14	101363503	T	C	4.05E-06	1.72E-05	*AL117190.3*
rs75935513	12	124374598	G	A	4.06E-06	0.0304424	*DNAH10*
rs34203236	10	1509191	A	G	4.06E-06	0.00219569	*ADARB2*
rs567644317	2	190781057	A	T	4.08E-06	0.00327691	*C2orf88*
rs72694443	1	104388206	T	G	4.08E-06	2.40E-06	*AMY1C*
rs2699462	7	22031326	A	G	4.09E-06	0.0242557	*RAPGEF5*
rs142627474	2	184080053	C	T	4.10E-06	0.00709418	*NUP35*
rs17035045	3	36458103	T	C	4.10E-06	0.00178734	*STAC*
rs532826702	3	84139176	T	C	4.13E-06	7.12E-06	*RP11-651J20.1*
rs8065026	17	18046290	C	T	4.14E-06	0.000158724	*MYO15A*
rs146390284	10	96718010	C	T	4.15E-06	0.00542015	*CYP2C9*
rs150033649	17	72134441	T	A	4.16E-06	2.13E-07	*SDK2*
rs78254452	2	60629051	G	T	4.18E-06	2.04E-05	*BCL11A*

rs12031950	1	4236710	T	C	4.18E-06	0.0256174	*RP11-374C13.1*
rs2527701	7	22031291	T	G	4.18E-06	0.024361	*RAPGEF5*
1:167211411_C ATTA_C	1	167211411	C	CATTA	4.18E-06	4.93E-06	/
rs12032955	1	4237878	T	C	4.19E-06	0.0255599	*RP11-374C13.1*
rs7935127	11	62470847	A	G	4.19E-06	7.30E-05	*BSCL2*
rs61931467	12	115412447	C	T	4.19E-06	0.0122838	*TBX3*
rs12424638	12	49318800	A	G	4.21E-06	0.0122618	*FKBP11*
rs34230456	1	4238472	A	G	4.21E-06	0.0254796	*RP11-374C13.1*
rs112714735	8	117627985	T	C	4.21E-06	3.42E-05	*TRPS1*
rs11523848	10	82505388	T	C	4.22E-06	0.00147782	*RP11-20E23.1*
rs2527700	7	22030951	T	C	4.22E-06	0.024071	*RAPGEF5*
rs1871020	3	82717048	C	T	4.23E-06	0.0292031	*CYP51A1P1*
rs12032975	1	4238038	T	C	4.25E-06	0.0255404	*RP11-374C13.1*
rs189554824	3	62047404	C	G	4.25E-06	0.00319999	*PTPRG*
rs11581912	1	104452251	A	G	4.25E-06	2.46E-06	*AMY1C*
rs147909535	13	34740757	T	C	4.25E-06	2.69E-07	*VDAC1P12*
rs12030172	1	4237984	T	G	4.25E-06	0.025581	*RP11-374C13.1*
rs9628997	1	104453628	T	G	4.26E-06	2.47E-06	*AMY1C*
rs72679982	1	104451404	A	G	4.26E-06	2.46E-06	*AMY1C*
rs72694458	1	104412792	G	A	4.26E-06	2.43E-06	*AMY1C*
rs74732460	1	104412357	T	A	4.26E-06	2.43E-06	*AMY1C*
rs72694456	1	104405613	A	C	4.26E-06	2.43E-06	*AMY1C*
rs72694457	1	104409140	A	T	4.26E-06	2.43E-06	*AMY1C*
rs113991964	7	146109285	G	A	4.26E-06	5.90E-05	*CNTNAP2*
rs72694451	1	104401791	A	G	4.27E-06	2.44E-06	*AMY1C*
rs72694454	1	104404783	A	G	4.27E-06	2.43E-06	*AMY1C*
rs112438207	1	104417312	T	G	4.28E-06	2.44E-06	*AMY1C*
rs72694461	1	104416516	A	G	4.28E-06	2.44E-06	*AMY1C*
rs72929434	11	62469929	A	C	4.28E-06	7.00E-05	*BSCL2*
rs75661393	5	86655947	G	T	4.29E-06	3.36E-06	*RASA1*
rs113199326	1	104448207	G	A	4.29E-06	2.45E-06	*AMY1C*
rs41511047	1	104439144	A	G	4.30E-06	2.45E-06	*AMY1C*
rs72679977	1	104436384	T	C	4.30E-06	2.45E-06	*AMY1C*
rs72679978	1	104437949	T	C	4.30E-06	2.45E-06	*AMY1C*
rs72679975	1	104433592	T	C	4.30E-06	2.45E-06	*AMY1C*
rs13374153	1	104432003	T	C	4.30E-06	2.45E-06	*AMY1C*
rs13374158	1	104432076	G	C	4.30E-06	2.45E-06	*AMY1C*
rs75626367	6	97234591	A	G	4.30E-06	0.000971691	*GPR63*
rs72679979	1	104438156	C	A	4.30E-06	2.45E-06	*AMY1C*
rs72679976	1	104434536	T	A	4.30E-06	2.45E-06	*AMY1C*
rs13376648	1	104430434	A	G	4.31E-06	2.45E-06	*AMY1C*
rs13374076	1	104430099	G	A	4.31E-06	2.45E-06	*AMY1C*
rs11589939	1	104414029	G	A	4.31E-06	2.45E-06	*AMY1C*
rs72694464	1	104423351	T	C	4.31E-06	2.45E-06	*AMY1C*
rs36075516	10	52109311	TC	T	4.33E-06	0.00426559	*SGMS1*
rs752274458	20	40401611	T	TG	4.34E-06	0.0377987	*CHD6*
rs72698664	9	12365393	G	T	4.35E-06	0.00470718	*TYRP1*
rs4678874	3	36457372	G	A	4.37E-06	0.00178524	*STAC*
rs143564659	20	17884894	A	G	4.38E-06	3.19E-06	*SNX5*
rs117644056	8	60389355	C	T	4.39E-06	0.02688	*TOX*
rs2373111	2	37730983	G	C	4.39E-06	0.0323389	*QPCT*
rs151171352	4	122288378	T	C	4.40E-06	0.00255089	*QRFPR*
rs117180979	12	80090373	C	T	4.40E-06	0.0167102	*PPP1R12A*
rs150640848	8	138210006	C	A	4.41E-06	0.000114855	*KHDRBS3*
rs117444449	16	51816056	A	G	4.42E-06	1.82E-06	*HNRNPA1P48*
rs11762281	7	22031967	T	A	4.43E-06	0.0334935	*RAPGEF5*
rs72694452	1	104402981	A	G	4.44E-06	2.56E-06	*AMY1C*

rs12047711	1	105660547	T	C	4.44E-06	0.0226637	*RP11-414B7.1*
rs6677692	1	105633111	A	C	4.49E-06	0.0140887	*RP11-414B7.1*
rs11583141	1	105654287	A	G	4.49E-06	0.0227398	*RP11-414B7.1*
rs12270593	11	62499998	T	C	4.50E-06	7.06E-05	*TTC9C*
rs7415977	1	105633335	C	T	4.51E-06	0.0140891	*RP11-414B7.1*
rs56135662	11	62473548	T	C	4.51E-06	7.01E-05	*BSCL2*
rs11588494	1	104410340	A	G	4.53E-06	2.79E-06	*AMY1C*
rs73199884	12	111791185	A	G	4.56E-06	0.000123447	*CUX2*
rs7698238	4	63056880	T	C	4.56E-06	0.0166074	*RP11-84A1.1*
rs8117723	20	40399442	A	G	4.59E-06	0.0312118	*CHD6*
rs6692674	1	105632468	A	C	4.61E-06	0.0136162	*RP11-414B7.1*
rs141146249	12	7882578	G	A	4.65E-06	0.00259754	*CLEC4C*
rs61744696	9	138664762	T	C	4.66E-06	7.70E-05	*KCNT1*
rs55707677	11	62506718	G	A	4.67E-06	7.94E-05	*TTC9C*
rs114678891	6	40231985	C	G	4.67E-06	2.86E-07	*LRFN2*
rs145342318	4	107135174	A	G	4.67E-06	0.022762	*TBCK*
rs138826256	9	2025808	A	G	4.68E-06	2.96E-05	*SMARCA2*
rs56160161	3	112572246	T	C	4.70E-06	2.79E-06	*CD200R1*
rs7959305	12	80070534	T	C	4.70E-06	0.0168086	*PAWR*
rs143729540	17	18268113	G	C	4.70E-06	0.0486203	*SHMT1*
rs59366579	17	18161744	T	C	4.72E-06	0.0256859	*FLII*
rs139661527	17	21803290	T	C	4.73E-06	0.000217001	*RP11-1109M24.15*
rs150500972	18	58533335	G	A	4.73E-06	6.31E-05	*CDH20*
rs11184435	1	105657883	C	A	4.73E-06	0.0237765	*RP11-414B7.1*
rs139969191	15	27590371	C	T	4.74E-06	0.000139525	*GABRG3*
rs138554687	3	23113992	C	G	4.74E-06	0.00230906	*RPL24P7*
rs79058579	2	105819694	A	G	4.75E-06	0.000908501	*MRPS9*
20:45737915_CTTAAG_C	20	45737915	C	CTTAAG	4.81E-06	0.00650115	/
rs140898701	6	106814014	T	C	4.82E-06	3.82E-05	*ATG5*
rs534521017	4	107215642	CT	C	4.83E-06	0.0424774	/
rs151029329	15	37057383	G	A	4.88E-06	0.00109478	*C15orf41*
rs143047393	17	21801660	A	C	4.89E-06	0.000251656	*RP11-1109M24.15*
rs11950678	5	66654519	G	A	4.92E-06	2.82E-06	*CD180*
rs62073602	17	18057907	G	A	4.92E-06	0.000181584	*MYO15A*
rs12113414	7	146075680	G	A	4.93E-06	0.000149718	*CNTNAP2*
rs77091990	7	20264728	A	T	4.93E-06	0.0182866	*ITGB8*
rs117767075	10	70177061	G	A	4.93E-06	5.58E-09	*DNA2*
rs56084975	7	22031791	A	G	4.95E-06	0.0339603	*RAPGEF5*
rs4393145	1	237557302	T	G	4.97E-06	0.0221092	*RYR2*
rs62414809	6	63011050	C	G	4.99E-06	0.000114156	*KHDRBS2*
rs12291015	11	62487378	C	T	5.03E-06	7.14E-05	*HNRNPUL2*
rs138011046	16	3326104	A	G	5.03E-06	6.09E-05	*ZNF263*
rs114276351	1	224598645	T	C	5.04E-06	0.00806889	*WDR26*
rs181802249	17	39249357	C	T	5.04E-06	0.000473282	*KRTAP4-8*
rs6675	11	62505764	T	C	5.05E-06	7.37E-05	*TTC9C*
rs7104360	11	62503774	A	G	5.06E-06	7.38E-05	*TTC9C*
rs7949279	11	62502467	T	C	5.07E-06	7.39E-05	*TTC9C*
rs55894446	2	60617514	G	A	5.08E-06	2.19E-05	*BCL11A*
rs2576006	10	128330904	G	A	5.08E-06	1.40E-05	*C10orf90*
rs143917300	16	559760	G	C	5.08E-06	0.0430699	*RAB11FIP3*
rs11592576	10	1443180	T	A	5.08E-06	1.35E-05	*ADARB2*
rs7126912	11	62495239	C	A	5.09E-06	7.13E-05	*TTC9C*
rs6583116	1	105639616	C	G	5.10E-06	0.0211351	*RP11-414B7.1*
rs6583115	1	105639614	T	C	5.10E-06	0.0211375	*RP11-414B7.1*
rs11843073	13	95319843	A	T	5.11E-06	0.00104856	*SOX21*
rs77033618	5	86534291	C	T	5.12E-06	2.44E-05	*AC008394.1*
rs76972842	2	42493019	A	C	5.12E-06	0.0023505	*EML4*

rs201302260	13	103324232	CA	C	5.14E-06	0.000100831	*TPP2*
rs73203802	8	13591904	T	C	5.14E-06	0.0238574	*RP11-436P7.2*
rs16950006	13	95321913	G	C	5.14E-06	0.00104791	*SOX21*
rs71306533	3	150666046	T	C	5.15E-06	2.25E-06	*CLRN1*
rs56323441	11	62506792	G	T	5.15E-06	7.32E-05	*TTC9C*
rs72776415	16	6272462	A	G	5.16E-06	0.000765103	*RBFOX1*
rs76202562	12	5458610	C	T	5.20E-06	7.33E-05	*KCNA5*
rs6925557	6	106791650	C	G	5.21E-06	1.32E-05	*ATG5*
rs6900767	6	106791545	A	T	5.22E-06	1.33E-05	*ATG5*
rs10881254	1	105657725	G	A	5.24E-06	0.0237168	*RP11-414B7.1*
rs556361110	8	22069349	C	A	5.24E-06	0.000272379	*BMP1*
rs113777195	11	62513643	A	G	5.25E-06	7.05E-05	*ZBTB3*
rs112132759	7	146125166	A	G	5.26E-06	5.34E-05	*CNTNAP2*
rs12404997	1	105654091	C	T	5.26E-06	0.0237575	*RP11-414B7.1*
rs4847087	1	105647075	G	A	5.28E-06	0.0241414	*RP11-414B7.1*
rs34040681	10	1509044	T	G	5.28E-06	0.00236544	*ADARB2*
rs115027936	5	173944657	A	G	5.31E-06	0.00363744	*GAPDHP71*
rs202023115	5	102843177	GA	G	5.31E-06	0.0413828	*AC010423.1*
rs7936611	11	62492674	G	A	5.32E-06	8.14E-05	*HNRNPUL2*
rs113687404	3	76029264	C	T	5.32E-06	7.48E-05	*ROBO2*
rs76858681	17	64707856	T	C	5.33E-06	0.041488	*PRKCA*
rs72929452	11	62493867	C	T	5.34E-06	7.48E-05	*HNRNPUL2*
rs142421629	12	19023040	A	G	5.34E-06	2.04E-05	*CAPZA3*
rs4678876	3	36468079	T	G	5.34E-06	0.00166961	*STAC*
rs75094269	4	134210050	A	G	5.34E-06	0.00969628	*PCDH10*
rs144588202	1	239917071	C	T	5.35E-06	0.0143289	*CHRM3*
rs62072537	17	18216100	A	G	5.35E-06	0.045548	*TOP3A*
rs16961114	17	18215339	C	G	5.37E-06	0.0455277	*TOP3A*
rs116151125	1	53642460	T	C	5.37E-06	0.000200487	*CPT2*
rs6583117	1	105639679	G	A	5.37E-06	0.0223662	*RP11-414B7.1*
rs13156648	5	26903476	G	A	5.38E-06	0.0252148	*CDH9*
rs11184421	1	105639051	G	A	5.39E-06	0.0225189	*RP11-414B7.1*
rs4847092	1	105658443	G	A	5.39E-06	0.0235673	*RP11-414B7.1*
rs115847948	3	135113957	A	C	5.41E-06	0.000142238	*EPHB1*
rs73828877	3	36443186	G	C	5.42E-06	0.00164645	*STAC*
rs138389868	12	79976171	A	G	5.42E-06	0.0186691	*PAWR*
rs10513698	3	171425654	T	A	5.42E-06	0.00132172	*PLD1*
rs75793205	12	5478631	T	C	5.43E-06	3.29E-05	*KCNA5*
rs72712820	4	180023073	C	T	5.43E-06	0.0342635	*RP11-774G5.1*
rs62072515	17	18213435	G	T	5.45E-06	0.0459529	*TOP3A*
rs78469001	4	180024718	G	A	5.46E-06	0.0380058	*RP11-774G5.1*
rs76919197	6	112495348	G	T	5.47E-06	0.0184318	*LAMA4*
rs3734227	6	144510926	T	C	5.47E-06	0.00101476	*STX11*
rs752142503	11	62510922	C	CA	5.48E-06	7.17E-05	*TTC9C*
rs16950001	13	95316946	T	C	5.50E-06	0.0010451	*SOX21*
rs142047294	3	171422459	G	A	5.54E-06	0.00147148	*PLD1*
rs35636163	5	98037812	G	A	5.54E-06	0.0186456	*RGMB*
rs138042840	8	62768126	AT	A	5.54E-06	1.55E-06	*ASPH*
rs6583123	1	105661518	T	A	5.55E-06	0.0235058	*RP11-414B7.1*
rs72929471	11	62521475	C	T	5.57E-06	5.99E-05	*ZBTB3*
rs11184442	1	105662070	C	T	5.57E-06	0.0231664	*RP11-414B7.1*
rs141799119	16	6084305	T	C	5.58E-06	0.00545981	*RBFOX1*
12:48261192_TTG_T	12	48261192	T	TTG	5.59E-06	0.00148714	/
rs11985417	8	13593700	A	C	5.59E-06	0.0267263	*RP11-436P7.2*
rs57820954	16	74791827	C	G	5.60E-06	0.0372925	*FA2H*
rs11670372	19	9110090	A	G	5.61E-06	0.00238307	*MUC16*
rs111234733	7	146140807	G	T	5.61E-06	5.31E-05	*CNTNAP2*

rs7112576	11	62511420	A	G	5.62E-06	6.87E-05	*TTC9C*
rs73063829	3	36475321	A	T	5.62E-06	0.00127319	*STAC*
rs77648385	3	171413198	T	C	5.63E-06	0.00145532	*PLD1*
rs12636967	3	36434155	A	G	5.63E-06	0.00110003	*STAC*
rs77304365	17	69483912	G	A	5.63E-06	0.000708182	*LOC124685*
rs12631541	3	36434214	G	A	5.63E-06	0.00110092	*STAC*
rs4847093	1	105658506	G	C	5.67E-06	0.0251447	*RP11-414B7.1*
rs12039389	1	105659073	A	G	5.68E-06	0.0251291	*RP11-414B7.1*
rs77943717	3	36431468	A	G	5.68E-06	0.000980244	*STAC*
13:39919649_CAAAAAAAAAAAAAAAAA_C	13	39919649	C	CAAAAAAAAAAAAAAAAA	5.68E-06	2.83E-06	/
rs8081155	17	18191948	G	C	5.70E-06	0.0465343	*TOP3A*
rs62072511	17	18193157	A	G	5.70E-06	0.0465378	*TOP3A*
rs529628645	12	116172535	CA	C	5.70E-06	8.31E-05	*MED13L*
rs2885140	3	36454007	T	C	5.71E-06	0.00213202	*STAC*
rs145121459	10	25431803	A	T	5.72E-06	0.000406538	*GPR158*
rs3817992	17	18194011	A	C	5.73E-06	0.0465265	*TOP3A*
rs112389739	6	98216447	TTAAAG	T	5.74E-06	0.00458946	*MMS22L*
rs117992377	8	142982058	T	C	5.74E-06	2.26E-06	*AC138647.1*
rs12140941	1	4259253	C	A	5.76E-06	0.0411952	*AJAP1*
rs116683967	6	66125204	T	C	5.76E-06	4.54E-06	*EYS*
rs28363367	5	6741558	G	T	5.76E-06	1.96E-05	*PAPD7*
rs117475996	12	116149843	A	C	5.77E-06	3.17E-05	*MED13L*
rs1925	17	18177715	A	G	5.78E-06	0.0463377	*TOP3A*
rs140273457	4	134235478	C	T	5.80E-06	0.00335471	*PCDH10*
rs115987182	2	105949435	T	C	5.80E-06	0.000633337	*TGFBRAP1*
rs79782340	7	21301707	C	T	5.86E-06	0.0359604	*ASS1P11*
rs303736	9	14430737	A	G	5.87E-06	0.034068	*NFIB*
rs58303436	3	36433205	G	A	5.90E-06	0.00112161	*STAC*
rs12480992	20	40396754	T	C	5.91E-06	0.0350503	*CHD6*
rs4810338	20	40397965	T	C	5.94E-06	0.0351803	*CHD6*
rs760456	21	46329415	C	G	5.95E-06	0.000377306	*ITGB2*
rs62072509	17	18182393	A	G	5.97E-06	0.0484443	*TOP3A*
rs59998929	20	40397288	A	T	5.98E-06	0.0351564	*CHD6*
rs17034995	3	36431080	T	C	6.02E-06	0.00111111	*STAC*
rs62072507	17	18172335	A	G	6.04E-06	0.048345	*TOP3A*
rs117353270	18	49132256	A	C	6.04E-06	0.0474289	*RSL24D1P9*
rs140390274	8	126962162	G	A	6.04E-06	0.000124138	*SOD1P3*
rs11723971	4	180027296	A	G	6.05E-06	0.0438602	*RP11-774G5.1*
rs551152882	1	243224687	A	G	6.05E-06	8.50E-05	*RP11-261C10.7*
rs138424314	3	115147512	A	G	6.05E-06	5.67E-05	*GAP43*
rs2361038	3	36429578	C	T	6.07E-06	0.00110887	*STAC*
rs12112484	7	146156390	T	G	6.08E-06	5.27E-05	*CNTNAP2*
rs2361037	3	36429484	C	T	6.08E-06	0.00110924	*STAC*
rs78425039	2	42483860	G	C	6.09E-06	0.00201976	*EML4*
rs4678875	3	36459254	C	T	6.10E-06	0.00183252	*STAC*
rs145133878	1	17934347	A	C	6.11E-06	0.00129735	*ARHGEF10L*
rs6583124	1	105661612	A	G	6.12E-06	0.0237999	*RP11-414B7.1*
rs148492409	3	104931603	C	T	6.13E-06	0.0221239	*ALCAM*
rs151268864	20	17883163	C	T	6.13E-06	8.08E-06	*SNX5*
rs77501534	2	95824624	T	G	6.16E-06	2.98E-07	*ZNF514*
rs11184428	1	105650016	C	A	6.16E-06	0.0279557	*RP11-414B7.1*
rs6659453	1	105650012	C	G	6.16E-06	0.0279557	*RP11-414B7.1*
rs147013419	2	144974739	C	T	6.23E-06	0.0106463	*GTDC1*
rs118108633	11	87439442	A	G	6.23E-06	0.00120447	*RP11-665E10.5*
rs12128652	1	105642360	A	G	6.24E-06	0.0215483	*RP11-414B7.1*
rs12311827	12	19256833	T	A	6.24E-06	0.00791829	*RP11-282K24.2*

rs61954590	12	84241315	C	T	6.24E-06	8.69E-07	*RPL6P25*
rs950824	7	154528956	G	A	6.25E-06	0.0331468	*DPP6*
rs28452921	7	26539777	A	G	6.26E-06	0.00987674	*SNX10*
7:146088842_TTTGTTG_T	7	146088842	T	TTTGTTG	6.26E-06	7.50E-05	/
rs12652586	5	26913243	A	G	6.27E-06	0.0302445	*CDH9*
rs112600086	7	146095320	A	G	6.30E-06	6.07E-05	*CNTNAP2*
rs10748481	1	105660270	A	G	6.33E-06	0.0264369	*RP11-414B7.1*
17:18169000_CCCT_C	17	18169000	C	CCCT	6.33E-06	0.0492843	/
rs79275841	5	64429122	A	G	6.33E-06	5.22E-05	*ADAMTS6*
rs77383472	8	22069591	T	C	6.33E-06	0.000882518	*BMP1*
rs12128596	1	105642215	A	G	6.33E-06	0.0216584	*RP11-414B7.1*
rs189626704	1	93042999	T	A	6.34E-06	0.00153043	*EVI5*
rs117062745	12	97572424	T	C	6.34E-06	0.000136497	*NEDD1*
rs57846685	16	74796162	T	C	6.35E-06	0.0402492	*FA2H*
rs17034991	3	36426701	C	T	6.38E-06	0.00111598	*STAC*
1:105648179_AT_A	1	105648179	A	AT	6.41E-06	0.0261842	/
rs112883379	7	146101640	T	C	6.42E-06	6.28E-05	*CNTNAP2*
rs7411599	1	105646216	G	A	6.44E-06	0.0262165	*RP11-414B7.1*
rs7518450	1	105651502	A	G	6.44E-06	0.026218	*RP11-414B7.1*
rs10215407	7	26581775	C	T	6.45E-06	0.00496756	*KIAA0087*
rs6583121	1	105651783	C	T	6.54E-06	0.0264773	*RP11-414B7.1*
rs118092660	12	10049380	T	C	6.60E-06	5.63E-05	*CLEC2A*
rs34732465	11	72544977	AT	A	6.61E-06	0.00510486	*ATG16L2*
rs35314164	5	26899979	TA	T	6.66E-06	0.0167638	*CDH9*
rs3829248	11	62521663	T	C	6.66E-06	6.72E-05	*ZBTB3*
rs9438438	1	205884062	C	T	6.66E-06	0.00444891	*SLC26A9*
rs72929469	11	62521408	T	A	6.67E-06	6.80E-05	*ZBTB3*
rs147486502	17	49058892	T	A	6.67E-06	4.86E-10	*SPAG9*
rs10881253	1	105655635	C	T	6.72E-06	0.026006	*RP11-414B7.1*
rs6676532	1	105656276	G	A	6.72E-06	0.026006	*RP11-414B7.1*
rs34883409	5	66677647	TA	T	6.73E-06	2.05E-05	*CD180*
rs10881246	1	105646709	G	A	6.74E-06	0.0264304	*RP11-414B7.1*
rs11184429	1	105650116	T	A	6.74E-06	0.0260341	*RP11-414B7.1*
rs10881248	1	105646843	A	T	6.75E-06	0.0264376	*RP11-414B7.1*
rs7410808	1	105646629	T	C	6.75E-06	0.0264376	*RP11-414B7.1*
rs588057	5	154578752	T	C	6.75E-06	0.000226384	*CTD-2311A18.1*
rs113438017	7	146153929	T	C	6.80E-06	5.31E-05	*CNTNAP2*
rs115029880	1	76025899	C	T	6.83E-06	0.015814	*SLC44A5*
7:70702497_GA_G	7	70702497	G	GA	6.83E-06	0.0218837	/
rs6583122	1	105651943	C	A	6.87E-06	0.0262948	*RP11-414B7.1*
rs4847088	1	105647513	T	C	6.88E-06	0.0262948	*RP11-414B7.1*
rs144452076	11	68552827	A	G	6.88E-06	0.000198443	*CPT1A*
rs10881249	1	105648599	A	T	6.88E-06	0.026355	*RP11-414B7.1*
rs7415734	1	105652214	G	T	6.88E-06	0.0263409	*RP11-414B7.1*
rs11184434	1	105655745	T	C	6.89E-06	0.0263359	*RP11-414B7.1*
rs6964303	7	139009280	C	A	6.89E-06	0.0268395	*UBN2*
rs11184424	1	105647606	T	C	6.89E-06	0.0263922	*RP11-414B7.1*
rs11184425	1	105647638	C	A	6.89E-06	0.0263922	*RP11-414B7.1*
rs7517313	1	105648060	T	C	6.89E-06	0.0263922	*RP11-414B7.1*
4:122317716_AAAAG_A	4	122317716	A	AAAAG	6.89E-06	0.00402034	/
rs11184431	1	105652341	T	C	6.89E-06	0.026378	*RP11-414B7.1*
rs7412026	1	105656613	T	G	6.90E-06	0.0263798	*RP11-414B7.1*
rs4847032	1	105654555	T	A	6.90E-06	0.026375	*RP11-414B7.1*

rs4847091	1	105654850	T	C	6.90E-06	0.026375	*RP11-414B7.1*
rs11184439	1	105659144	T	C	6.90E-06	0.0258315	*RP11-414B7.1*
rs7412489	1	105656938	A	G	6.91E-06	0.0263882	*RP11-414B7.1*
rs62346385	5	26910332	T	C	6.92E-06	0.0307207	*CDH9*
rs10881250	1	105650446	T	C	6.92E-06	0.0263476	*RP11-414B7.1*
rs2826177	21	21699226	T	C	6.93E-06	0.00543555	*C1QBPP1*
rs11184426	1	105647717	G	C	6.94E-06	0.0262709	*RP11-414B7.1*
rs71174839	1	241898647	GC	G	6.94E-06	0.00241487	*WDR64*
rs191026992	13	70414224	G	A	7.01E-06	1.14E-05	*KLHL1*
rs10500688	11	7291369	G	T	7.02E-06	0.035571	*SYT9*
rs562358754	11	62504167	GA	G	7.07E-06	2.00E-05	*TTC9C*
rs2826180	21	21702869	G	T	7.08E-06	0.0109784	*C1QBPP1*
rs148661844	12	111761334	A	G	7.09E-06	7.58E-05	*CUX2*
rs146777635	12	116151851	G	T	7.09E-06	3.85E-05	*MED13L*
rs9909104	17	18248021	C	T	7.12E-06	0.0303484	*SHMT1*
rs76538932	4	134269562	G	C	7.14E-06	0.0032288	*PCDH10*
rs1297421	17	33120317	T	A	7.15E-06	2.16E-05	*CCT6B*
rs10158783	1	4263519	A	G	7.15E-06	0.0428611	*AJAP1*
rs4847089	1	105648916	C	T	7.15E-06	0.0260511	*RP11-414B7.1*
rs139135933	17	22006015	T	C	7.16E-06	0.000457743	*RP11-744K17.8*
rs139438716	17	22007042	G	A	7.21E-06	0.000457742	*RP11-744K17.8*
17:21979313_AG_A	17	21979313	A	AG	7.21E-06	0.000458014	/
rs765157457	17	21986388	T	TTGGCATATTTTGGGA	7.22E-06	0.000458383	/
17:21997356_GC_G	17	21997356	G	GC	7.22E-06	0.000458387	/
rs142742957	22	47766977	G	T	7.22E-06	3.48E-05	*TBC1D22A*
rs149207264	17	21982057	C	A	7.22E-06	0.000457995	*RP11-744K17.8*
rs143148863	17	21999976	G	T	7.23E-06	0.000458333	*RP11-744K17.8*
rs145680787	17	21995997	A	G	7.23E-06	0.00045834	*RP11-744K17.8*
rs1518051	17	21985976	A	G	7.23E-06	0.00045835	*RP11-744K17.8*
rs148534077	17	22001104	C	T	7.23E-06	0.000458343	*RP11-744K17.8*
rs1914613	17	21985742	G	A	7.23E-06	0.000458348	*RP11-744K17.8*
rs117710326	17	22000374	T	C	7.23E-06	0.000458332	*RP11-744K17.8*
rs4128942	1	105645726	A	G	7.23E-06	0.0266357	*RP11-414B7.1*
rs75378953	17	21989903	T	C	7.23E-06	0.000458656	*RP11-744K17.8*
rs117398458	17	21975033	A	T	7.23E-06	0.000459653	*RP11-744K17.8*
rs10881245	1	105643320	T	C	7.23E-06	0.0222772	*RP11-414B7.1*
rs151310017	17	21989917	A	G	7.24E-06	0.000460014	*RP11-744K17.8*
rs137993192	17	21994809	T	C	7.25E-06	0.000458249	*RP11-744K17.8*
rs144173214	12	116153585	A	C	7.26E-06	3.89E-05	*MED13L*
rs10785727	1	105650578	A	G	7.38E-06	0.0264144	*RP11-414B7.1*
rs115656147	1	25548475	A	G	7.39E-06	3.11E-07	*SYF2*
rs56722971	3	36424375	A	T	7.39E-06	0.00131129	*STAC*
rs71306532	3	150666037	A	C	7.41E-06	2.26E-06	*CLRN1*
rs115557126	7	146145255	A	T	7.43E-06	5.31E-05	*CNTNAP2*
rs113530263	15	27219750	A	G	7.44E-06	1.54E-05	*GABRG3*
rs1389538	3	19266335	G	A	7.44E-06	0.00297092	*KCNH8*
rs10222511	3	131384071	T	G	7.46E-06	0.0374742	*CPNE4*
rs78001008	4	27485467	A	C	7.46E-06	0.00380524	*STIM2*
rs10779949	2	62549445	A	C	7.47E-06	0.00249851	*B3GNT2*
rs138134962	4	47479028	T	C	7.52E-06	0.0129401	*ATP10D*
rs114664970	6	40127169	C	T	7.55E-06	9.59E-07	*LRFN2*
rs34336908	5	26909260	CT	C	7.56E-06	0.0195004	*CDH9*
rs8081343	17	18199150	A	G	7.57E-06	0.0487495	*TOP3A*
rs78186457	13	26498014	T	G	7.60E-06	0.000168379	*ATP8A2*

rs80009944	7	31043227	A	G	7.61E-06	0.0307555	*ADCYAP1R1*
11:62512381_TA_T	11	62512381	T	TA	7.62E-06	0.000609825	/
rs59750599	6	98229841	C	A	7.62E-06	0.00397958	*MMS22L*
rs146726391	8	6982284	G	C	7.63E-06	0.000129234	*RPS3AP30*
rs11184419	1	105638421	T	C	7.65E-06	0.0313797	*RP11-414B7.1*
rs62073650	17	18081577	A	G	7.65E-06	0.000581607	*MYO15A*
rs113139534	7	146147078	T	A	7.66E-06	5.30E-05	*CNTNAP2*
14:45802643_CTATTT_C	14	45802643	C	CTATTT	7.69E-06	2.00E-08	/
rs11041288	11	7283776	A	G	7.80E-06	0.0415639	*SYT9*
rs148561464	1	40603479	A	G	7.80E-06	0.00431182	*RP11-115D7.3*
rs145246068	10	126492394	T	C	7.81E-06	0.000113247	*ABRAXAS2*
rs8064657	17	55619829	G	A	7.82E-06	1.63E-07	*MSI2*
rs78301299	11	114965111	T	C	7.84E-06	0.000208301	*CADM1*
rs146011392	17	22007537	A	G	7.87E-06	0.00049004	*RP11-744K17.8*
rs144895463	17	22012457	T	C	7.88E-06	0.000489862	*RP11-744K17.8*
rs141251457	17	22012655	T	C	7.88E-06	0.000489853	*RP11-744K17.8*
rs143526496	17	22016206	T	A	7.88E-06	0.000489744	*RP11-846F4.1*
rs62541769	9	36209082	A	G	7.90E-06	0.030138	*CLTA*
rs2407647	21	21709136	G	A	7.96E-06	0.0185474	*C1QBPP1*
rs10839755	11	7288018	T	C	7.98E-06	0.0434712	*SYT9*
rs75497462	9	9202950	T	C	8.00E-06	0.000152885	*PTPRD*
rs10839754	11	7287818	A	G	8.01E-06	0.0437453	*SYT9*
rs6720190	2	107106572	T	G	8.04E-06	0.000134539	*CD8B2*
rs117343657	10	22039230	A	C	8.05E-06	0.009458	*MLLT10*
rs77590668	3	158116361	A	G	8.07E-06	0.00616839	*RSRC1*
rs141131066	3	158118893	C	T	8.08E-06	0.00619252	*RSRC1*
rs191738298	4	86778640	G	A	8.09E-06	8.56E-05	*ARHGAP24*
rs11029652	11	26646787	G	A	8.10E-06	0.0320052	*ANO3*
rs140263835	17	39239250	A	T	8.10E-06	0.000414378	*KRTAP4-7*
rs564284009	8	41648658	GAGA	G	8.11E-06	0.000838626	*ANK1*
rs117395564	11	104475775	A	G	8.12E-06	4.97E-05	*PDGFD*
rs56256051	11	12072943	G	A	8.12E-06	2.37E-06	*DKK3*
rs71640913	1	4257365	G	A	8.13E-06	0.0496778	*AJAP1*
rs542378728	9	39091561	C	T	8.15E-06	0.00202514	*CNTNAP3*
rs74653799	6	98226517	AG	A	8.17E-06	0.00418643	*MMS22L*
rs58726054	4	7560404	T	C	8.25E-06	0.00656238	*SORCS2*
rs116347716	7	146149451	G	A	8.31E-06	5.29E-05	*CNTNAP2*
rs17568237	5	75973445	T	C	8.32E-06	0.00926497	*IQGAP2*
rs111624519	7	146149802	A	T	8.34E-06	5.29E-05	*CNTNAP2*
rs770944021	22	48118271	G	GA	8.34E-06	0.0024452	*FAM19A5*
rs2826187	21	21709948	A	T	8.41E-06	0.0178267	*C1QBPP1*
rs114101428	2	62558951	T	C	8.42E-06	0.00786119	*B3GNT2*
rs116976804	7	144908420	G	A	8.42E-06	2.86E-06	*RPL7P59*
rs199568673	22	48112913	CA	C	8.43E-06	0.00145658	*FAM19A5*
rs138688067	3	132019987	A	G	8.49E-06	2.33E-05	*ACP3*
rs4877985	9	89124032	T	C	8.49E-06	0.000903378	*GAS1*
rs112067809	17	31702565	A	G	8.50E-06	1.26E-07	*ASIC2*
rs12576021	11	7287460	G	T	8.52E-06	0.0429881	*SYT9*
rs139037368	1	4246346	T	C	8.54E-06	0.0396106	*AJAP1*
rs2826185	21	21708991	A	G	8.54E-06	0.0202424	*C1QBPP1*
rs193227825	21	21660115	T	C	8.56E-06	0.00199695	*C1QBPP1*
rs202120653	19	55168250	ACATTTTCTT	A	8.60E-06	0.00105914	*AC009892.9*
rs10112791	8	13592517	C	G	8.61E-06	0.0367841	*RP11-436P7.2*
rs7498	17	18148485	A	G	8.63E-06	0.0426733	*FLII*
rs181235703	1	93358441	G	C	8.65E-06	0.00151932	*DIPK1A*

rs71428151	2	125290510	C	G	8.68E-06	3.89E-06	*CNTNAP5*
rs4860457	4	63051489	T	G	8.71E-06	0.0112383	*RP11-84A1.1*
rs79037957	16	72831111	C	G	8.78E-06	0.000498034	*ZFHX3*
rs116824245	2	8608228	A	G	8.82E-06	7.63E-07	*SNRPEP5*
rs12141123	1	239611507	G	A	8.84E-06	0.00389477	*CHRM3*
6:65986540_AG_A	6	65986540	A	AG	8.86E-06	8.07E-06	/
rs118131834	10	96504052	C	T	8.86E-06	0.0117445	*CYP2C19*
rs117605497	10	70106739	T	C	8.86E-06	8.03E-07	*RUFY2*
rs146296868	1	30406240	T	C	8.92E-06	5.86E-05	*RP3-357I16.1*
rs72741940	5	9187580	C	T	8.93E-06	0.0360162	*SEMA5A*
rs17746486	2	95722609	T	C	8.94E-06	2.32E-07	*MAL*
rs74670795	17	39071764	G	C	8.94E-06	0.000482467	*KRT23*
rs188341184	21	28781326	C	T	8.96E-06	1.58E-05	*RPL10P1*
rs117662606	8	95058960	A	C	8.97E-06	0.000354991	*CDH17*
rs117509604	6	128894951	G	A	8.98E-06	0.0357672	*PTPRK*
rs75077451	2	222575807	G	A	8.99E-06	0.000816895	*AC079834.1*
rs10137097	14	93096228	A	T	9.01E-06	0.00479553	*RIN3*
rs146468282	12	56768489	T	C	9.01E-06	0.00544062	*APOF*
rs115580702	2	25126911	G	C	9.04E-06	0.0195961	*ADCY3*
rs116401620	3	99168125	T	C	9.05E-06	0.00455951	*COL8A1*
rs141503063	4	111005626	C	A	9.06E-06	0.0342801	*ELOVL6*
rs2826188	21	21710072	T	G	9.07E-06	0.0179488	*C1QBPP1*
rs17883670	17	7591722	C	G	9.12E-06	0.0140105	*WRAP53*
rs74611811	5	55568117	C	T	9.13E-06	8.31E-05	*PSMC1P4*
rs113783935	6	20531169	A	G	9.13E-06	0.000458928	*CDKAL1*
rs7113137	11	7289078	C	T	9.17E-06	0.0343274	*SYT9*
rs9615205	22	48119371	G	T	9.23E-06	0.00225146	*FAM19A5*
rs61762827	4	149285042	A	G	9.25E-06	0.000941328	*NR3C2*
5:26916865_CAGAG_C	5	26916865	C	CAGAG	9.30E-06	0.0253323	/
rs62417467	6	43007097	G	C	9.35E-06	0.00262302	*CUL7*
rs17497628	5	26910902	G	C	9.44E-06	0.0293633	*CDH9*
rs62073607	17	18071338	G	A	9.45E-06	0.00181054	*MYO15A*
rs115526943	3	99166047	T	G	9.48E-06	0.00453139	*COL8A1*
rs7730501	5	26900472	C	T	9.49E-06	0.0270911	*CDH9*
rs9637769	5	52235853	T	C	9.57E-06	0.0453918	*ITGA1*
rs567959690	13	75152464	T	TTCTATCTATCTATCTA	9.57E-06	0.00147042	*AL355390.1*
rs62032667	16	55062315	T	C	9.62E-06	1.44E-05	*IRX6*
rs317984	5	66666739	G	A	9.64E-06	1.32E-05	*CD180*
rs139526467	19	29634123	A	G	9.64E-06	0.0406741	*UQCRFS1*
rs115243416	4	14010108	A	C	9.65E-06	0.0443517	*RP11-341G5.2*
rs76673557	20	17481097	T	C	9.66E-06	0.0103774	*BFSP1*
rs143372559	2	235193701	T	C	9.68E-06	0.0232874	*SPP2*
rs80198879	12	30157579	A	T	9.70E-06	4.13E-06	*TMTC1*
rs75318837	10	96415549	G	A	9.73E-06	0.0134788	*CYP2C18*
rs528391640	3	36427899	GAA	G	9.75E-06	0.00229086	/
14:102191282_CTA_C	14	102191282	C	CTA	9.75E-06	0.00893158	/
rs61916670	12	29215914	C	T	9.75E-06	0.0382401	*FAR2*
rs7734956	5	26900903	C	T	9.76E-06	0.0279294	*CDH9*
rs77397336	2	222587780	A	G	9.80E-06	0.000762878	*AC079834.1*
rs8073387	17	39391788	G	A	9.80E-06	0.000615809	*KRTAP9-8*
rs17862370	7	127029562	T	C	9.85E-06	9.70E-05	*ZNF800*
rs17497167	5	26896286	T	C	9.85E-06	0.0272539	*CDH9*
rs7524475	1	92982679	T	C	9.86E-06	0.00126878	*EVI5*

1:105636868_TG_T	1	105636868	T	TG	9.88E-06	0.0304069	/
rs6583114	1	105634963	C	T	9.90E-06	0.0209657	*RP11-414B7.1*
rs149001943	3	99174870	A	C	9.90E-06	0.00480692	*COL8A1*
rs76358560	15	71511297	G	A	9.92E-06	0.00593428	*THSD4*
rs1479685	5	26902078	C	T	9.92E-06	0.0285338	*CDH9*
rs72791324	16	75965325	T	A	9.95E-06	3.35E-05	*TERF2IP*
rs79063668	7	18994327	G	A	9.99E-06	0.00309456	*HDAC9*

Table S7. The PsRS-interacted SNPs for GAD score in discovery and replication cohort (Marked in red is significant).

SNP	CHR	Position	Eeference allele	Alternative allele	*P* discovery	*P* replication	Overlapped gene
rs146434301	18	24723498	TA	T	2.52E-10	9.04E-09	*CHST9*
rs140314791	4	68709501	T	C	1.06E-09	0.000101793	*TMPRSS11D*
rs2860008	4	68710963	C	T	1.06E-09	0.000100573	*TMPRSS11D*
rs10518045	4	68710842	C	T	1.06E-09	0.000100369	*TMPRSS11D*
rs116587563	4	68710465	A	C	1.06E-09	0.000100656	*TMPRSS11D*
rs17088693	4	68709690	C	T	1.06E-09	0.000101659	*TMPRSS11D*
rs114006170	4	68704816	T	C	1.14E-09	7.36E-05	*TMPRSS11D*
rs111606492	19	55105969	A	C	1.24E-09	6.54E-07	*LILRB1*
rs148060334	4	68705712	G	A	1.44E-09	0.00010652	*TMPRSS11D*
rs17576689	4	68704958	C	T	1.44E-09	0.000107208	*TMPRSS11D*
rs1545935	4	68700984	C	G	1.69E-09	0.000121113	*TMPRSS11D*
rs115427586	4	68681137	C	T	1.75E-09	0.000132068	*TMPRSS11D*
rs76632488	4	68684456	A	G	1.79E-09	0.000146241	*TMPRSS11D*
rs77927903	10	23161188	C	T	2.40E-09	0.00222043	*PIP4K2A*
rs184992408	18	41869044	G	T	2.65E-09	1.44E-07	*KRT8P5*
rs78422940	13	104942046	T	C	3.99E-09	4.94E-05	*RPL7P45*
rs78286508	6	153802852	G	T	7.07E-09	3.34E-06	*AL590867.1*
rs80221096	10	23188930	T	C	9.41E-09	0.00148762	*PIP4K2A*
4:68667012_CAAT_C	4	68667012	C	CAAT	1.49E-08	1.42E-05	/
rs147176127	18	32120008	C	G	1.59E-08	5.35E-07	*DTNA*
rs11638904	15	37309303	G	C	1.76E-08	0.00588768	*MEIS2*
rs10924842	1	246969261	C	G	2.26E-08	4.12E-05	*KIF28P*
rs10924841	1	246968607	C	T	2.34E-08	7.46E-05	*KIF28P*
rs4926469	1	246969804	T	G	3.12E-08	5.04E-05	*KIF28P*
rs59378072	2	8744978	T	C	3.23E-08	0.000220711	*SNRPEP5*
rs61852556	1	246970258	C	T	3.64E-08	5.61E-05	*KIF28P*
rs719764	6	152522373	A	G	3.89E-08	0.000308436	*SYNE1*
rs80024556	9	101172602	A	G	4.40E-08	0.000117894	*GABBR2*
rs80029890	7	54449520	T	G	4.41E-08	0.000423656	*SLC25A5P3*
rs4591276	19	55029367	C	G	4.54E-08	0.000717896	*AC008746.9*
rs77483076	6	152523736	C	G	7.86E-08	0.00226792	*SYNE1*
rs79630665	2	221238702	C	T	8.24E-08	0.0289031	*AC009310.1*
7:38775121_GTGTA_G	7	38775121	G	GTGTA	8.34E-08	0.0433953	/
rs115967737	1	220260674	A	G	1.05E-07	0.00787438	*BPNT1*
rs76696574	1	105668020	C	G	1.06E-07	0.000662636	*RP11-414B7.1*
rs183444862	7	120029186	G	A	1.07E-07	0.000139099	*KCND2*
rs142221374	3	153568239	G	A	1.14E-07	0.0233329	*C3orf79*
rs78006154	4	44367358	A	G	1.15E-07	0.0132134	*KCTD8*
rs147997219	19	49004917	T	C	1.22E-07	2.16E-06	*LMTK3*
rs73100617	7	32132611	G	C	1.22E-07	4.16E-05	*PDE1C*
rs11719004	3	100349	C	A	1.24E-07	0.00521654	*CHL1*
rs142882569	3	45338144	T	G	1.31E-07	0.0135181	*LARS2*
rs192339362	3	45332746	G	T	1.31E-07	0.0135186	*LARS2*
rs141360714	5	16239143	G	T	1.39E-07	0.0245929	*ZNF622*
rs139196868	1	209980595	C	T	1.66E-07	0.00430413	*IRF6*
rs115549955	3	5678469	A	G	1.80E-07	0.000176873	*EDEM1*
rs72647054	13	101504370	A	G	1.93E-07	2.28E-06	*NALCN*
rs17389618	1	209999401	G	A	1.96E-07	0.0075849	*DIEXF*
rs4926467	1	246968714	T	C	2.13E-07	0.000169522	*KIF28P*
rs111581301	16	79605807	C	G	2.20E-07	0.000680926	*MAF*
rs4926468	1	246968746	A	G	2.26E-07	0.000152563	*KIF28P*

rs188018003	7	131456777	T	C	2.37E-07	2.36E-05	*AC009518.2*
rs372567922	8	13564360	CA	C	2.38E-07	0.000453999	*RP11-436P7.1*
rs73702587	8	115472570	T	A	2.63E-07	1.18E-07	*RP11-393K19.1*
rs73702589	8	115475185	A	T	2.64E-07	1.20E-07	*RP11-393K19.1*
2:51912409_GTA_G	2	51912409	G	GTA	2.69E-07	6.72E-05	/
rs73702588	8	115472734	C	T	2.71E-07	1.25E-07	*RP11-393K19.1*
rs77973424	8	115473565	G	A	2.71E-07	1.25E-07	*RP11-393K19.1*
rs16885943	8	115499823	C	T	2.71E-07	1.26E-07	*RP11-393K19.1*
rs16885944	8	115499948	A	T	2.71E-07	1.26E-07	*RP11-393K19.1*
rs73702594	8	115483551	T	A	2.72E-07	1.25E-07	*RP11-393K19.1*
rs73702593	8	115483517	A	G	2.72E-07	1.25E-07	*RP11-393K19.1*
rs73702591	8	115483413	A	G	2.72E-07	1.25E-07	*RP11-393K19.1*
rs746524	8	115496875	T	C	2.82E-07	1.34E-07	*RP11-393K19.1*
rs73149812	22	17553526	C	T	2.84E-07	0.0045759	*IL17RA*
rs17653230	10	120201622	A	T	2.88E-07	0.00422966	*FAM204A*
rs56346559	8	115506657	C	T	2.94E-07	1.39E-07	*RP11-393K19.1*
rs77359095	6	153866784	C	G	2.99E-07	2.21E-05	*AL590867.1*
rs72746742	15	85827911	A	G	3.05E-07	0.00900568	*ADAMTS7P4*
rs16872719	7	21257691	A	G	3.27E-07	0.0380608	*ASS1P11*
rs77736207	8	115457533	G	A	3.27E-07	5.84E-08	*RP11-393K19.1*
rs73702585	8	115460709	A	G	3.31E-07	5.96E-08	*RP11-393K19.1*
rs59630764	8	115461018	A	G	3.31E-07	5.96E-08	*RP11-393K19.1*
rs60474673	8	115465762	C	A	3.32E-07	5.95E-08	*RP11-393K19.1*
rs75349344	8	115454607	T	G	3.33E-07	5.95E-08	*RP11-393K19.1*
rs62374568	5	132733367	T	C	3.39E-07	0.000269633	*FSTL4*
7:4190210_GT_G	7	4190210	G	GT	3.40E-07	0.0014428	/
rs117025112	11	967944	G	A	3.41E-07	0.021713	*AP2A2*
rs75123652	8	115459030	C	G	3.44E-07	5.96E-08	*RP11-393K19.1*
rs16885873	8	115467355	T	C	3.45E-07	1.00E-07	*RP11-393K19.1*
rs146468282	12	56768489	T	C	3.57E-07	0.00101217	*APOF*
rs78484020	3	193684005	A	G	3.73E-07	0.00165574	*RP11-528A4.3*
rs77767147	8	115528006	A	C	3.85E-07	1.88E-07	*RP11-393K19.1*
rs74803995	3	193677469	T	C	3.88E-07	0.00165759	*RP11-528A4.3*
rs73705321	8	115532533	C	A	4.07E-07	1.99E-07	*RP11-393K19.1*
rs189245179	2	84357713	T	C	4.23E-07	0.000235976	*FUNDC2P2*
rs114882422	1	188037391	A	T	4.60E-07	3.94E-07	*RP11-134C1.1*
rs118014449	16	6126384	C	T	4.61E-07	0.0373574	*RBFOX1*
rs76188550	22	34814862	A	G	4.61E-07	0.00109138	*LARGE*
rs141065459	8	115537595	G	A	4.80E-07	2.40E-07	*RP11-393K19.1*
rs568907752	1	188002743	C	G	4.83E-07	2.33E-07	*RP11-134C1.1*
rs141034234	3	197353587	C	T	5.20E-07	0.0464543	*AC024560.3*
rs724717	8	115538787	A	G	5.27E-07	2.68E-07	*RP11-393K19.1*
rs17354902	1	187954825	A	C	5.40E-07	7.67E-07	*RP11-134C1.1*
rs77458809	3	193672161	T	C	5.48E-07	0.00216756	*RP11-528A4.3*
rs76456149	8	115542652	T	G	5.55E-07	2.88E-07	*RP11-393K19.1*
rs76506384	7	54312950	T	G	5.67E-07	0.00523884	*SLC25A5P3*
rs113921735	12	101935985	T	G	5.77E-07	0.0203551	*SPIC*
rs73705323	8	115548212	T	C	6.02E-07	3.16E-07	*RP11-393K19.1*
rs71451165	11	29405492	T	C	6.13E-07	0.000211978	*RP11-460B17.1*
rs76063481	21	28728691	T	C	6.24E-07	2.16E-06	*GPX1P2*
rs77412674	8	115554739	T	C	6.26E-07	3.30E-07	*RP11-393K19.1*
rs553615290	11	46049642	AC	A	6.36E-07	0.000139642	*PHF21A*
rs185184464	10	25399461	C	G	6.48E-07	5.51E-06	*GPR158*
rs75820280	3	193676604	T	C	6.62E-07	0.00173851	*RP11-528A4.3*
rs12215234	6	49640046	G	A	6.64E-07	2.63E-05	*RHAG*
rs114336979	3	193674845	A	G	6.66E-07	0.00232473	*RP11-528A4.3*

4:108468503_ATT_A	4	108468503	A	ATT	6.73E-07	0.0346573	/
16:56985739_CTT_C	16	56985739	C	CTT	6.97E-07	0.000247513	/
rs73201271	4	2218685	G	A	7.09E-07	0.000105224	*POLN*
rs72866387	6	47831141	T	C	7.22E-07	2.90E-05	*OPN5*
rs182780958	3	31191252	G	A	7.32E-07	0.0328292	*GADL1*
rs118048058	8	136154777	C	G	7.40E-07	4.85E-07	*KHDRBS3*
rs146372107	8	136154729	A	C	7.40E-07	4.85E-07	*KHDRBS3*
rs56338040	4	108467222	T	C	7.41E-07	0.0244046	*PAPSS1*
rs191452721	3	31191251	T	C	7.42E-07	0.0327462	*GADL1*
rs149137169	8	53343488	A	C	7.45E-07	2.68E-06	*ST18*
rs71324488	21	47475825	T	C	7.86E-07	0.000150502	*COL6A1*
rs140641696	3	31210611	G	A	7.88E-07	0.0212091	*GADL1*
rs73705324	8	115566786	A	G	7.89E-07	4.37E-07	*RP11-393K19.1*
4:108467809_CA_C	4	108467809	C	CA	8.02E-07	0.0320653	/
rs73705325	8	115568873	T	G	8.05E-07	4.49E-07	*RP11-393K19.1*
rs73705326	8	115569591	G	A	8.05E-07	4.49E-07	*RP11-393K19.1*
rs6854956	4	112901719	A	G	8.12E-07	0.0237034	*RP11-255I10.1*
rs181518713	3	87071545	A	C	8.20E-07	0.00017019	*VGLL3*
rs117699451	21	28716047	T	A	8.42E-07	3.03E-06	*GPX1P2*
rs117223994	15	55002233	T	G	8.52E-07	0.00256556	*UNC13C*
rs529115038	8	115486745	GA	G	8.62E-07	3.69E-07	/
8:115477078_AT_A	8	115477078	A	AT	8.62E-07	3.66E-07	/
rs114021007	5	155373160	T	C	9.28E-07	0.0063275	*SGCD*
rs151336847	4	108453589	CA	C	9.49E-07	0.0431094	*PAPSS1*
rs115709053	1	37596458	T	G	9.50E-07	0.00853536	*GRIK3*
rs72671489	4	108455800	T	A	9.52E-07	0.0434715	*PAPSS1*
rs72671488	4	108455798	T	G	9.53E-07	0.0434769	*PAPSS1*
rs17037828	4	108453123	G	A	9.58E-07	0.043567	*PAPSS1*
rs72671487	4	108454875	A	G	9.58E-07	0.0435505	*PAPSS1*
rs60388823	4	108456918	G	A	9.59E-07	0.0435465	*PAPSS1*
rs17037832	4	108455721	T	A	9.59E-07	0.043548	*PAPSS1*
rs73837898	4	108457550	A	G	9.59E-07	0.0438824	*PAPSS1*
rs56837294	4	108456938	A	G	9.61E-07	0.0435526	*PAPSS1*
rs72682328	4	112901281	G	A	9.64E-07	0.0249584	*RP11-255I10.1*
rs201571046	8	115500163	TA	T	9.88E-07	4.07E-07	*RP11-393K19.1*
rs118190018	16	7319648	T	C	9.88E-07	0.000784992	*RBFOX1*
rs373301858	17	7862593	TA	T	1.00E-06	1.78E-06	/
rs188808988	15	36691902	A	C	1.00E-06	8.24E-08	*RP11-702M1.2*
rs75124487	4	108468550	C	A	1.07E-06	0.0288982	*PAPSS1*
rs777812851	1	246973506	A	AAAAGGATAG	1.11E-06	1.24E-05	*KIF28P*
rs62344144	5	165301	A	C	1.11E-06	0.000146004	*PLEKHG4B*
rs6671004	1	246972086	T	A	1.12E-06	3.52E-05	*KIF28P*
rs6674135	1	246972200	C	T	1.15E-06	3.77E-05	*KIF28P*
rs1745960	13	113730600	A	T	1.16E-06	0.0223001	*MCF2L*
rs12124993	1	186284321	C	A	1.20E-06	0.000968027	*TPR*
rs79481713	3	31237386	C	T	1.23E-06	0.0244613	*CNN2P6*
rs140457754	3	80497135	C	T	1.25E-06	0.00219471	*RP11-481N16.1*
rs191883076	6	100439576	C	T	1.26E-06	2.29E-05	*MCHR2*
rs79282006	9	14789979	C	A	1.29E-06	0.00657357	*FREM1*
rs141979416	21	28387891	C	G	1.31E-06	0.0433409	*ADAMTS5*
rs149469089	3	68327787	G	C	1.32E-06	2.38E-05	*FAM19A1*
rs72682321	4	112894500	G	C	1.34E-06	0.0259013	*RP11-255I10.1*
rs62331694	4	112896237	C	A	1.39E-06	0.0265303	*RP11-255I10.1*

rs180786175	8	115517871	A	G	1.39E-06	5.77E-07	*RP11-393K19.1*
rs186248171	8	115517875	C	T	1.39E-06	5.77E-07	*RP11-393K19.1*
rs17527759	4	112915030	C	G	1.47E-06	0.044395	*RP11-255I10.1*
rs78981156	19	55064611	A	G	1.48E-06	0.0021199	*AC009892.2*
rs548073365	8	115521897	A	G	1.49E-06	6.24E-07	*RP11-393K19.1*
rs73022033	3	4403260	T	C	1.49E-06	0.00438654	*SUMF1*
rs115989117	2	215190637	A	G	1.56E-06	1.85E-06	*SPAG16*
rs138618203	11	9597085	A	G	1.59E-06	0.000414019	*WEE1*
rs17574963	10	120208008	A	C	1.61E-06	0.0125535	*FAM204A*
rs73705330	8	115572386	C	G	1.61E-06	1.06E-06	*RP11-393K19.1*
rs117512829	11	102069135	C	A	1.62E-06	0.0445885	*YAP1*
rs117969745	18	64889618	C	A	1.64E-06	2.90E-05	*RPL31P9*
rs143896981	8	115023762	G	T	1.68E-06	1.91E-07	*RP11-67H2.1*
rs78460917	12	90726337	C	T	1.69E-06	4.31E-05	*CCER1*
rs150816374	3	130211691	T	G	1.70E-06	2.11E-05	*COL6A5*
rs138738731	3	164647731	G	A	1.71E-06	2.04E-06	*SI*
rs575051668	7	31426904	T	C	1.74E-06	0.00064115	*NEUROD6*
rs116847314	18	61057007	C	A	1.76E-06	0.0011972	*VPS4B*
rs28529767	1	206300551	T	C	1.77E-06	0.00648047	*CTSE*
rs145816499	3	124109466	C	T	1.80E-06	1.83E-06	*KALRN*
rs2714445	7	79878921	T	C	1.81E-06	0.000302076	*GNAI1*
rs9841038	3	15453986	T	G	1.81E-06	0.0143833	*METTL6*
rs72685482	8	142539257	T	C	1.82E-06	2.19E-05	*AC138647.1*
rs4926465	1	246968455	A	G	1.84E-06	0.000394128	*KIF28P*
rs4912164	1	57887992	T	C	1.84E-06	0.000338405	*DAB1*
rs544072956	5	156344228	A	T	1.88E-06	2.66E-05	*TIMD4*
rs201856464	15	56592908	G	A	1.92E-06	0.000660545	*TEX9*
rs17179857	3	87065282	T	C	1.92E-06	0.000869089	*VGLL3*
rs1228912	7	84127834	G	C	1.98E-06	0.0212167	*SEMA3A*
rs116845939	7	88493918	G	T	2.00E-06	1.87E-05	*ZNF804B*
rs79498666	6	100436719	C	G	2.02E-06	2.38E-05	*MCHR2*
rs7531476	1	246973530	T	A	2.06E-06	2.88E-05	*KIF28P*
rs371045168	20	8488417	GAAGAT	G	2.06E-06	0.0484166	/
rs12646388	4	112896102	G	T	2.08E-06	0.0343955	*RP11-255I10.1*
rs186937865	3	61585601	C	G	2.09E-06	0.0110187	*PTPRG*
rs117621835	8	79883010	A	G	2.10E-06	0.0429189	*IL7*
rs74652565	4	79702818	T	C	2.11E-06	3.00E-07	*BMP2K*
rs7531462	1	246973479	G	A	2.11E-06	1.75E-05	*KIF28P*
rs78354327	8	115574055	A	G	2.12E-06	1.49E-06	*RP11-393K19.1*
rs139424815	10	3109286	G	C	2.12E-06	0.00175217	*PFKP*
rs183917631	14	86791060	A	G	2.15E-06	1.42E-05	*FLRT2*
rs115073069	20	11069473	G	T	2.15E-06	0.0225578	*C20orf187*
rs36187401	4	132274569	A	G	2.16E-06	0.000492283	*RP11-758B24.1*
rs183074991	1	95617626	G	A	2.23E-06	4.55E-07	*TMEM56*
rs2727729	12	119730802	T	C	2.24E-06	0.0272744	*HSPB8*
rs386810407	19	52384077	CTTCCATTA	C	2.24E-06	0.0488628	*ZNF577*
rs77448671	16	18051633	T	C	2.33E-06	0.0245541	*RPL7P47*
rs75634755	20	11079395	T	C	2.34E-06	0.0238884	*C20orf187*
rs17867508	4	118513308	A	G	2.34E-06	0.00556917	*NDST3*
rs79158798	15	68287324	A	G	2.37E-06	7.55E-05	*PIAS1*
rs117702508	7	96505432	G	A	2.38E-06	0.0370397	*DLX6*
rs288794	13	68457521	T	G	2.41E-06	0.00173011	*NPM1P22*
rs9426273	1	246972266	T	C	2.43E-06	4.50E-05	*KIF28P*
rs79042120	7	130580396	C	T	2.45E-06	0.0190852	*MKLN1*
rs112121552	19	52385589	ACC	A	2.47E-06	0.0475311	*ZNF577*
rs145658929	1	246973875	A	AT	2.50E-06	2.44E-05	*KIF28P*
rs117929040	7	96514406	T	A	2.51E-06	0.038674	*DLX6*

rs35020972	3	15460692	TA	T	2.52E-06	0.0323429	*METTL6*
rs149179325	7	130557425	AGAGAG AAGAGA GAGATA AGAGAG	A	2.53E-06	0.0141583	*MKLN1*
rs3910751	2	174218857	G	A	2.59E-06	0.00111052	*AC092573.2*
rs11610071	12	111795257	C	A	2.62E-06	0.000303177	*FAM109A*
rs41281112	13	100518634	T	C	2.62E-06	0.000559024	*CLYBL*
rs35381071	4	68694828	T	TG	2.62E-06	0.00105586	*TMPRSS11D*
rs73705337	8	115574639	C	T	2.63E-06	1.95E-06	*RP11-393K19.1*
rs190389090	7	124083457	C	T	2.67E-06	0.00722445	*SSU72P8*
rs11584835	1	246973094	G	A	2.67E-06	2.80E-05	*KIF28P*
rs112318373	6	124221412	T	C	2.69E-06	0.000105163	*NKAIN2*
rs115788078	1	82901875	G	C	2.70E-06	0.0436472	*ADGRL2*
rs586407	11	119994765	A	G	2.75E-06	4.87E-05	*TRIM29*
rs61831886	10	1517146	T	C	2.78E-06	0.000868704	*ADARB2*
rs2005398	13	113732347	T	C	2.78E-06	0.0347182	*MCF2L*
rs288770	13	68454413	T	C	2.80E-06	0.00209107	*NPM1P22*
rs55948526	1	246973852	A	G	2.87E-06	2.95E-05	*KIF28P*
rs78947267	7	130555019	G	A	2.88E-06	0.0170972	*MKLN1*
rs7534538	1	246973700	C	T	2.88E-06	3.05E-05	*KIF28P*
rs75342732	7	130555355	C	T	2.88E-06	0.0170904	*MKLN1*
rs35913465	1	246972810	A	G	2.92E-06	3.41E-05	*KIF28P*
rs113846010	7	130553642	A	G	2.94E-06	0.0174271	*MKLN1*
rs143125011	19	1959827	C	A	2.96E-06	0.00115097	*CSNK1G2*
rs9877409	3	4372386	G	C	2.96E-06	0.000350935	*SUMF1*
rs188313541	15	68298092	T	A	2.98E-06	0.000135128	*PIAS1*
rs7518032	1	246973638	A	G	2.99E-06	2.93E-05	*KIF28P*
rs4926466	1	246968469	A	G	2.99E-06	0.000289223	*KIF28P*
rs17778661	19	52383288	G	C	2.99E-06	0.0495194	*ZNF577*
rs145121459	10	25431803	A	T	3.03E-06	0.000322188	*GPR158*
rs8102738	19	52388229	C	T	3.03E-06	0.0467015	*ZNF577*
rs77458785	19	52384295	G	T	3.03E-06	0.0493072	*ZNF577*
rs45437201	19	1952850	G	C	3.03E-06	0.00697524	*CSNK1G2*
rs59911578	19	52385597	C	G	3.04E-06	0.0494317	*ZNF577*
rs3007292	1	246974010	T	C	3.06E-06	2.97E-05	*KIF28P*
rs115084606	2	75552159	A	G	3.08E-06	0.000140524	*TACR1*
1:246974079_CG_C	1	246974079	C	CG	3.10E-06	3.02E-05	/
rs7515707	1	246973374	A	G	3.13E-06	3.16E-05	*KIF28P*
rs9597939	13	59718113	C	A	3.13E-06	0.000230259	*POLR3KP1*
rs288795	13	68457437	C	A	3.14E-06	0.00211849	*NPM1P22*
rs2280277	2	240656525	C	G	3.15E-06	0.0499533	*AC079612.1*
rs288767	13	68456682	C	T	3.16E-06	0.00199825	*NPM1P22*
rs74681460	7	130544376	T	C	3.16E-06	0.0195751	*MKLN1*
rs17574549	10	120195786	T	G	3.19E-06	0.0236217	*FAM204A*
rs62329971	4	112913171	G	T	3.20E-06	0.0496766	*RP11-255I10.1*
10:120195780_TA_T	10	120195780	T	TA	3.20E-06	0.0236009	/
rs574804956	4	108407751	CA	C	3.21E-06	0.0354429	*PAPSS1*
15:36754008_GT_G	15	36754008	G	GT	3.21E-06	0.000606651	/
rs2996558	1	246974187	C	T	3.24E-06	3.15E-05	*KIF28P*
rs75685360	12	43962152	T	C	3.26E-06	0.000662961	*RP11-73B8.2*
rs9686093	5	154445470	T	C	3.29E-06	3.31E-06	*KIF4B*
rs3007294	1	246974438	T	C	3.30E-06	3.15E-05	*KIF28P*
rs78117107	10	23287544	C	T	3.34E-06	0.00137597	*ARMC3*
rs7524529	1	246973158	T	C	3.35E-06	3.30E-05	*KIF28P*

rs111605732	10	131331939	G	A	3.35E-06	3.50E-10	*MGMT*
rs150163576	7	150055084	T	C	3.35E-06	0.0132766	*RARRES2*
rs142387919	7	150061074	T	C	3.40E-06	0.0117944	*REPIN1*
rs2996557	1	246974226	A	G	3.46E-06	3.03E-05	*KIF28P*
rs79525992	7	130555848	G	A	3.48E-06	0.0169657	*MKLN1*
rs774095435	9	18600715	T	TC	3.55E-06	0.036987	*ADAMTSL1*
rs188278	13	68453984	C	T	3.58E-06	0.002233	*NPM1P22*
rs185966	13	68454106	G	T	3.59E-06	0.00224278	*NPM1P22*
rs595963	6	20175722	A	G	3.59E-06	3.79E-05	*MBOAT1*
rs158299	13	68453900	T	C	3.61E-06	0.00221728	*NPM1P22*
rs71522592	8	131744663	A	G	3.62E-06	0.0207949	*ASAP1*
rs181320539	4	115090495	A	G	3.62E-06	0.00505178	*UGT8*
rs288769	13	68455565	T	C	3.64E-06	0.00218186	*NPM1P22*
rs142051800	7	88422978	C	A	3.65E-06	3.59E-05	*ZNF804B*
rs144631938	6	29476235	T	C	3.69E-06	0.00589618	*RPS17P1*
rs74146685	10	90032784	G	A	3.70E-06	0.0157932	*RNLS*
rs12983067	19	46913200	C	G	3.70E-06	0.000972848	*CCDC8*
rs2099647	19	1948089	T	C	3.73E-06	0.00564361	*CSNK1G2*
rs7520180	1	246965098	G	A	3.74E-06	0.000532954	*KIF28P*
rs3007293	1	246974222	A	T	3.75E-06	2.92E-05	*KIF28P*
rs78033352	7	50183456	C	T	3.77E-06	0.0460568	*C7orf72*
rs7531852	1	246973283	C	T	3.85E-06	3.28E-05	*KIF28P*
rs73705340	8	115577768	C	T	3.86E-06	3.02E-06	*RP11-393K19.1*
rs12882034	14	106519361	T	C	3.94E-06	0.000156342	*IGHV3-7*
rs7515514	1	246973156	A	G	4.02E-06	3.34E-05	*KIF28P*
rs7551504	1	246964771	T	G	4.05E-06	0.000529453	*KIF28P*
rs7186819	16	82706407	C	T	4.10E-06	5.88E-05	*CDH13*
rs62541805	9	36269958	T	C	4.11E-06	5.76E-06	*GNE*
rs144326611	9	36257538	T	C	4.12E-06	5.74E-06	*CLTA*
rs73021652	19	30739969	A	G	4.14E-06	3.46E-05	*ZNF536*
rs79004974	11	102052263	C	T	4.16E-06	0.00125896	*YAP1*
rs77215221	2	124004932	C	A	4.19E-06	0.0435129	*TSN*
rs113644415	18	64892371	T	C	4.23E-06	6.58E-05	*RPL31P9*
rs6072229	20	39629534	C	T	4.24E-06	2.03E-05	*TOP1*
rs142052678	13	65218913	A	G	4.24E-06	0.00126709	*LGMNP1*
rs55779577	19	1950112	A	T	4.25E-06	0.00691164	*CSNK1G2*
rs17603507	15	30234960	G	A	4.27E-06	0.000598968	*TJP1*
rs17177765	6	29482797	A	G	4.32E-06	0.00667183	*RPS17P1*
rs11661452	18	64893826	T	C	4.35E-06	6.67E-05	*RPL31P9*
rs145608950	1	115205487	T	G	4.35E-06	0.00175383	*DENND2C*
rs139499380	2	135087890	C	T	4.36E-06	0.000122537	*MGAT5*
rs6072230	20	39629538	G	A	4.38E-06	1.97E-05	*TOP1*
rs2996555	1	246974936	C	G	4.43E-06	3.81E-05	*KIF28P*
rs2996554	1	246975015	A	G	4.51E-06	3.91E-05	*KIF28P*
rs12127314	1	71472911	A	G	4.60E-06	0.00271486	*PTGER3*
rs72894982	11	43245078	T	C	4.65E-06	0.0368745	*HNRNPKP3*
rs138004333	6	69596677	C	T	4.66E-06	9.93E-05	*ADGRB3*
4:108382777_AAT_A	4	108382777	A	AAT	4.68E-06	0.0410785	/
rs184134422	7	109990427	T	C	4.69E-06	0.0110573	*IMMP2L*
rs71426282	2	123945178	A	C	4.70E-06	0.00551093	*TSN*
rs79782340	7	21301707	C	T	4.73E-06	0.00273808	*ASS1P11*
rs138663011	11	68106952	G	A	4.74E-06	1.04E-05	*LRP5*
rs7867475	9	18587204	A	C	4.77E-06	0.0053389	*ADAMTSL1*
rs73145433	12	100178737	A	G	4.78E-06	2.84E-06	*ANKS1B*
8:92664237_AG_A	8	92664237	A	AG	4.79E-06	0.01403	/
rs34694126	4	108411704	CAT	C	4.79E-06	0.0415733	/

rs17037683	4	108376221	T	A	4.80E-06	0.0418939	*PAPSS1*
rs72671419	4	108378789	G	A	4.80E-06	0.0418937	*PAPSS1*
rs7690326	4	108381672	G	A	4.80E-06	0.0418926	*PAPSS1*
rs7690333	4	108381681	G	A	4.80E-06	0.0418926	*PAPSS1*
rs28415019	4	108380916	T	G	4.80E-06	0.0418926	*PAPSS1*
rs1835346	6	161162290	G	A	4.82E-06	0.00843778	*PLG*
rs7867370	9	18587239	C	A	4.83E-06	0.00511207	*ADAMTSL1*
rs10631015	1	86339511	TAAAA	T	4.87E-06	0.000444197	*COL24A1*
rs6851675	4	108394219	C	A	4.88E-06	0.0426307	*PAPSS1*
rs6846065	4	108393857	G	A	4.89E-06	0.0425882	*PAPSS1*
rs114696892	3	77398283	A	G	4.92E-06	0.000657819	*ROBO2*
rs140028676	4	108375596	AT	A	4.95E-06	0.0429589	*PAPSS1*
rs150485043	4	108368293	A	G	4.95E-06	0.0429173	*PAPSS1*
rs17037768	4	108413114	G	T	4.95E-06	0.0429319	*PAPSS1*
rs17037762	4	108412908	C	A	4.95E-06	0.0429319	*PAPSS1*
rs149223968	4	108406552	G	T	4.95E-06	0.0429319	*PAPSS1*
rs182315905	4	108407203	T	C	4.95E-06	0.0429318	*PAPSS1*
rs72671459	4	108408109	A	G	4.95E-06	0.0429319	*PAPSS1*
rs28592707	4	108389952	C	T	4.96E-06	0.0429026	*PAPSS1*
rs17037711	4	108385102	T	A	4.96E-06	0.0429148	*PAPSS1*
18:5695961_TAATA_T	18	5695961	T	TAATA	4.98E-06	0.0153961	/
rs116683967	6	66125204	T	C	4.98E-06	8.37E-08	*EYS*
rs55814949	19	1953166	C	T	4.99E-06	0.00870628	*CSNK1G2*
4:108402730_AG_A	4	108402730	A	AG	5.10E-06	0.0432957	/
rs72669802	4	108357166	T	C	5.11E-06	0.0436385	*PAPSS1*
rs148661844	12	111761334	A	G	5.14E-06	0.000324024	*CUX2*
rs76406875	8	58821337	G	A	5.14E-06	0.0335607	*FAM110B*
rs149993972	8	79990982	T	C	5.17E-06	0.000934879	*IL7*
rs12459738	19	1932798	T	C	5.18E-06	0.00434098	*CSNK1G2*
rs75858231	7	130531946	A	G	5.20E-06	0.0340701	*AC016831.6*
rs58415489	19	1951647	T	C	5.24E-06	0.0137645	*CSNK1G2*
rs56256051	11	12072943	G	A	5.25E-06	5.22E-06	*DKK3*
rs288768	13	68456578	C	T	5.26E-06	0.00201879	*NPM1P22*
rs75008843	19	31786059	G	A	5.27E-06	4.96E-07	*TSHZ3*
rs191562832	17	57787529	T	G	5.30E-06	1.18E-05	*VMP1*
rs72671463	4	108410363	C	G	5.31E-06	0.0432559	*PAPSS1*
rs77656645	6	128232202	A	G	5.31E-06	4.63E-05	*THEMIS*
rs60440755	19	31782629	A	G	5.33E-06	4.96E-07	*TSHZ3*
rs202132012	4	108385870	TA	T	5.37E-06	0.0394987	*PAPSS1*
rs55965180	11	43262509	C	A	5.40E-06	0.0323695	*HNRNPKP3*
rs77728351	9	138656634	A	G	5.43E-06	0.00670918	*KCNT1*
rs77875911	19	31780846	G	C	5.44E-06	5.11E-07	*TSHZ3*
rs6065299	20	39640568	A	C	5.47E-06	5.64E-05	*TOP1*
rs73257882	20	39644855	T	C	5.49E-06	5.89E-05	*TOP1*
rs116205884	7	14825372	C	T	5.51E-06	7.47E-05	*DGKB*
rs374498896	9	82111472	G	T	5.65E-06	0.000453537	*TLE4*
rs1006325	20	39633603	T	C	5.65E-06	4.07E-05	*TOP1*
rs765689265	2	154420705	TTA	T	5.65E-06	0.0237067	/
1:157753232_GA_G	1	157753232	G	GA	5.66E-06	1.42E-07	/
rs1006383	20	39631671	G	A	5.67E-06	3.40E-05	*TOP1*
rs78639199	19	31779931	T	C	5.71E-06	5.40E-07	*TSHZ3*
rs72682332	4	112904652	T	C	5.74E-06	0.0377863	*RP11-255I10.1*
rs76919197	6	112495348	G	T	5.77E-06	0.0124582	*LAMA4*
rs6072241	20	39641473	A	G	5.78E-06	5.68E-05	*TOP1*
rs150622554	1	79199296	A	G	5.78E-06	1.27E-07	*RP4-641G12.4*

rs73257868	20	39634704	A	G	5.82E-06	3.57E-05	*TOP1*
rs114469298	1	95669553	T	G	5.85E-06	2.14E-06	*TMEM56-RWDD3*
rs2178804	7	130542828	A	G	5.85E-06	0.0200071	*MKLN1*
rs181750950	16	51326114	A	G	5.86E-06	4.28E-05	*HNRNPA1P48*
rs140918174	2	53085705	C	T	5.87E-06	3.41E-09	*AC010967.1*
rs112363273	20	39645285	C	T	5.87E-06	7.89E-05	*TOP1*
rs6442522	3	15440556	T	C	5.90E-06	0.0331808	*METTL6*
rs116918050	12	123610753	C	T	5.92E-06	0.00327818	*PITPNM2*
rs76843717	19	31798091	T	C	5.94E-06	3.40E-06	*TSHZ3*
rs6065298	20	39640060	A	G	5.96E-06	5.37E-05	*TOP1*
rs73257859	20	39630224	T	C	5.98E-06	3.10E-05	*TOP1*
18:5709882_TC_T	18	5709882	T	TC	6.01E-06	0.0373727	/
rs72671479	4	108435058	T	C	6.03E-06	0.0409166	*PAPSS1*
rs17037815	4	108444113	A	C	6.06E-06	0.0428984	*PAPSS1*
rs72671476	4	108430206	A	G	6.06E-06	0.0428547	*PAPSS1*
rs17037782	4	108421171	C	T	6.07E-06	0.0429079	*PAPSS1*
rs17037826	4	108449838	C	A	6.08E-06	0.0426746	*PAPSS1*
rs72671472	4	108426827	A	G	6.08E-06	0.0429434	*PAPSS1*
rs367692292	4	108448778	T	C	6.08E-06	0.0428897	*PAPSS1*
rs4276357	4	108446727	A	G	6.10E-06	0.0429225	*PAPSS1*
rs72671484	4	108442738	A	G	6.10E-06	0.042924	*PAPSS1*
rs148600869	4	108427635	A	G	6.10E-06	0.0429215	*PAPSS1*
rs17037810	4	108440616	G	A	6.10E-06	0.0429242	*PAPSS1*
rs199937856	4	108432358	CA	C	6.11E-06	0.0429244	*PAPSS1*
rs199857577	9	26001632	CT	C	6.11E-06	0.000187319	*CAAP1*
rs10488874	4	108420553	G	C	6.11E-06	0.0430312	*PAPSS1*
rs149531636	4	108425115	G	A	6.12E-06	0.0430066	*PAPSS1*
rs11944572	4	108435303	T	C	6.12E-06	0.042875	*PAPSS1*
rs73257865	20	39633163	C	T	6.17E-06	4.66E-05	*TOP1*
rs17037788	4	108422651	C	T	6.18E-06	0.042898	*PAPSS1*
rs2383080	9	18586678	G	A	6.20E-06	0.00587471	*ADAMTSL1*
rs6072223	20	39620847	A	T	6.21E-06	2.39E-05	*TOP1*
rs11532901	9	18589279	G	A	6.31E-06	0.00516579	*ADAMTSL1*
rs6065296	20	39629935	A	G	6.35E-06	3.12E-05	*TOP1*
rs141687852	8	58819162	A	G	6.37E-06	0.0337985	*FAM110B*
rs151197979	1	245366473	C	T	6.37E-06	3.06E-05	*KIF26B*
rs567959690	13	75152464	T	TTCTATCTATCTATCTA	6.37E-06	0.00182757	*AL355390.1*
rs73064790	7	14822956	A	T	6.40E-06	7.56E-05	*DGKB*
rs12782764	10	1509658	A	G	6.41E-06	0.00219855	*ADARB2*
rs150382208	15	25416332	A	C	6.44E-06	0.0136607	*TMEM261P1*
rs6072233	20	39636872	G	C	6.45E-06	6.02E-05	*TOP1*
rs6072242	20	39641948	T	C	6.48E-06	6.16E-05	*TOP1*
18:5708886_TA_T	18	5708886	T	TA	6.49E-06	0.0381117	/
rs72671478	4	108433745	G	A	6.57E-06	0.0431617	*PAPSS1*
rs574823708	2	38369441	G	T	6.57E-06	0.0093273	*CYP1B1*
rs184514320	2	85701985	C	A	6.61E-06	0.000194042	*SH2D6*
rs17313391	8	131691963	T	C	6.72E-06	0.0446692	*ASAP1*
rs113105757	4	188335074	A	T	6.72E-06	0.00229494	*MRPS36P2*
rs58481964	18	5708214	A	G	6.73E-06	0.0232618	*EPB41L3*
rs41525951	4	68669704	C	T	6.74E-06	0.0001025	*TMPRSS11D*
rs116731467	3	183993361	A	G	6.79E-06	0.0127079	*EIF2B5*
rs78856971	4	68672846	G	T	6.81E-06	0.000107251	*TMPRSS11D*
rs188341184	21	28781326	C	T	6.93E-06	1.88E-05	*RPL10P1*
rs1576897	9	18590952	T	C	6.94E-06	0.00487921	*ADAMTSL1*

rs117442926	7	95414630	C	G	7.03E-06	0.00920044	*DYNC1I1*
rs181116195	3	124097352	A	G	7.04E-06	9.83E-07	*KALRN*
14:48601996_GT_G	14	48601996	G	GT	7.05E-06	0.0498665	/
rs115716922	14	48601998	G	T	7.05E-06	0.0498665	*RP11-10A2.1*
rs117752475	16	69122402	A	G	7.09E-06	0.0038156	*TANGO6*
rs4624546	3	15437073	C	T	7.11E-06	0.0405021	*METTL6*
rs113894469	7	130547967	G	A	7.16E-06	0.0185609	*MKLN1*
rs1576898	9	18590804	C	T	7.17E-06	0.00475803	*ADAMTSL1*
rs72874479	11	28015568	T	C	7.19E-06	0.0366406	*KIF18A*
rs13096970	3	15439902	C	T	7.21E-06	0.041168	*METTL6*
rs68122973	18	5699240	T	C	7.22E-06	0.0234662	*EPB41L3*
rs2121545	7	130545517	T	G	7.25E-06	0.0192884	*MKLN1*
rs34203236	10	1509191	A	G	7.25E-06	0.00234776	*ADARB2*
rs7196182	16	77841325	G	C	7.26E-06	0.0011884	*VAT1L*
rs12608096	18	5709890	C	T	7.29E-06	0.02401	*EPB41L3*
rs111614646	7	130545183	C	T	7.29E-06	0.019394	*MKLN1*
rs3909916	16	77854848	G	A	7.29E-06	0.00179888	*VAT1L*
rs12956245	18	5698267	G	T	7.33E-06	0.0235201	*EPB41L3*
rs6072232	20	39631197	C	A	7.35E-06	4.22E-05	*TOP1*
rs12605334	18	5697101	T	G	7.43E-06	0.0236251	*EPB41L3*
rs12437668	15	72094454	C	A	7.50E-06	0.000115761	*MYO9A*
rs12604811	18	5696608	T	G	7.52E-06	0.0236861	*EPB41L3*
rs62155760	2	84407782	G	C	7.55E-06	8.62E-06	*FUNDC2P2*
rs72905696	11	44612648	T	C	7.56E-06	0.000302158	*CD82*
rs6762167	3	15445037	T	A	7.57E-06	0.0379298	*METTL6*
rs9855902	3	27727219	T	C	7.58E-06	0.0215725	*AC098614.2*
rs77393301	7	95404500	A	G	7.58E-06	0.00858374	*DYNC1I1*
rs12899374	15	36753040	C	A	7.59E-06	0.00116177	*RP11-702M1.2*
rs2219251	3	15429168	A	G	7.59E-06	0.0421856	*METTL6*
rs11918807	3	15446277	T	C	7.61E-06	0.0379828	*METTL6*
rs73372335	10	125922227	A	G	7.62E-06	0.0117411	*OAT*
rs79924254	3	27729357	T	A	7.62E-06	0.0217215	*AC098614.2*
rs79707181	7	130540256	G	A	7.64E-06	0.0201685	*MKLN1*
rs4372704	16	77849042	G	T	7.67E-06	0.00158474	*VAT1L*
rs142860985	4	165986827	T	C	7.68E-06	0.0482662	*TMEM192*
rs76623227	3	27731660	G	A	7.70E-06	0.0218696	*AC098614.2*
rs28420508	3	27724500	G	A	7.71E-06	0.0218187	*AC098614.2*
rs74446329	17	68876378	A	G	7.78E-06	0.01601	*RP11-1058G23.1*
rs116471345	3	27741868	T	C	7.79E-06	0.0225435	*AC098614.3*
rs72794901	5	139214934	G	C	7.79E-06	0.000427864	*PSD2*
rs76079178	3	27738430	A	G	7.80E-06	0.02232	*AC098614.2*
rs116375996	3	27739252	A	T	7.83E-06	0.0223952	*AC098614.2*
rs115944578	3	27734566	A	G	7.85E-06	0.0220964	*AC098614.2*
rs71453327	12	42371942	A	G	7.85E-06	0.0028036	*RP11-2A1.1*
rs17571316	15	30173956	T	C	7.88E-06	0.00136367	*TJP1*
rs17576726	2	130124874	A	G	7.91E-06	1.14E-06	*ISCA1P6*
10:120178512_GCC_G	10	120178512	G	GCC	7.97E-06	0.0443295	/
rs191510703	3	27745770	T	A	7.99E-06	0.0229251	*AC098614.3*
rs12934135	16	82820866	T	C	8.09E-06	0.025263	*CDH13*
rs146398630	11	67997388	T	C	8.10E-06	0.00344545	*SUV420H1*
rs141641643	6	6315590	T	C	8.26E-06	0.0326527	*F13A1*
18:5698307_AT_A	18	5698307	A	AT	8.29E-06	0.0344667	/
rs560287592	4	79930658	G	C	8.30E-06	3.84E-07	*NAA11*
rs1377723	15	36762580	G	A	8.32E-06	0.00114899	*RP11-702M1.2*
rs146293670	1	220268018	A	G	8.37E-06	0.0126806	*IARS2*

rs28588099	3	27751264	T	C	8.38E-06	0.0239693	*EOMES*
rs80081239	20	2848083	A	G	8.44E-06	0.00457005	*PTPRA*
8:115482375_ATAGT_A	8	115482375	A	ATAGT	8.46E-06	8.17E-05	/
rs7179674	15	36756990	G	A	8.47E-06	0.00117574	*RP11-702M1.2*
rs75819398	4	127643768	C	T	8.52E-06	0.00154475	*RBM48P1*
rs9847430	3	15449640	T	G	8.52E-06	0.0391651	*METTL6*
rs182562905	8	3752943	C	T	8.60E-06	1.65E-05	*CSMD1*
rs533829772	5	176096	T	C	8.62E-06	0.0184653	*PLEKHG4B*
rs13075694	3	15450400	T	C	8.66E-06	0.0398792	*METTL6*
rs13075773	3	15450569	T	A	8.68E-06	0.0398921	*METTL6*
rs72990900	2	211082539	A	G	8.69E-06	0.00108719	*ACADL*
rs6792128	3	15458857	G	A	8.70E-06	0.0405358	*METTL6*
rs3861261	16	77854705	G	C	8.75E-06	0.00271466	*VAT1L*
rs80072402	18	6660730	G	A	8.76E-06	0.000141574	*ARHGAP28*
rs117618378	7	130538887	C	G	8.92E-06	0.0168143	*MKLN1*
rs116818000	3	27753310	G	A	8.92E-06	0.0246766	*EOMES*
rs75404987	8	80026287	A	G	8.92E-06	0.000535667	*IL7*
rs77082285	19	52387583	A	G	8.95E-06	0.0491307	*ZNF577*
rs75486749	20	39966771	T	C	8.98E-06	0.00290765	*LPIN3*
rs1202201	6	20162878	A	G	9.03E-06	0.0174228	*MBOAT1*
rs79860681	20	39964461	A	G	9.14E-06	0.00281089	*RP3-450M14.1*
rs72841922	2	84338670	G	T	9.15E-06	0.00011279	*FUNDC2P2*
rs10204812	2	236410779	G	A	9.25E-06	0.0169784	*AGAP1*
rs6065337	20	39966060	A	C	9.31E-06	0.00286039	*LPIN3*
rs6072343	20	39968188	A	G	9.32E-06	0.00283734	*LPIN3*
rs56160161	3	112572246	T	C	9.42E-06	1.84E-05	*CD200R1*
rs113331764	16	77855470	G	A	9.43E-06	0.00279707	*VAT1L*
rs34040681	10	1509044	T	G	9.48E-06	0.00373332	*ADARB2*
rs114250585	4	68667630	A	G	9.49E-06	7.17E-05	*TMPRSS11D*
rs60108251	3	127176185	T	G	9.65E-06	5.73E-05	*TPRA1*
rs72794900	5	139212839	T	C	9.66E-06	0.000454289	*PSD2*
rs538443508	11	8727602	C	G	9.71E-06	9.10E-05	*DENND2B*
rs71309890	3	2401368	A	C	9.80E-06	0.00116016	*CNTN4*
rs9587342	13	107936790	A	G	9.89E-06	0.00939356	*FAM155A*
rs74431945	4	79448289	C	T	9.97E-06	6.47E-06	*FRAS1*

Table S8. The PsRS-interacted SNPs for self-reported depression in discovery and replication

SNP	CHR	Position	Eeference allele	Alternative allele	*P* discovery	*P* replication	Overlapped gene
rs13270264	8	15186872	A	G	4.15E-07	0.0495191	*RP11-782K4.1*
rs112858627	6	21805878	C	T	7.25E-07	0.0465774	*RP11-524C21.1*
rs59097328	6	21806250	A	G	7.43E-07	0.0432458	*RP11-524C21.1*
rs759904442	6	21800562	T	TAGTTCAT AAA	9.32E-07	0.0329306	/
6:21799264_ TA_T	6	21799264	T	TA	1.04E-06	0.0314449	/
rs7738357	6	21797634	T	C	1.12E-06	0.0346179	*RP11-524C21.1*
rs34532408	6	21807304	A	T	1.18E-06	0.0425854	*RP11-524C21.1*
rs7738472	6	21797544	T	G	1.57E-06	0.0359061	*RP11-524C21.1*
rs6933786	6	21799200	C	T	1.72E-06	0.0359753	*RP11-524C21.1*
6:21794731_ AAT_A	6	21794731	A	AAT	1.72E-06	0.0345461	/
rs9358459	6	21796788	C	G	1.76E-06	0.0374412	*RP11-524C21.1*
rs9358458	6	21796608	A	C	1.93E-06	0.0467082	*RP11-524C21.1*
rs9847419	3	61785125	G	A	2.07E-06	0.0133945	*PTPRG*
rs6935843	6	21796294	T	A	2.29E-06	0.0367143	*RP11-524C21.1*
rs796820303	6	21793906	G	GT	2.39E-06	0.0410176	/
rs1819685	13	63908772	G	A	2.42E-06	0.0011219	*OR7E156P*
rs79164741	13	63911387	A	T	2.43E-06	0.0010697	*OR7E156P*
rs115554083	13	63911397	T	C	2.43E-06	0.0010784	*OR7E156P*
rs4482153	13	63906500	G	A	2.46E-06	0.0011536	*OR7E156P*
rs116429100	13	63905839	T	C	2.47E-06	0.0011685	*OR7E156P*
rs9368349	6	21796583	C	A	2.85E-06	0.0443512	*RP11-524C21.1*
rs117966702	13	63910539	A	G	2.91E-06	0.0009499	*OR7E156P*
rs2181361	6	21793962	C	T	3.43E-06	0.039558	*RP11-524C21.1*
rs2181360	6	21793478	C	T	3.62E-06	0.0360652	*RP11-524C21.1*
13:63910097 _AT_A	13	63910097	A	AT	4.71E-06	0.001187	/
rs13212386	6	21795567	G	A	5.94E-06	0.0308483	*RP11-524C21.1*
rs144588845	11	4330769	G	C	6.60E-06	0.0020052	*SSU72P4*
6:21752673_ AT_A	6	21752673	A	AT	8.69E-06	0.002881	/
rs12002153	9	89300815	A	G	8.87E-06	0.0163041	*GAS1*
rs142183465	6	21781403	TTGTGT G	T	9.03E-06	0.0200467	*RP11-524C21.1*

Table S9. The PsRS-interacted SNPs for self-reported anxiety in discovery and replication cohort.

SNP	CHR	Position	Eeference allele	Alternative allele	*P* discovery	*P* replication	Overlapped gene
rs199600376	3	141387404	TTTTG	T	2.81E-08	0.0219056	RASA2
rs62484606	8	4629117	C	G	1.29E-07	0.0106378	CSMD1
rs111635254	3	1355796	C	A	1.38E-07	0.00910829	CNTN6
rs1544975	9	78076173	A	T	5.46E-07	0.0238249	RP11-360J12.1
rs4803133	19	53974402	G	C	5.58E-07	0.00133277	ZNF813
rs1359805	9	78080621	C	G	9.17E-07	0.0209027	RP11-360J12.1
rs11780422	8	139916909	G	A	9.42E-07	0.0101858	COL22A1
rs7038910	9	78078806	C	G	9.72E-07	0.0235201	RP11-360J12.1
rs4744749	9	78084484	C	G	9.78E-07	0.0204204	RP11-360J12.1
rs4745404	9	78072761	C	T	1.00E-06	0.0384841	RP11-360J12.1
rs7824825	8	139916826	G	A	1.01E-06	0.00763952	COL22A1
rs7828583	8	139916824	A	T	1.03E-06	0.004864	COL22A1
rs7828568	8	139916797	C	T	1.07E-06	0.00722421	COL22A1
9:78080525_AT_	9	78080525	A	AT	1.19E-06	0.0178178	/
rs1359806	9	78080515	G	A	1.21E-06	0.0237036	RP11-360J12.1
rs1123758	8	139889659	G	A	1.96E-06	0.0315975	COL22A1
rs553504	9	78081291	T	C	2.24E-06	0.00494627	RP11-360J12.1
rs2294845	1	210553523	A	C	2.75E-06	0.0103136	HHAT
rs524314	9	78089799	A	C	2.83E-06	0.00571424	RP11-360J12.1
rs113901498	19	53974835	T	G	3.55E-06	0.00162366	ZNF813
rs524346	9	78089813	G	A	3.68E-06	0.00673008	RP11-360J12.1
rs10124225	9	78069874	T	G	3.70E-06	0.0124595	RP11-360J12.1
rs11144445	9	78110424	A	C	4.09E-06	0.00484006	RP11-360J12.1
8:139916850_AT_A	8	139916850	A	AT	4.33E-06	0.00795844	/
9:78108497_AAACAATGCTGCAG_A	9	78108497	A	AAACAATGCTGCAG	4.56E-06	0.00578749	/
rs7866499	9	78108533	T	C	4.86E-06	0.00650209	RP11-360J12.1
rs752603387	9	94203050	T	TGCTTAGGA	5.03E-06	0.00500318	/
rs7862507	9	78108367	A	G	5.57E-06	0.0061998	RP11-360J12.1
rs1409933	9	78110916	C	T	5.57E-06	0.00623897	RP11-360J12.1
rs1409932	9	78110868	C	T	5.57E-06	0.00624269	RP11-360J12.1
rs1581881	9	78110317	C	G	5.57E-06	0.00624279	RP11-360J12.1
rs1409931	9	78110775	A	G	5.59E-06	0.0062445	RP11-360J12.1
rs7032347	9	78109702	C	T	5.60E-06	0.006247	RP11-360J12.1
rs1581880	9	78110159	G	T	5.65E-06	0.00626044	RP11-360J12.1
rs1854134	9	78109029	G	A	5.76E-06	0.00631515	RP11-360J12.1
rs1327369	9	78108869	G	A	5.76E-06	0.00631515	RP11-360J12.1
rs1854133	9	78108811	C	T	5.94E-06	0.0066043	RP11-360J12.1
rs528127	9	78111430	G	A	6.27E-06	0.00578447	RP11-360J12.1
rs3094188	6	31142245	A	C	6.33E-06	0.00322051	POU5F1
rs140124660	1	45418936	GA	G	6.73E-06	7.32E-06	EIF2B3
rs12424302	12	94881159	C	T	7.38E-06	0.0496239	CEP83
rs75586365	16	30996363	T	C	7.46E-06	0.00107468	SETD1A
rs777711382	5	96375135	A	AT	7.63E-06	0.0124123	LNPEP
rs13161540	5	96375671	G	A	8.00E-06	0.0148619	LNPEP
rs76611117	3	186738426	A	C	8.00E-06	0.0153969	ST6GAL1
rs13165925	5	96375851	C	T	8.09E-06	0.0129172	LNPEP
rs536195237	4	70816621	CA	C	8.12E-06	0.00389303	/
rs80123179	5	8916500	AAAG	A	8.22E-06	0.0144316	SEMA5A
rs7706943	5	96376766	A	T	8.42E-06	0.0145159	LNPEP
rs3846108	3	10379337	A	G	8.86E-06	0.0106297	ATP2B2
rs115944865	4	138117192	T	C	9.64E-06	0.000309524	TERF1P3

Table S10. Functional analysis results for PHQ score candidate genes.

**Category**	**GeneSet**	***P***	**Genes**
GO bp	GOBP ROUNDABOUT SIGNALING	1.37E-05	ROBO1:SLIT2
GO bp	GOBP VENTRICULAR SEPTUM MORPHOGENESIS	1.83E-05	PROX1:ROBO1:SLIT2
GO bp	GOBP CEREBRAL CORTEX TANGENTIAL MIGRATION	2.06E-05	ROBO1:SLIT2
GO bp	GOBP NEGATIVE REGULATION OF CHEMOKINE MEDIATED SIGNALING PATHWAY	2.88E-05	ROBO1:SLIT2
GO bp	GOBP OLFACTORY BULB INTERNEURON DEVELOPMENT	3.83E-05	ROBO1:SLIT2
GO bp	GOBP TANGENTIAL MIGRATION FROM THE SUBVENTRICULAR ZONE TO THE OLFACTORY BULB	3.83E-05	ROBO1:SLIT2
GWAScatalog	Adult body size	1.27E-06	ROBO1:GBE1:XXYLT1:LCORL:SLIT2:PRR16:CDH7
Reactome	REACTOME INACTIVATION OF CDC42 AND RAC1	2.88E-05	ROBO1:SLIT2
Reactome	REACTOME ROLE OF ABL IN ROBO SLIT SIGNALING	3.83E-05	ROBO1:SLIT2
Reactome	REACTOME REGULATION OF COMMISSURAL AXON PATHFINDING BY SLIT AND ROBO	6.15E-05	ROBO1:SLIT2
Reactome	REACTOME ACTIVATION OF RAC1	0.000106	ROBO1:SLIT2

Table S11. Multi-omics-based validation for top candidate genes of mental phenotypes.

Phenotype	Candidate Gene	Function confirmation		
		**Gene level(GWAS catalog)**	**Transcript level(TWAS Atlas)**	**Protein level(UniPort)**
**PHQ score**	*CD69*	Drinks per week	Lymphocyte Count	
	LCORL	General cognitive	Intelligence	
	CTBP2P3	Depression severity * hours spent watching television interaction		
	ST18	Psychotic experience (distressing)		
	TMPRSS11D	Cognitive performance		
	RPL31P9	Cigarette consumption * hours spent using computers interaction		
	EPB41L1			Intellectual developmental disorder, autosomal dominant 11 (MRD11)
	MYO9A		Neuroimaging Measurement	
	GBE1	Sensitivity to environmental stress and adversity	Attention Deficit Hyperactivity Disorder	
	DKK2	Resting-state electroencephalogram vigilance		
	ROBO1	Facial emotion recognition (fearful faces)	Waist Circumference	Neurooculorenal syndrome (NORS)
	TJP1	Response to antipsychotic treatment		
	SLIT2	Brain morphology (MOSTest)	Body Fat Percentage	
	LILRB1		High-Density Lipoproteins	
**GAD score**	*CHST9*	Severe depressive disorders in coronary artery disease	Attention Deficit Hyperactivity Disorder	
	TMPRSS11D	Cognitive performance		
	PIP4K2A	Sensitivity to environmental stress and adversity	Attention Deficit Hyperactivity Disorder	
	RPL7P45	Insomnia		
